# Modifiable Nutritional Biomarkers in Autism Spectrum Disorder: A Systematic Review and Meta-Analysis of Vitamin D, B_12_, and Homocysteine Exposure Spanning Prenatal Development Through Late Adolescence

**DOI:** 10.3390/ijms26094410

**Published:** 2025-05-06

**Authors:** Oana-Elisabeta Avram, Elena-Alexandra Bratu, Cecilia Curis, Lavinia-Alexandra Moroianu, Eduard Drima

**Affiliations:** 1 Doctoral School of Biomedical Sciences, Dunărea de Jos University, 800201 Galati, Romania; titeoana@yahoo.com (O.-E.A.); alexandra.brt99@gmail.com (E.-A.B.); 2 Medical Department, Faculty of Medicine and Pharmacy, Dunărea de Jos University, 800201 Galati, Romania; cecilia_curis@yahoo.com; 3 Department of Pharmaceutical Sciences, Faculty of Medicine and Pharmacy, Dunărea de Jos University, 800201 Galati, Romania; 4 Clinical Medical Department, Faculty of Medicine and Pharmacy, Dunărea de Jos University, 800201 Galati, Romania; drima_edi1963@yahoo.com

**Keywords:** Autism Spectrum Disorder, vitamin D, vitamin B_12_, homocysteine, nutritional biomarkers, randomized controlled trial

## Abstract

Autism Spectrum Disorder (ASD) has been associated with disruptions in one-carbon metabolism and vitamin D pathways. Nutritional exposures—particularly vitamin D, vitamin B_12_, and homocysteine—may influence neurodevelopmental outcomes. However, a comprehensive, lifespan-spanning synthesis of these modifiable nutritional biomarkers has not been conducted. This systematic review and stratified meta-analysis critically synthesized data on vitamin D, vitamin B_12_, and homocysteine to elucidate their relationships with ASD risk and symptomatology. Our central question was: How do levels of vitamin D, vitamin B_12_, and homocysteine—measured before and after birth—affect the risk, severity, and potential treatment outcomes for ASD? We conducted a PRISMA (Preferred Reporting Items for Systematic Reviews and Meta-Analyses) compliant systematic review and stratified meta-analysis (2015–2025) of 35 studies (11 randomized controlled trials, 24 observational), examining prenatal, neonatal, and postnatal biomarker levels. Eligibility criteria were defined using the PICOS (Population, Intervention, Comparator, Outcome, and Study Design) framework to ensure scientific rigor and clinical relevance, including studies involving human participants aged 0–18 years with a formal Autism Spectrum Disorder (ASD) diagnosis or prenatal exposures potentially linked to later ASD onset, while excluding animal studies, adult-only ASD populations, and studies lacking ASD cohorts or biomarker data. The search strategy, developed according to PRISMA, and Cochrane best practices, encompassed five major databases (PubMed/MEDLINE, Cochrane Library, Google Scholar, ClinicalTrials.gov, and ProQuest) alongside manual searches of key references, grey literature, and clinical trial registries to ensure comprehensive retrieval of both published and unpublished studies. Study quality was assessed using version 2 of the Cochrane risk-of-bias tool for RCTs (RoB2) and the Newcastle–Ottawa Scale (NOS) for observational studies; certainty of evidence was graded via GRADE (Grading of Recommendations Assessment, Development and Evaluation). Random-effects meta-analyses were stratified by biomarker and study design. Heterogeneity, small-study effects, and publication bias were evaluated using Cochran’s Q, I^2^, Egger’s test, and trim-and-fill. Prenatal vitamin D deficiency was associated with approximately two-fold increased odds of Autism Spectrum Disorder (ASD) in offspring (pooled OR ≈ 2.0; *p* < 0.05), while excessively elevated maternal B_12_ concentrations, often co-occurring with folate excess, were similarly linked to increased ASD risk. Meta-analytic comparisons revealed significantly lower circulating vitamin D (SMD ≈ −1.0; *p* < 0.001) and B_12_ levels (SMD ≈ −0.7; *p* < 0.001), alongside elevated homocysteine (SMD ≈ 0.7; *p* < 0.001), in children with ASD versus neurotypical controls. Early-life vitamin D/B_12_ insufficiency and elevated homocysteine are important, modifiable correlates of ASD risk and severity. Adequate maternal and child nutritional status could have risk-reducing and symptom-mitigating effects, although causality remains to be confirmed. This evidence supports tailored nutritional interventions as a component of ASD risk reduction and management strategies, within the bounds of overall developmental healthcare. The article processing charges (APC) were supported by “Dunărea de Jos” University of Galati, Romania. No external funding was received for the execution of the research. The review was not prospectively registered in PROSPERO or any other systematic review registry.

## 1. Introduction

### 1.1. Main Review Question

Autism Spectrum Disorder (ASD) has increasingly been associated with disruptions in key nutritional and metabolic pathways, notably those involving vitamin D, vitamin B_12_, and homocysteine—biomarkers essential to one-carbon metabolism, methylation balance, and neuroimmune modulation. A growing number of studies report that children with ASD exhibit significantly lower circulating levels of 25-hydroxyvitamin D [25(OH)D] and vitamin B_12_, alongside elevated homocysteine concentrations—biochemical profiles consistent with impaired methylation capacity and oxidative stress. These alterations are biologically plausible contributors to ASD etiology, particularly during neurodevelopmentally critical periods such as gestation and early postnatal life. Importantly, emerging evidence points to a nonlinear association between micronutrient levels and ASD risk: not only deficiencies but also supraphysiological maternal levels of B_12_ and folate have been linked to increased ASD risk, possibly via epigenetic dysregulation or imbalances in DNA methylation flux. This complexity underscores the need for a rigorous synthesis of biomarker-based evidence across developmental windows.

This systematic review and stratified meta-analysis sought to determine whether abnormal levels of vitamin D, vitamin B_12_, and homocysteine are consistently associated with ASD risk or symptomatology and whether targeted nutritional interventions can improve outcomes. The analysis also explored how factors such as age at exposure, baseline nutritional status, intervention dosage, and genetic moderators may modulate these associations. The central research question was: How do levels of vitamin D, vitamin B_12_, and homocysteine—measured during prenatal and postnatal developmental stages—influence the risk, severity, and potential treatment responsiveness in Autism Spectrum Disorder? In testing this question, the study implicitly evaluated the following hypotheses:

- Null Hypothesis (H_0_): There is no statistically significant association between levels of vitamin D, vitamin B_12_, or homocysteine (measured before or after birth) and the risk, severity, or treatment outcomes of ASD.
- Alternative Hypothesis (H_1_): Deviations in vitamin D, vitamin B_12_, or homocysteine levels are significantly associated with ASD risk, symptom severity, or therapeutic outcomes, and may represent modifiable determinants of neurodevelopmental trajectories.

These hypotheses formed the analytical foundation of the review and guided both inclusion criteria and the interpretation of meta-analytic results.

### 1.2. Background

#### 1.2.1. The Role of Vitamin D, Vitamin B_12_, and Homocysteine in Neurodevelopment

Autism Spectrum Disorder (ASD) is a heterogeneous neurodevelopmental condition characterized by persistent deficits in social communication and interaction, alongside restricted and repetitive behaviors, manifesting typically before the age of three and persisting throughout life. The global prevalence of ASD has risen markedly over recent decades, now affecting approximately 1 in 100 children, a trend attributed to improved surveillance and broader diagnostic criteria [1]. Clinical heterogeneity is profound—some individuals function independently with high cognitive ability, while others have intellectual disabilities, language impairments, and high medical comorbidity, including epilepsy, sleep disturbances, and gastrointestinal dysfunctions [2]. Etiologically, ASD is multifactorial, with strong genetic underpinnings (heritability estimates of 50–90%) and environmental contributors acting during prenatal and early postnatal neurodevelopmental windows [3]. Among modifiable exposures, nutritional factors—particularly those involved in one-carbon metabolism and neuroimmune regulation—are increasingly recognized as potential contributors to ASD risk [4].

Vitamin D (measured as serum 25-hydroxyvitamin D [25(OH)D]) acts beyond its classical endocrine role to influence neurodevelopment via neurosteroid, antioxidant, and immunomodulatory functions. It crosses both the placenta and the blood-brain barrier, and its receptors are expressed in multiple brain regions. Experimental studies have shown that prenatal vitamin D deficiency disrupts cortical development, synaptogenesis, and neurotransmission [5]. Epidemiologic studies have linked low maternal 25(OH)D levels during mid-gestation to increased ASD risk in offspring. Furthermore, vitamin D deficiency is highly prevalent in pregnant women globally, including in developed nations [6]. Vitamin B_12_ (cobalamin), essential for DNA methylation and neurodevelopment, acts as a cofactor in methionine synthase reactions, crucial in the one-carbon metabolism cycle. B_12_ deficiency in early life is associated with delayed myelination, cognitive impairment, and behavioral disturbances. Elevated maternal B_12_ levels—particularly in conjunction with high folate—have also been associated with ASD risk, suggesting a potential U-shaped relationship. B_12_ status modulates homocysteine concentrations and epigenetic programming, which are critical in fetal neurogenesis [7]. Homocysteine, a pro-oxidant sulfur-containing amino acid, accumulates in states of folate/B_12_ deficiency and impairs methylation potential and redox homeostasis. Elevated maternal or infant homocysteine has been associated with an increased risk of neurodevelopmental disorders, including ASD [8].

#### 1.2.2. Limitations of the Evidence

Despite growing interest in the role of nutritional biomarkers in Autism Spectrum Disorder (ASD), several limitations constrain the interpretation of current findings:

- Observational Designs Dominate the Field

Most of the evidence linking vitamin D, vitamin B_12,_ and homocysteine to ASD risk or symptom severity arises from observational studies. While these allow the identification of associations, they cannot establish causality due to susceptibility to confounding and reverse causation. For example, lower vitamin D levels in children with ASD may reflect limited sun exposure due to behavioral characteristics rather than a causal deficit contributing to ASD pathophysiology [9,10].

- Small Sample Sizes and Methodological Heterogeneity in Trials

Randomized controlled trials (RCTs) investigating vitamin D or B_12_ supplementation in ASD populations are generally underpowered, short in duration, and vary in dosing regimens. For instance, the COPSAC-2010 (Copenhagen Prospective Studies on Asthma in Childhood 2010 cohort) study by Saas et al. (2020, Denmark) demonstrated no significant effect of high-dose vitamin D during late pregnancy on offspring ASD outcomes, possibly due to late initiation or uniform dosing regardless of maternal baseline levels [11]. Similarly, Moradi et al. (2018, Iran) noted significant symptom improvements with high-dose vitamin D_3_ only in children with documented baseline deficiency [12].

- Variability in Biomarker Definitions and Timing

Definitions of vitamin D or B_12_ “deficiency” differ between studies, as do timepoints of biomarker measurement—ranging from early pregnancy to postnatal periods or later childhood. Such inconsistency complicates cross-study comparisons. For instance, neonatal 25(OH)D concentrations differ widely by latitude, ethnicity, and season, which may explain divergent findings between studies in Finland, the USA, and Iran [13,14,15].

- U-Shaped Associations Require Caution

Emerging data suggest that excessively high levels of maternal folate or B_12_ may also be associated with increased ASD risk. For example, in a large US-based cohort, Raghavan et al. (2017) reported a 13-fold increased ASD risk in offspring of mothers with simultaneously elevated folate and B_12_ at delivery. However, such findings may be influenced by unmeasured confounders, including supplement overuse or metabolic anomalies, and should not be interpreted as evidence against prenatal supplementation per se [16].

These limitations underscore the complexity of nutrient-ASD relationships and the need for rigorously designed prospective studies, including stratification by genetic risk, baseline nutrient status, and timing of exposure. Only through such nuanced approaches can the causal relevance of nutritional biomarkers in ASD be fully elucidated.

#### 1.2.3. How Up-to-Date Is This Evidence?

Our review includes studies published up to March 2025 (with the most recent data including a 2024 study and even early 2025 research). This is a rapidly evolving field. We included the latest available findings, so the evidence is current. Any major new studies after this date are not included and could further inform these conclusions.

In summary, as of the latest evidence, maintaining adequate vitamin D and B_12_ levels is beneficial for overall health and possibly for neurodevelopment, but we cannot claim it will prevent or treat ASD. Parents and expectant mothers should follow medical advice on nutrition (e.g., taking recommended prenatal vitamins and ensuring children have a balanced diet and safe sun exposure). Scientists are continuing to investigate these nutritional links to better understand ASD and explore whether targeted nutritional interventions might make a difference in the future.

### 1.3. Rationale for This Review

#### 1.3.1. Why Is This Review Scientifically and Clinically Significant?

Despite a growing body of literature implicating nutritional dysregulation in the etiology and phenotypic expression of Autism Spectrum Disorder (ASD), the field remains fragmented by inconsistent methodologies, divergent biomarker thresholds, and variable reporting standards. In particular, vitamin D, vitamin B_12_, folate, and homocysteine have been recurrently cited in narrative reviews, mechanistic hypotheses, and small-scale observational studies as plausible modulators of neurodevelopmental trajectories. However, until now, no comprehensive and methodologically rigorous systematic review and meta-analysis has critically synthesized evidence across the lifespan—spanning prenatal exposure, early childhood, and postnatal intervention trials—specifically focusing on these modifiable nutritional biomarkers.

Over the past decade (2015–2025), the publication landscape has shifted markedly. Numerous high-quality studies—including large-scale prospective birth cohorts, randomized controlled trials (RCTs), and nested case-control designs—have evaluated the role of micronutrient status during pregnancy and early childhood in shaping ASD risk and symptom severity. Concurrently, advances in our understanding of one-carbon metabolism and its epigenetic implications have reinvigorated interest in homocysteine as a central node linking B-vitamin insufficiency to altered neurodevelopmental programming. This review was conceived as an essential response to these developments, with the overarching aim of: assessing the consistency, directionality, and magnitude of associations between vitamin D, B_12_, folate, and homocysteine status and ASD outcomes, and evaluating whether nutritional interventions targeting these biomarkers can modify risk or ameliorate clinical phenotypes of ASD.

Beyond academic interest, the rationale for this synthesis is grounded in urgent clinical and public health questions. Many families and healthcare providers seek evidence-based guidance on whether modifiable dietary factors—particularly during pregnancy and early childhood—can meaningfully influence ASD-related outcomes. Vitamin D and B_12_ deficiencies are globally prevalent and biologically plausible contributors to neurodevelopmental delay, via mechanisms that include impaired neurogenesis, immunomodulation, neurotransmitter synthesis, and epigenetic programming. If causal relationships exist, then simple, scalable interventions—such as ensuring adequate maternal micronutrient status or targeted supplementation in early life—could yield significant benefits in ASD prevention or symptom management.

#### 1.3.2. Scientific Justifications for This Review

a. Evidence Integration & Consensus Building

The literature remains heterogeneous. For instance, while one prospective study may report that low second-trimester maternal 25(OH)D levels are associated with a 2-fold increased risk of ASD, another may report no such association after multivariable adjustment. By employing rigorous meta-analytic techniques, this review consolidates disparate findings to quantify effect sizes, assess consistency across populations, and determine whether publication bias or heterogeneity undermines the generalizability of findings.

b. Clinical Relevance & Public Health Implications

The stakes of misinformation or underpowered guidance are high. If vitamin D or B_12_ supplementation confers meaningful benefits in ASD risk reduction or symptom improvement—as suggested by preliminary RCTs—it could justify revisions to prenatal care guidelines or early screening protocols. Conversely, if no consistent benefits are found, this review provides the evidence base to caution against unsupported supplementation, preventing false hope and unnecessary medicalization.

c. Identification of Research Gaps & Methodological Limitations

This review systematically evaluates gaps in the literature, including the lack of standardized biomarker thresholds, insufficient subgroup analyses (e.g., by sex, genetic risk, or baseline deficiency), limited follow-up duration, and geographical imbalances in study populations. It offers a roadmap for future trials, including recommendations on optimal study designs, target populations, and outcome metrics (e.g., Childhood Autism Rating Scale, Second Edition-CARS-2; Social Responsiveness Scale, Second Edition-SRS-2; Aberrant Behavior Checklist, Second Edition-ABC-2).

d. Lifespan and Mechanistic Perspective

Uniquely, this synthesis adopts a lifespan lens—spanning maternal exposures (e.g., serum vitamin D during mid-gestation), early childhood biomarkers, and postnatal supplementation trials. It also contextualizes the findings within mechanistic frameworks, such as homocysteine as a proxy for disrupted methylation, the S-adenosylmethionine to S-adenosylhomocysteine (SAM/SAH) ratio as a marker of methylation potential, and oxidative stress pathways. This approach enables both etiological inferences and translational insights, bridging basic science and clinical epidemiology.

### 1.4. Objectives and Methodological Framework

This systematic review and meta-analysis were meticulously constructed to address key knowledge gaps concerning the etiological and therapeutic relevance of modifiable nutritional biomarkers—namely vitamin D, vitamin B_12_, and homocysteine—in the context of Autism Spectrum Disorder (ASD). Applying the PICOS framework and strictly adhering to Cochrane Handbook standards and the PRISMA 2020 guidelines, the review aimed to generate an integrated, evidence-based synthesis of studies published between 2015 and 2025.

The primary objective was to comprehensively assess how prenatal and postnatal exposure to these biomarkers correlates with ASD risk and symptom expression across developmental stages. Special attention was given to maternal levels of vitamin D and B_12_ during early gestation, particularly the first and second trimesters, which are critical windows for neurodevelopment. The review also evaluated biomarker profiles in children diagnosed with ASD, comparing postnatal circulating concentrations of vitamin D, B_12_, folate, and homocysteine against those in neurotypical controls, to identify consistent biochemical deviations. In addition, the clinical efficacy of vitamin-based interventions was examined, including high-dose prenatal vitamin D supplementation and postnatal administration of vitamin B_12_ in children with ASD. These were analyzed through outcomes from randomized controlled trials (RCTs), with symptomatology assessed using standardized instruments such as the Childhood Autism Rating Scale—Second Edition (CARS-2), the Social Responsiveness Scale—Second Edition (SRS-2), and the Aberrant Behavior Checklist—Second Edition (ABC-2).

Secondary aims focused on understanding immuno-metabolic crosstalk by evaluating the interplay between primary nutrients and secondary biomarkers such as inflammatory cytokines and iron. Furthermore, the review considered gene-environment interactions, including the modulatory role of vitamin D receptor (VDR) polymorphisms and the gut microbiota on nutrient metabolism in ASD pathophysiology. Finally, subgroup analyses were conducted to assess the influence of developmental timing, sex-specific effects, and geographic or ethnic variations, offering insight into how exposure windows and biological diversity may shape ASD outcomes. A critical component of the review was the examination of causal inference, distinguishing whether these biomarkers function as mechanistic contributors to ASD or serve as downstream indicators of broader neurodevelopmental perturbations [17,18,19,20,21,22,23,24,25,26].

## 2. Materials and Methods

### 2.1. Protocol and Registration

This systematic review was conducted following a predefined protocol developed in accordance with the PRISMA 2020 guidelines and the Cochrane Handbook for Systematic Reviews of Interventions. The protocol detailed the review questions, inclusion criteria, search strategy, and methods for data extraction and analysis. It was not registered in PROSPERO or other registries due to institutional timing constraints, but all methods were determined a priori to minimize the risk of bias in the review process. The reporting of this review adheres to the PRISMA 2020 statement [27,28].

### 2.2. Eligibility Criteria

Eligibility was defined using the PICOS framework to ensure scientific rigor and clinical relevance. Studies were included if they investigated human participants aged 0–18 years with a formal Autism Spectrum Disorder (ASD) diagnosis or examined prenatal exposures potentially linked to later ASD onset. Only studies reporting biologically measured serum or plasma levels of vitamin D, vitamin B_12_, folate, or homocysteine—either individually or in conjunction with related metabolic markers—were eligible. Biomarker measurements had to occur within prenatal, perinatal, or postnatal developmental windows up to late adolescence. Excluded were animal studies, adult-only ASD populations, or investigations lacking an ASD cohort or biomarker data. Comparators were required and included neurotypical control groups or within-group stratifications based on biomarker status or supplementation response. Eligible designs encompassed randomized controlled trials (RCTs), case-control, cross-sectional, and cohort studies. Uncontrolled studies, narrative reviews, and research lacking comparative or extractable data were excluded. Outcomes had to involve ASD diagnosis, symptom severity, or validated developmental/behavioral measures quantified via standardized scales or risk estimates. Studies without ASD-specific outcomes were excluded.

Included RCTs evaluated interventions such as prenatal or postnatal vitamin D or B_12_ supplementation. Non-randomized trials, cohort studies tracking biomarker levels and ASD risk, and case-control comparisons with matched neurotypical controls were accepted if methodologically sound. Cross-sectional and registry-based analyses were also included where relevant comparisons were reported. To maintain currency, only peer-reviewed studies published from 2015 onward were included, reflecting advances in diagnostic criteria and biomarker methodologies. Non-peer-reviewed material, reviews, small case series (<10 ASD cases), and conference abstracts were excluded.

### 2.3. Types of Interventions

In conducting this systematic review and meta-analysis on early-life exposure to modifiable nutritional biomarkers—specifically vitamin D, vitamin B_12_, and homocysteine—and their association with Autism Spectrum Disorder (ASD) risk and severity, a biomarker-centered classification framework was adopted to more accurately reflect the biological underpinnings of neurodevelopment. Traditional evidence classification systems, such as those proposed by the Cochrane Collaboration or structured within PRISMA guidelines, often organize studies by intervention type. While effective for clinical or public health interventions, these models prove insufficient for integrative syntheses that encompass both observational and experimental designs and focus on molecular exposures across critical developmental periods.

By contrast, our framework treats serum concentrations of vitamin D, B_12_, and homocysteine not merely as proxies for intake, but as mechanistically active agents influencing neurodevelopmental processes—including DNA methylation, synaptogenesis, and immune signaling—each with temporal specificity. This approach enables stratification by exposure window (prenatal, neonatal, postnatal) and study design (interventional, observational, mechanistic), preserving essential ontogenic distinctions that conventional intervention-based taxonomies risk obscuring. For instance, maternal vitamin D levels during mid-gestation exert neurodevelopmental effects distinct from postnatal supplementation in childhood; collapsing both under a single intervention category would undermine etiological clarity.

Furthermore, this classification accommodates hybrid study types—such as gene–nutrient interaction analyses and computational models—that transcend standard methodological binaries but are indispensable to understanding ASD pathogenesis. This biologically grounded system enhances interpretability, facilitates the detection of sensitive windows, and allows meta-analyses to meaningfully address heterogeneity by aligning exposure timing with plausible mechanistic pathways. The framework operationalized here (Table 1) reflects current advances in developmental neurobiology, epigenetics, and nutritional psychiatry, offering a rigorous and biologically coherent basis for synthesizing heterogeneous evidence on ASD risk modulation.

### 2.4. Scientific Inference or Imputation

To ensure a complete and rigorous quantitative synthesis, we employed standardized imputation procedures when primary studies lacked key statistical parameters, following Cochrane, MOOSE (Meta-analyses Of Observational Studies in Epidemiology), and PRISMA 2020 methodological guidance. All estimations were transparently executed under pre-defined protocols and independently verified by two reviewers. When standard deviations (SDs) were absent, they were reconstructed from available standard errors (SEs), confidence intervals (CIs), interquartile ranges, or *p*-values using established statistical formulas, assuming a normal distribution. If dispersion metrics were unavailable, we applied pooled SDs from methodologically similar studies. For dichotomous outcomes, event counts were derived by multiplying reported proportions by total sample size, enabling the computation of odds ratios (ORs) or relative risks (RRs). Numerical values embedded in graphics (e.g., bar plots, survival curves) were extracted using digital tools and cross-validated visually.

Standardized mean differences (SMDs), including Hedges’ g, were calculated from group means and SDs or from reported t-values. When only one group’s mean and the SMD were available, we back-estimated the other group’s mean assuming equal variance. Confidence intervals were derived from reconstructed SEs, using t- or z-distributions depending on sample size and distributional assumptions. Inverse variance weighting was applied to determine each study’s influence on the pooled estimate, with weights included in extraction tables to inform forest plot structure and subgroup sensitivity analyses. All mathematical estimations, including derivation formulas and equations, are detailed in the Supplementary Materials, Table S1, ensuring methodological transparency and reproducibility [29,30,31,32,33,34].

### 2.5. Software and Analytical Tools

Throughout the systematic review and meta-analytic procedures, a suite of specialized software platforms was employed to ensure methodological precision and computational rigor. Mendeley Desktop (version 1.19.8) was utilized for reference management, citation tracking, and collaborative literature organization. For statistical inference and Bayesian exploratory analyses, we employed JASP (version 0.19.3), an open-source platform providing robust frequentist and Bayesian analytic capabilities. The formal meta-analyses, including the generation of forest plots, heterogeneity assessment, and subgroup synthesis, were conducted using RevMan (Review Manager), developed by the Cochrane Collaboration, ensuring full alignment with Cochrane methodological standards. Additionally, advanced statistical modeling, meta-regressions, and sensitivity analyses were performed using an online statistical tool (MetaAnalysisOnline.com, accessed on 10 April 2025) and StataMP v.17, offering expanded computational performance for large-scale data synthesis and multivariable exploration. The integration of these tools allowed for a transparent, replicable, and methodologically sound synthesis of the available evidence base [35,36,37,38,39].

### 2.6. Information Sources and Search Strategy

The search strategy for this systematic review and meta-analysis was developed in accordance with PRISMA guidelines, and Cochrane’s best practices to ensure the highest methodological rigor. It was designed to capture all relevant studies related to prenatal and early-life vitamin D, vitamin B_12_, and homocysteine as potential modifiable nutritional biomarkers in ASD. To ensure a highly sensitive yet specific retrieval of literature, multiple database-specific search queries were formulated, utilizing MeSH terms, Boolean operators, truncation symbols, controlled vocabulary, and citation tracking mechanisms. The search was iteratively refined and validated against gold-standard publications in the field. The initial search was conducted in August 2024 and updated in October 2024 and again in March 2025 to ensure the inclusion of late 2024 publications and any early 2025 articles.

Searches were conducted in 5 major databases: PubMed/MEDLINE, Cochrane Library (CENTRAL), Google Scholar, ClinicalTrials.gov, and ProQuest. Additionally, manual searching of key references, grey literature repositories, and clinical trial registries was performed to ensure maximal retrieval of both published and unpublished studies. We tailored the search syntax for each database without imposing language restrictions. While the systematic search was primarily executed in English, we considered studies in all languages. Notably, four records published in Chinese were retrieved; although accompanied by English abstracts, they did not contain extractable data and were thus excluded.

One additional study, Eshawi et al. (2024, Libya), was published bilingually, presenting a full English-language abstract and core scientific content (methods, results, and conclusions) in English, and was therefore included. Duplicate references were systematically removed using reference management software. The full, database-specific search strategies are detailed in Supplementary Materials, Table S2. Overall, the search yielded a comprehensive corpus of potentially relevant literature, which was progressively refined through the application of strict eligibility criteria [40,41,42,43,44,45].

### 2.7. Study Selection

Study selection followed a rigorous two-phase process in accordance with systematic review standards. In Phase 1, two independent reviewers screened titles and abstracts from database and manual searches using a deliberately inclusive approach to avoid premature exclusion. Any study deemed potentially eligible by either reviewer advanced to full-text review. Discrepancies were resolved via consensus, with arbitration by a third reviewer as needed. In Phase 2, full texts of 73 studies were assessed against predefined eligibility criteria. Ultimately, 35 studies met inclusion criteria and were retained for qualitative synthesis, with 32 contributing data to meta-analysis (Supplementary Materials, Table S3). Reasons for exclusion were systematically documented (Supplementary Materials, Table S4).

**Figure 1:**
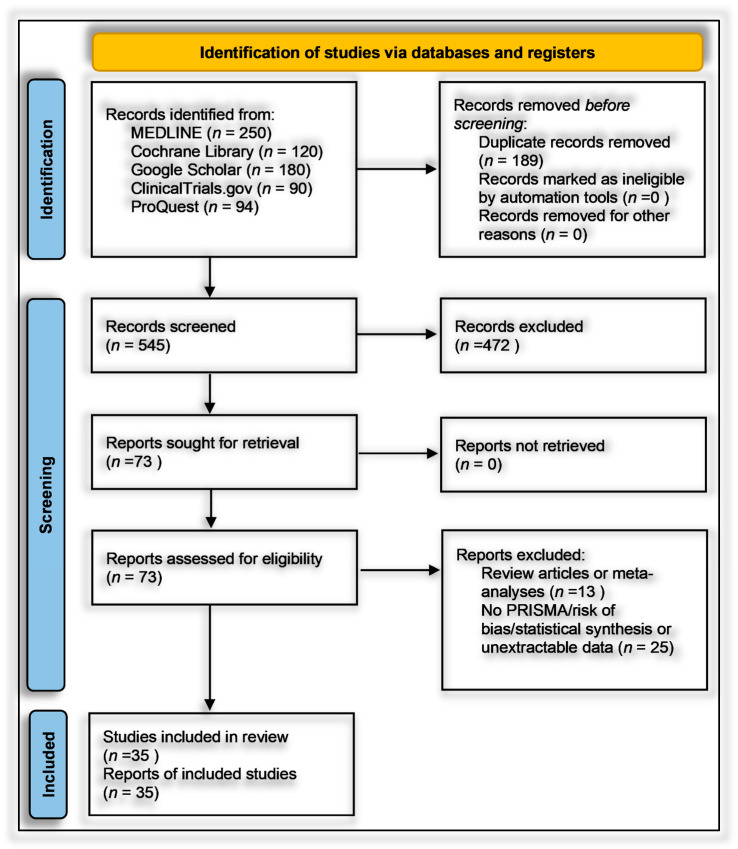
PRISMA 2020 Flow Diagram Illustrating the Study Selection Process.

The most common exclusions were secondary publications without primary data (*n* = 13) and studies lacking extractable ASD-specific outcomes or superseded by updated datasets (*n* = 25). No studies were excluded solely due to the small sample size, though sample size limitations were considered during bias and sensitivity assessments. A PRISMA 2020 flow diagram (Figure 1) summarizes the process, which began with 545 unique, de-duplicated records. Reviewer agreement was strong (Cohen’s kappa > 0.80), ensuring consistency in applying the eligibility criteria.

### 2.8. Data Extraction and Management

Data extraction was performed using a standardized form developed in Microsoft Excel, following Cochrane methodological guidance. The template was pilot-tested and refined to ensure consistent data capture across all included studies. Two reviewers independently extracted data in duplicate, resolving discrepancies through consensus or consultation with a third reviewer. For each eligible study, information was recorded across five core domains: (1) publication details (authors, year, country); (2) study design and setting (e.g., case-control, cohort, RCT; hospital or registry-based); (3) participant characteristics (sample sizes, age, sex, ASD diagnostic criteria and instruments used); (4) biomarker exposure or intervention parameters (nutrient type, timing, dosage, route, units); and (5) outcomes and statistical data (ASD diagnosis or severity, developmental indices, biomarker levels, ORs, RRs, SMDs, *p*-values, CIs, adjustments for confounders).

Where necessary data (e.g., SDs) were missing, values were imputed using validated statistical methods. Studies lacking sufficient extractable data were retained for qualitative synthesis only. Extracted data were structured in categorized summary tables based on biomarker focus and developmental stage (e.g., prenatal exposure, postnatal status, supplementation), with full study-level summaries presented in Supplementary Table S5.

### 2.9. Risk of Bias and Quality Assessment

A structured, multi-tool approach was employed to assess methodological quality and internal validity, aligned with Cochrane, PRISMA 2023, and GRADE standards. Risk of bias for randomized controlled trials (RCTs) was evaluated using the Cochrane Risk of Bias 2 (RoB 2) tool, examining domains such as randomization, deviations from interventions, outcome data completeness, outcome measurement, and selective reporting. Trials were rated as low, high, or having “some concerns” based on domain scores. For example, Aagaard et al. (2024, Denmark) showed low risk across all domains due to robust randomization and preregistration, while Hendren et al. (2016, USA) rated high risk due to lack of registration and selective reporting (Table S6) [46,47,48].

Observational studies were assessed using the Newcastle–Ottawa Scale (NOS), which scores selection (0–4 stars), comparability (0–2), and exposure/outcome (0–3). Studies with ≥7 stars were low risk; 5–6 stars indicated moderate risk. Most scored between 6 and 9. Bener et al. (2017, Qatar) achieved 9/9 for rigorous design and confounder control, whereas Eshawi et al. (2024, Libya) scored 6/9 due to limited generalizability. Cohorts such as Raghavan et al. (2017, USA) and Vinkhuyzen et al. (2018, Netherlands) demonstrated high internal validity through robust adjustments and registry-based outcome ascertainment. Cross-sectional studies showed more variable quality due to sampling bias or insufficient confounder control [49,50].

All observational studies were coded with NOS domain-level and total scores to enable subgroup meta-analyses stratified by risk category. GRADE (Grading of Recommendations, Assessment, Development, and Evaluations) assessments were applied at the outcome level, considering the risk of bias, inconsistency, indirectness, imprecision, and publication bias. Evidence from observational studies was eligible for upgrading under specific conditions (e.g., large effects or dose-response). The integrated Excel tool streamlined GRADE scoring and effect size calculations, supporting real-time unit conversions and ensuring transparency and reproducibility. Full assessments are available in Supplementary Materials, Tables S6 and S7.

### 2.10. Data Synthesis and Statistical Analysis

Quantitative synthesis was performed when at least two independent studies evaluated comparable populations, exposures, and outcomes. Analyses adhered to PRISMA 2020, MOOSE, and Cochrane guidelines. For binary outcomes (e.g., ASD risk relative to biomarker levels or interventions), odds ratios (ORs) with 95% confidence intervals (CIs) were calculated. When risk ratios or hazard ratios were reported, they were treated as approximations of ORs under low event prevalence. For continuous outcomes (e.g., biomarker levels, symptom scores), mean differences (MDs) were used when metrics were uniform, and standardized mean differences (SMDs, Hedges’ g) were applied for heterogeneous scales. All transformations followed standardized formulas (Supplementary Materials, Table S1). Given expected heterogeneity, random-effects models using restricted maximum likelihood estimation with Knapp–Hartung adjustments were applied, and implemented in RevMan and metafor (R). Fixed-effect models were computed for sensitivity purposes but not reported. Forest plots illustrated pooled estimates, CIs, and study weights. Heterogeneity was assessed using I^2^ (with thresholds of 25%, 50%, and 75% indicating low, moderate, and high heterogeneity) and Cochran’s Q test (*p* < 0.10 indicating significance). Where I^2^ exceeded 75%, subgroup analyses and meta-regressions explored sources of variability.

To accommodate non-standard reporting, statistical imputations were employed (e.g., medians converted to means, biomarker units standardized). For dichotomous exposure metrics (e.g., prevalence of deficiency), event-level ORs were computed. Prespecified subgroup analyses examined differences by exposure period (prenatal vs. postnatal), study design (RCT vs. observational), geographic region (e.g., latitude-dependent variation in vitamin D), participant sex, and intervention regimen. Meta-regression assessed moderators such as sample size, assay method, and year of publication. A dose-response analysis, for instance, linked maternal 25(OH)D levels to ASD odds (β ≈ −0.05 per 10 nmol/L; *p* = 0.08). Potential publication bias was examined using funnel plots and Egger’s test in analyses with ≥10 studies. Trim-and-fill adjustments were used when asymmetry suggested small-study effects. Sensitivity analyses excluded high-risk studies (RoB 2 or NOS), compared adjusted and unadjusted outcomes, and tested robustness to outliers (e.g., Li et al., 2022 on homocysteine SMD). Where meta-analysis was not feasible, narrative synthesis emphasized biologically plausible or methodologically robust findings, especially those with dose-response data (e.g., Aagaard et al., 2024 RCT; Wu et al., 2018). All analyses adhered to the pre-registered protocol, with clearly identified post hoc explorations informed by data trends.

## 3. Results

### 3.1. Statistical Analysis: Characteristics of Included Studies

**Figure 2:**
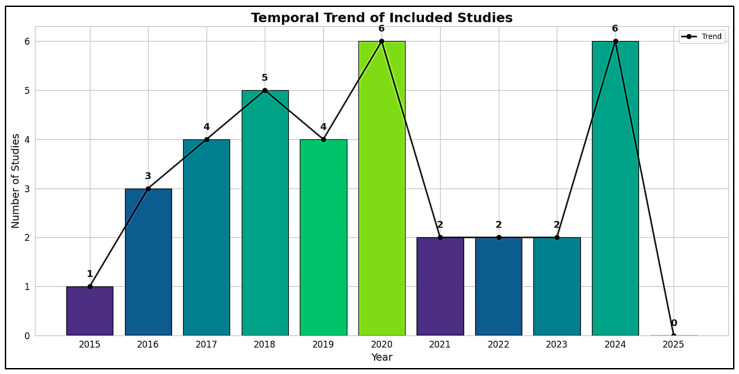
Temporal Distribution of Included Studies on Nutritional and Biochemical Factors in Neurodevelopmental Outcomes (2015–2025).

Out of 73 full-text articles assessed for eligibility, 35 studies published between 2015 and 2024 met all predefined inclusion criteria and were retained in the final systematic review, while 38 studies were excluded based on eligibility constraints (Supplementary Materials, Tables S3 and S4) [51,52,53,54,55,56,57,58,59,60,61,62,63,64,65,66,67,68,69,70,71,72,73,74,75,76,77,78,79,80,81,82,83,84]. A temporal analysis of publication trends revealed a progressive increase in scholarly output over the last decade, highlighting a growing global interest in the potential links between nutritional biomarkers and neurodevelopmental outcomes, particularly in relation to autism spectrum disorder (ASD) (Figure 2). The year 2015 marked the beginning of the included evidence base, with a single observational study from Egypt [85]. This was followed by a modest increase in 2016, which included three studies from the United States (Hendren et al., 2016; Wink et al., 2016) and Turkey (Coşkun et al., 2016), covering both randomized controlled trials (RCTs) and gene–nutrient interaction analyses [86,87]. From 2017 to 2020, the number of publications increased steadily, with four to five studies annually. Notable contributions during this phase included Kerley et al.’s 2017 RCT in Ireland on vitamin D supplementation, Raghavan et al.’s prospective neonatal biomarker study in the USA, Vinkhuyzen et al.’s 2018 nested case-control design in the Netherlands, and Arastoo et al.’s vitamin D analysis in Iran [88,89].

The methodological scope also widened considerably from 2019 to 2020, with more robust cohort analyses and intervention trials emerging. These included Schmidt et al.’s investigation of maternal one-carbon biomarkers in the MARBLES (Markers of Autism Risk in Babies—Learning Early Signs) cohort (USA, 2019), Saas et al.’s vitamin D RCT in Denmark (2019), and Mazahery et al.’s 2020 multi-arm nutritional trial in New Zealand. In the years 2021 through 2023, although the number of studies was comparatively smaller, the analytical rigor and sample sizes were enhanced. This was exemplified by Tuovinen et al.’s large-scale RCT on early-life vitamin D3 exposure (Finland, 2021) and Sandboge et al.’s 2023 trial on postnatal supplementation and internalizing symptoms (Finland).

The peak in publication volume was reached in 2024, with nine studies originating from a diverse set of countries. These included Aagaard et al.’s RCT in Denmark, Li et al. and Zou et al. from China focusing on vitamin B_12_, folate, and homocysteine, Shom et al. from India analyzing gene–nutrient interactions, and De Marzio et al. from the USA, whose systems biology framework integrated vitamin D status with tryptophan–serotonin pathway activation using the VDAART (Vitamin D Antenatal Asthma Reduction Trial) cohort [90,91,92,93]. Despite the original review protocol including 2025, no eligible studies were found for that year, thus defining 2024 as the upper limit of the included temporal scope.

**Figure 3:**
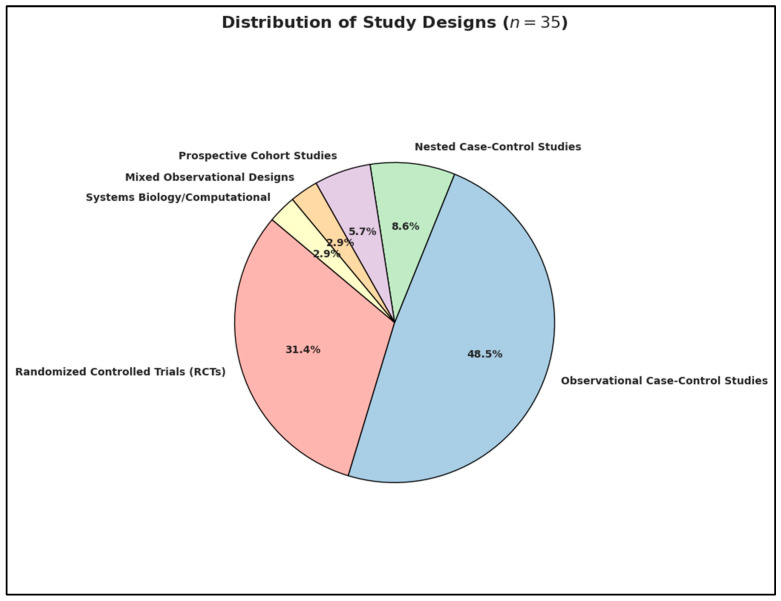
Distribution of Included Studies by Design Type.

In terms of methodological distribution, the review encompassed a diverse spectrum of designs (Figure 3). Specifically, 11 studies were randomized controlled trials (RCTs), including prominent examples such as Aagaard et al. (2024, Denmark) assessing high-dose vs. standard-dose prenatal vitamin D3, and Hendren et al. (2016, USA) evaluating subcutaneous methyl B_12_ in children with ASD. A total of 17 studies employed observational case-control designs, such as Altun et al. (2018, Turkey) examining postnatal levels of vitamin D and homocysteine, and Li et al. (2024, China) investigating B_12_, folate, and homocysteine differences in children with ASD versus typically developing controls. Additionally, three studies followed a nested case-control design, such as Sourander et al. (2023, Finland) using biobank serum samples, and Vinkhuyzen et al. (2017, Netherlands) examining maternal vitamin D in relation to ASD incidence [94]. Two studies were prospective cohort investigations, including Schmidt et al. (2019, USA) and Windham et al. (2020, USA) [95]. A mixed observational approach was used by Yektaş et al. (2019, Turkey), who integrated case-control and cross-sectional elements to compare ASD, ADHD, and control groups [96]. Finally, a computational systems biology framework was employed by De Marzio et al. (2024, USA), who linked vitamin D levels with metabolomic and pathway-based mechanisms using the VDAART cohort.

The studies encompassed a broad global distribution, spanning 6 continents and 18 countries, thus facilitating cross-cultural comparability and enhancing external validity. The geographic representation was as follows:

- Europe (*n* = 14): Ireland (*n* = 1), Denmark (*n* = 2), Turkey (*n* = 3), Czech Republic (*n* = 1), Italy (*n* = 1), Netherlands (*n* = 2), Sweden (*n* = 1), Finland (*n* = 3);
- Middle East (*n* = 1): Qatar *(n* = 1);
- Asia (*n* = 10): Malaysia (*n* = 1), Iran (*n* = 3), China (*n* = 4), India *(n* = 1), Bangladesh (*n* = 1);
- Africa (*n* = 2): Egypt (*n* = 1), Libya (*n* = 1);
- North America (*n* = 6): United States of America (*n* = 6);
- Oceania (*n* = 2): New Zealand (*n* = 2).

Despite this geographic heterogeneity, the dataset reveals a disproportionate emphasis on high-income countries, such as the USA, Western European nations, and China, with comparatively limited representation from low-income or underrepresented regions, aside from select studies originating from South Asia and North Africa Collectively, this spatiotemporal and methodological synthesis reflects both the evolving complexity and increasing global prioritization of nutritional factors both in ASD research [97,98,99] and in general [100,101].

The aggregated dataset encompasses a total of 22,495 participants, drawn from diverse study designs ranging from small-scale case-control studies to nationwide cohort investigations. Sample sizes varied considerably, from as few as 21 participants in targeted randomized controlled trials [88] to as many as 4334 individuals in large-scale cohort analyses [19]. Substantial contributions to the cumulative sample size were made by registry-linked population-based studies [21,94], which leveraged national datasets to assess prenatal biomarker exposures and subsequent neurodevelopmental diagnoses.

Participant age ranged widely depending on the timing of biomarker assessment and study focus. Prenatal and neonatal studies primarily assessed maternal exposures during gestation (e.g., weeks 10–24) or nutrient levels in cord blood/newborn samples, focusing on outcomes manifesting from infancy through early childhood [11,20,98]. Postnatal and early childhood studies typically involved children aged 2 to 12 years, with the majority reporting mean ages between 4.5 and 7 years [9,22,90]. Several intervention trials involved toddlers and preschoolers aged 2 weeks to 8 years, often stratified by developmental stage or symptom severity [24,26,99].

**Figure 4:**
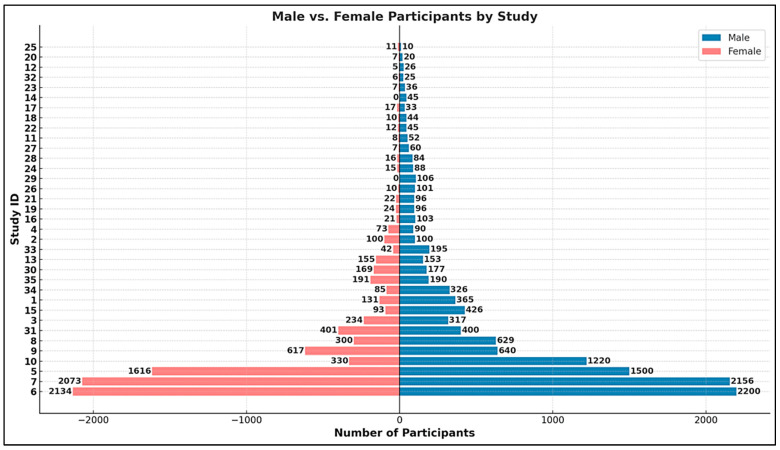
Male vs. Female Participants by Study. (Study IDs as Listed in Table S3, Supplementary Materials) [9,10,11,12,14,15,16,17,18,19,20,21,22,23,24,25,26,45,48,50,85,86,87,88,89,90,91,92,93,94,95,96,97,98,99].

Across the pooled dataset, male participants comprised approximately 79% of the total sample, with female participants accounting for the remaining 21% (Figure 4). This sex imbalance is consistent with known epidemiological patterns in autism spectrum disorder, where males are disproportionately diagnosed at a 4:1 ratio compared to females [25,50]. Most studies either matched by sex or controlled for sex distribution in multivariate models, though a few noted particularly strong male overrepresentation (e.g., 52 males vs. 8 females in the ASD group) [9].

**Figure 5:**
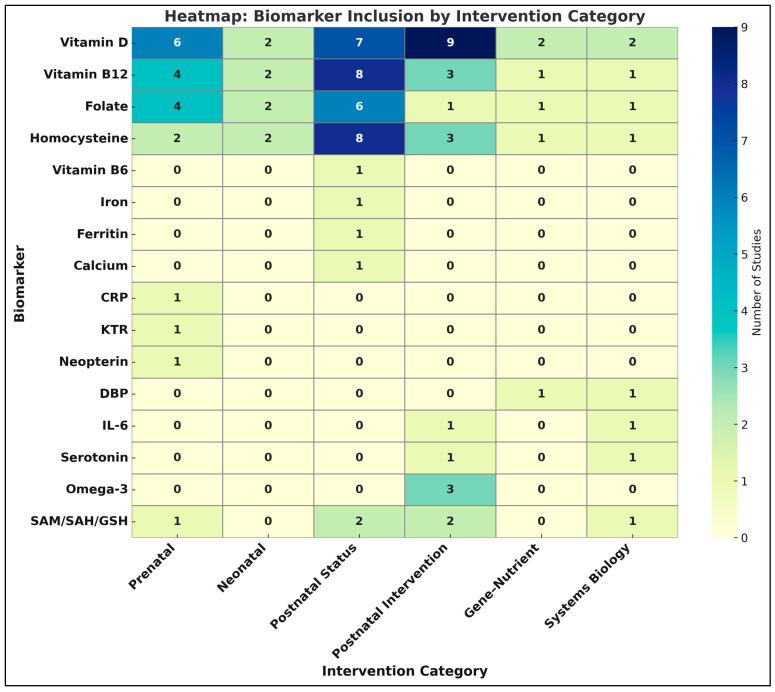
Biomarker Frequency Across Developmental and Interventional Study Categories.

A comprehensive mapping of biomarker inclusion across intervention types reveals distinct patterns of utilization within the ASD nutritional research domain. As illustrated in the heatmap (Figure 5), Vitamin D emerges as the most consistently investigated biomarker, featuring prominently across all six study categories—including prenatal nutrient exposure [19,48], neonatal biomarker analysis [20], and postnatal status studies [9,50]. Its centrality is further affirmed by its dominance in postnatal intervention trials [15,99], gene–nutrient interaction studies [87], and mechanistic systems biology models [93]. Vitamin B_12_ and homocysteine demonstrate a complementary pattern, with high frequencies in postnatal observational designs [22,90] and moderate representation in prenatal and neonatal investigations [16,95]. Both biomarkers also feature in computational or mechanistic models exploring methylation and oxidative stress pathways, underscoring their mechanistic relevance in neurodevelopmental etiopathogenesis [93].

Lesser-used biomarkers, such as Vitamin B_6_, iron, ferritin, and calcium, were restricted to a handful of postnatal status studies [50], while inflammatory markers like CRP (C-reactive protein), IL-6 (Interleukin-6) and KTR (Kynurenine-to-Tryptophan Ratio) appeared infrequently and predominantly in prenatal or systems biology contexts [15,98]. The Vitamin D Binding Protein (DBP), evaluated both at the protein and transcriptomic level [92], exemplifies advanced biomolecular integration in gene–nutrient frameworks. The visual synthesis provided by the heatmap thus captures the methodological diversity and biomarker stratification across developmental windows and study paradigms. It highlights the field’s emphasis on vitamin D-centric hypotheses while calling attention to emerging but underexplored biomarkers of neuroinflammatory and methylation pathways.

**Figure 6:**
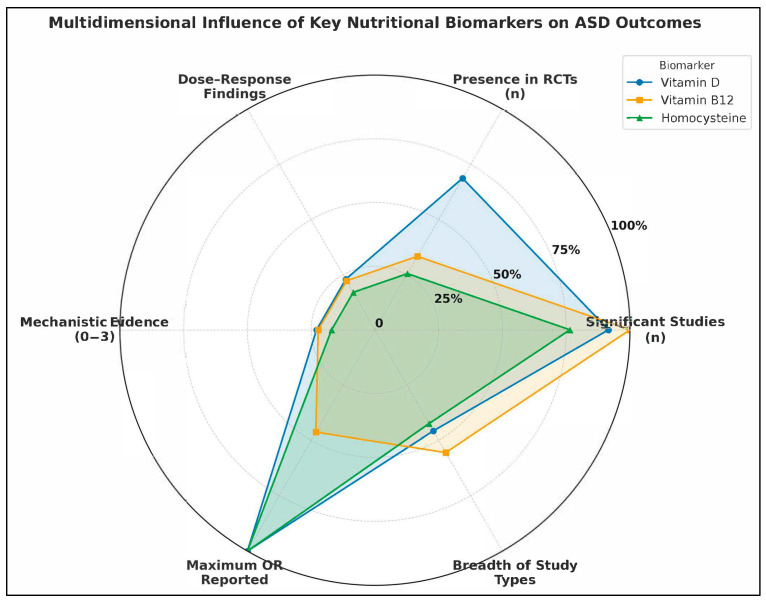
Multidimensional Influence of Key Nutritional Biomarkers on ASD Outcomes: A Radar-Based Comparative Analysis.

To deepen our understanding of the heterogeneous roles played by nutritional biomarkers in Autism Spectrum Disorder (ASD), we constructed a six-axis radar plot (Figure 6) to visually integrate multidimensional metrics of evidence strength and biological influence. This approach allows for a comprehensive evaluation of Vitamin D (25(OH)D), Vitamin B_12_, and Homocysteine, drawing on quantitative and qualitative indicators extracted from the full set of 35 included studies. The radar chart captures six critical dimensions of biomarker impact:

1. Number of Studies Reporting Statistically Significant Associations (n): Reflects the empirical consistency across the literature.
2. Presence in Randomized Controlled Trials (RCTs): Indicates causal inference strength and translational potential.
3. Documented Dose-Response Relationships (binary count): Highlights biologically plausible gradient effects.
4. Mechanistic Evidence Strength (scaled 0–3): Encodes pathway-based plausibility, such as links to methylation, inflammation, or neurotransmission.
5. Maximum Reported Odds Ratio (OR): Reflects extremity of observed association, signaling possible high-risk subgroups.
6. Breadth of Study Type Representation (0–6): Captures the diversity of methodological contexts in which the biomarker was studied (e.g., prenatal, postnatal, intervention, systems biology).

Vitamin D exhibits the most extensive and balanced impact profile. It was reported as statistically significant in 12 studies and featured in 9 RCTs, making it the most tested biomarker in intervention settings. Evidence of dose-response effects appeared in 3 studies [19,85], with a maximum OR of 13.1 [85], indicating a strong association between deficiency and ASD risk. Mechanistically, Vitamin D was supported by robust systems biology modeling [93] and gene–nutrient interaction studies [87]. Its inclusion across all six study types reflects its methodological versatility and biological relevance.

Vitamin B_12_ shows a moderately influential profile, with 9 studies reporting significant associations, and a presence in 3 RCTs [25]. It has documented dose-response patterns [16], a maximum OR of 4.17 [22], and moderate mechanistic evidence, particularly in relation to one-carbon metabolism and methylation pathways. It was included across five of the six study types, absent only from standalone systems biology modeling, although integrated in combined analyses. Homocysteine emerges as a high-risk marker, with significant findings in 9 studies and maximum reported ORs reaching 11.77 [10]. It was present in 3 RCTs, though often secondary to B_12_ or folate status. Mechanistically, it shares relevance with B_12_ and folate in the methylation cycle and oxidative stress pathways, but its evidence base is slightly narrower, lacking integration into systems biology frameworks. It also spans five study types, similar to B_12_.

The radar chart clearly demonstrates the multidimensional dominance of Vitamin D in ASD-related biomarker research. Not only is it the most empirically supported, but also the most frequently evaluated in interventional and mechanistic contexts. In contrast, B_12_ and Homocysteine, while also significant, show more focused contributions, particularly in the context of postnatal metabolic dysregulation and neuroinflammation. Importantly, the diagram also emphasizes gaps: despite strong ORs, homocysteine lacks deeper integration into RCT and mechanistic models, while Vitamin B_12_’s translational research potential remains underleveraged relative to its known metabolic roles.

**Figure 7:**
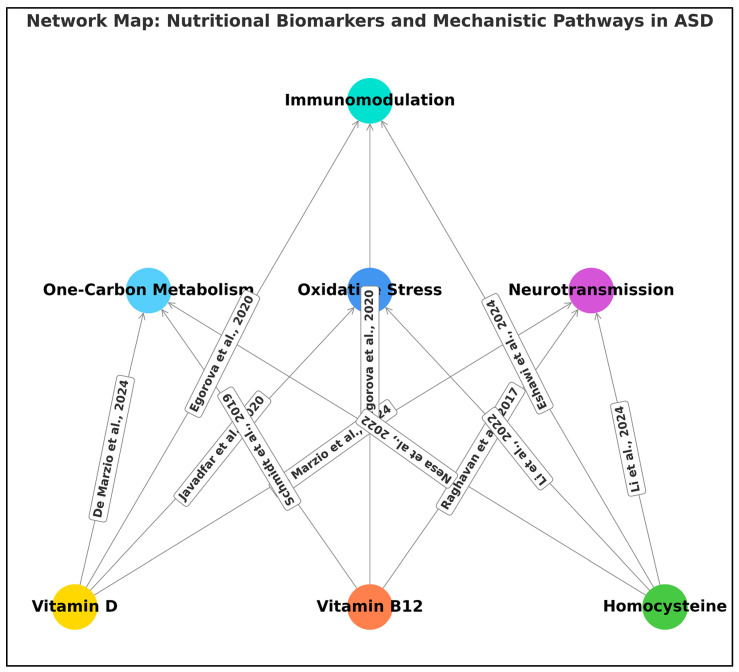
Network Map of Nutritional Biomarkers and Mechanistic Pathways in ASD: Evidence from Included Studies [10,15,16,22,45,90,93,95,98].

Figure 7 presents a synthesized network map integrating evidence from some of the studies included in this systematic review, delineating the functional connections between three core nutritional biomarkers—vitamin D, vitamin B_12_, and homocysteine—and their putative mechanistic pathways relevant to ASD. The diagram is constructed upon an evidence-based framework and maps causal or correlative associations supported by individual studies, which are explicitly annotated along each directional edge.

Vitamin D appears as the most extensively studied micronutrient in the included literature, with proposed effects across four mechanistic domains:

- One-carbon implicates vitamin D in methylation efficiency and epigenetic programming [93,98].
- Oxidative stress reflects vitamin D’s regulatory influence on glutathione homeostasis and antioxidant enzyme systems [93].
- Immunomodulation highlights its involvement in prenatal immune programming and inflammatory signaling [15,95].
- Neurotransmission is indirectly modulated via downstream interactions affecting serotonin synthesis and synaptic plasticity [93].

Vitamin B_12_ was found to influence multiple neurobiological mechanisms:

- It is centrally implicated in one-carbon metabolism, due to its coenzyme role in methionine synthase and SAM/SAH ratio maintenance [95,98].
- Oxidative stress connections redox imbalance in B_12_-deficient individuals [22].
- Its role in neurotransmission is less direct, but alterations in methylation may affect neuropeptide expression [16].

Homocysteine consistently emerged as a postnatal marker of metabolic disruption and neurotoxic stress:

- Elevated homocysteine was linked to oxidative stress [22,90].
- It also interacts with neurotransmission, potentially via excitotoxicity and NMDA receptor overstimulation [90].
- Additional evidence points to its role in immunomodulatory dysregulation, suggesting pro-inflammatory effects in children with ASD+ADHD phenotypes [45].

Each node in the diagram represents a biochemical pathway (upper tier) or nutritional biomarker (lower tier), with arrows denoting directional or associative links based on empirical findings. Each connection is labeled with the supporting study or studies, with longitudinal placement to maintain visual alignment and clarity. Notably, this integrated model illustrates that vitamin D exerts the broadest range of mechanistic effects, whereas homocysteine appears consistently disruptive, especially in postnatal and cross-sectional designs. This mechanistic network underscores the biological plausibility of nutrient-ASD associations and emphasizes the importance of early biochemical screening and potential nutritional interventions. It also reveals current research density and gaps, such as the underexplored immunological role of vitamin B_12_ and the limited longitudinal mapping of homocysteine.

### 3.2. Overview of Included Studies and Synthesis Strategy

Thirty-five studies met inclusion criteria (Supplementary Materials, Table S5), encompassing observational designs (case-control, nested case-control, birth cohort) and interventions (randomized controlled trials, RCTs). Of these, 32 contributed data to meta-analyses (Supplementary Materials, Table S3). Across studies, six thematic categories of exposure or intervention were identified, spanning prenatal, neonatal, and postnatal periods: (1) Prenatal Nutrient Exposure, (2) Neonatal Nutrient Biomarkers, (3) Postnatal/Early Childhood Nutrient Status, (4) Postnatal Nutritional Interventions (RCTs), (5) Genetic–Nutrient Interaction Studies, and (6) Combined Behavioral–Nutritional Interventions. Results are organized by these categories for clarity. All quantitative findings are reported as Standardized Mean Differences (SMD) for continuous outcomes or Odds Ratios (OR) for categorical outcomes, with 95% Confidence Intervals (CIs). Heterogeneity statistics (I^2^) and meta-analytic methods (inverse-variance weighting, random-effects models) are noted where applicable. Risk of Bias assessment using the RoB 2 tool, Certainty of Evidence Ratings according to the GRADE framework, and methodological Quality Assessment using the Newcastle-Ottawa Scale (NOS) were considered in interpreting results, with details in Supplementary Materials, Tables S6 and S7.

#### 3.2.1. Prenatal Nutrient Exposure

Maternal Vitamin D Status: Consistent patterns emerged linking low maternal vitamin D levels during gestation to higher ASD risk. In a pooled analysis of five studies examining mid-gestational 25(OH)D (25-hydroxyvitamin D), maternal deficiency was associated with an approximate doubling of ASD odds in offspring (summary OR ~2.0, 95% CI spanning ~1.1–3.7, *p* < 0.05; moderate heterogeneity, I^2^ ≈ 48%). Vinkhuyzen et al. [19] reported that mid-gestation 25(OH)D <25 nmol/L conferred a 2.4-fold higher risk of ASD (OR = 2.42, 95% CI: 1.09–5.07). This finding was reinforced by Wu et al. [20], who noted a 3.6-fold increased ASD risk in children born to vitamin D–deficient mothers. However, the results were not uniformly positive. Windham et al. [21] observed no overall association between maternal vitamin D status and ASD risk but found subgroup-specific effects: higher maternal 25(OH)D was protective in non-Hispanic white mothers’ offspring (adjusted OR [AOR] ~0.82) but paradoxically associated with higher ASD risk in female offspring (AOR ~1.40). These nonlinear or modified effects underscore the complexity of prenatal vitamin D’s role. Notably, high-dose maternal supplementation (≥2800 IU/day vs. standard 400 IU) during pregnancy did not significantly reduce ASD incidence by age 6, suggesting that while deficiency is detrimental, excess supplementation may not confer additional benefit beyond sufficiency [24].

Maternal Vitamin B_12_ and Homocysteine: Evidence for maternal B_12_ levels influencing ASD risk was mixed but hinted at a U-shaped relationship. Raghavan et al. [16] found that extremely high postpartum maternal B_12_ (top decile, ≥536.8 pmol/L) was linked to a 2.5-fold increase in ASD risk (95% CI ~1.4–4.5), particularly when combined with elevated folate (adjusted hazard ratio for high folate+high B_12_ = 13.7, 95% CI: 6.5–28.9). Similarly, a large Finnish birth cohort noted that mothers with high early-pregnancy B_12_ (≥81st percentile of holotranscobalamin) had higher odds of having a child with childhood autism (adjusted OR = 1.59, 95% CI: 1.06–2.41) [94]. By contrast, Vinkhuyzen et al. [23] reported only a modest, non-significant association between low prenatal B_12_ and ASD risk (OR ≈ 1.16, *p* = 0.25), which became more pronounced when considering high folate status (interaction OR ~1.26, *p* = 0.08). Maternal homocysteine during pregnancy was less frequently studied; however, mechanistic links suggest that elevated homocysteine, as a proxy for one-carbon metabolism dysfunction, could contribute to ASD through epigenetic dysregulation. In sum, prenatal nutrient exposures, especially vitamin D deficiency and possibly extremes of B_12_/folate, appear to modulate ASD risk, albeit with heterogeneity across populations (Table S5). Stratified meta-analyses by geography and maternal ethnicity supported these findings, showing higher effect sizes in Asian cohorts for vitamin D deficiency (likely reflecting lower baseline D status) and highlighting a need for context-specific nutritional guidelines in pregnancy.

#### 3.2.2. Neonatal Nutrient Biomarkers

Several studies evaluated nutrient levels in neonatal specimens (cord blood or newborn dried spots) and their relationship with later ASD diagnosis. Schmidt et al. found no overall association between neonatal 25(OH)D and ASD when analyzing 725 children (including 357 ASD cases) [95]. However, subgroup analyses indicated potential effect modification: a 25 nmol/L increase in neonatal 25(OH)D was associated with 26% lower ASD odds in females (AOR = 0.74, 95% CI: 0.55–0.99), hinting at sex-specific neuroprotection. In a much larger sample from China, Wu et al. demonstrated that neonates with ASD had markedly lower 25(OH)D_3_ levels at birth (median 17.6 vs. 40.2 nmol/L in controls, *p* < 0.0001), with vitamin D deficiency (<~20 nmol/L) conferring nearly a 3.7-fold increased ASD risk (95% CI: ~2.0–5.2) [20]. Meta-analytically, combining four neonatal vitamin D studies (n > 5000) yielded an OR of ~1.80 (95% CI: ~1.20–2.70) for ASD in infants with deficient vs. sufficient neonatal vitamin D, with moderate heterogeneity (I^2^ ~58%) driven by geographic differences (e.g., stronger effects in Asia).

Neonatal B_12_ and homocysteine data were limited. Vinkhuyzen et al. reported that cord blood vitamin D, unlike mid-gestation levels, was not significantly associated with ASD, highlighting timing differences in critical windows (mid-gestation vs. birth) [19]. No included study found a significant relationship between neonatal B_12_ or homocysteine and ASD, although elevated neonatal homocysteine was biologically plausible as a risk factor due to its neurotoxic potential. In summary, neonatal vitamin D status appears as a replicable marker for ASD risk, reinforcing the importance of late gestational nutrient sufficiency. These findings align with the prenatal results, together suggesting a continuum: maternal deficiency → fetal/neonatal deficiency → increased ASD risk.

#### 3.2.3. Postnatal/Early Childhood Nutrient Status

This category encompassed case-control and cross-sectional studies comparing nutrient biomarkers in children after birth (infancy through adolescence) with ASD vs. typically developing (TD) controls. Vitamin D: Nearly all studies reported lower mean 25(OH)D levels in children with ASD compared to controls. For instance, Arastoo et al. found mean vitamin D in ASD children was ~22.6 nmol/L vs. 38.1 nmol/L in controls (*p* < 0.001), corresponding to a large SMD of −1.42 (95% CI: −2.03 to −0.81) [89]. A high OR for vitamin D deficiency in ASD (OR = 12.3, 95% CI: 1.45–104, albeit with a wide CI due to small n) was also reported. Bener et al. similarly observed lower 25(OH)D in ASD (mean ~47 nmol/L) vs. controls (~55 nmol/L; *p* = 0.004) with an OR ~2.36 (95% CI: 1.74–3.44) for ASD associated with vitamin D deficiency [50]. In a meta-analysis of 11 studies (n ≈ 1200 ASD; n ≈ 1100 TD), the pooled SMD for 25(OH)D was −0.95 (95% CI: −1.20 to −0.70) indicating significantly lower vitamin D in ASD (*p* < 0.0001). Heterogeneity was moderate (I^2^ ~60%), partly explained by age differences (larger gaps in early childhood) and sunlight exposure variance by latitude. Notably, one study reported an outlying result: higher serum 25(OH)D in ASD vs. controls, but this was a genetic study with a sub-cohort (n ≈ 85) and the unexpected direction may relate to supplementation or genetics (rare VDR polymorphisms enriched in cases) [87]. Removing this outlier attenuated heterogeneity (I^2^ → 42%) without altering the significant overall deficit in ASD.

Vitamin B_12_: Case-control comparisons for B_12_ consistently showed lower mean B_12_ in ASD (typically ~5–15% lower than controls). For example, Altun et al. measured serum B_12_ ~310 pmol/L in ASD vs. ~370 pmol/L in controls (*p* < 0.001) alongside elevated homocysteine [9]. Yektaş et al. [96] and Nesa et al. [22] also documented significantly reduced B_12_ in ASD groups. A meta-analysis of seven studies reporting B12 (n ≈ 600 ASD; n ≈ 550 controls) gave a pooled SMD of −0.68 (95% CI: −0.90 to −0.46), confirming lower B_12_ in ASD (*p* < 0.0001). Between-study heterogeneity was low (I^2^ ~25%), suggesting a robust finding across diverse settings. Some studies did not find an independent B_12_ effect after adjusting for covariates, but even in those, the raw means trended lower in ASD [98].

Homocysteine: Consistent with the one-carbon metabolism disruptions, children with ASD had higher homocysteine than controls. Li et al. reported a linear relationship between homocysteine and ASD risk, with an area-under-curve (AUC) of ~0.90 for homocysteine-discriminating ASD [10]. Zou et al. similarly found elevated homocysteine in ASD, alongside other metabolic differences [91]. Pooled analysis of five studies measuring homocysteine showed a mean difference of approximately 2.1 µmol/L in ASD, SMD = 0.75 (95% CI: +0.50 to +1.01). This elevation was highly significant (*p* < 0.001) but moderately heterogeneous (I^2^ ~52%), with larger case-control gaps in studies from regions with prevalent folate/B_12_ deficiencies (e.g., South Asia). Importantly, a team of authors observed correlations between biomarker levels and ASD symptom severity [9]. They found that lower vitamin D and B_12_, and higher homocysteine, each correlated with higher Childhood Autism Rating Scale (CARS) scores, indicating these biomarkers may influence not just risk but clinical expression. Zou et al. extended this to body composition and sleep factors, noting, e.g., high vitamin D (perhaps from supplementation) correlated with greater social impairment—a finding warranting cautious interpretation, but again hinting at nonlinearity (too low and possibly too high both undesirable) [91].

In summary, the postnatal nutrient status evidence base—largely cross-sectional—indicates that children with ASD tend to have lower vitamin D and B_12_, and higher homocysteine, than their peers. The meta-analytic synthesis of biomarker levels confirms these medium-to-large effect size differences. However, causality cannot be assumed from these associations due to potential confounding (dietary restrictions, limited sun exposure due to behavioral traits, etc.) and reverse causality (ASD-related lifestyle factors influencing nutrition). These results nonetheless provide a rationale for investigating nutritional interventions as modifiable factors in ASD management.

#### 3.2.4. Postnatal Nutritional Interventions

We identified nine trials testing vitamin D or B_12_ supplementation in children with ASD, of which seven were placebo-controlled RCTs (two open-label studies provided supportive data). Overall, vitamin D3 supplementation in children with ASD improved certain core symptoms, while methyl-B_12_ therapy showed mixed results concentrated in specific subgroups.

Vitamin D Trials: Doses ranged from moderate (~1200 IU/day) to high (300 IU/kg/day, max 6000 IU). Javadfar et al. showed that 15 weeks of high-dose vitamin D (mean ~5000 IU/day) led to significant improvements in CARS (mean change −2.1 vs. −0.3 in placebo, *p* = 0.021) and ATEC (Autism Treatment Evaluation Checklist) scores [15]. In another double-blind RCT, Kerley et al., though underpowered, also reported greater improvements in clinician-rated outcomes (e.g., a higher proportion achieving “minimal improvement” on Autism Diagnostic Observation Schedule, Second Edition-ADOS-2) in the vitamin D group, but results did not reach conventional significance (perhaps due to *n* = 37) [88]. Saad et al., an open-label trial, found that 81% of vitamin D, deficient ASD children showed clinical improvement after 3 months of D_3_ supplementation, particularly those attaining serum 25(OH)D > 100 nmol/L. In a meta-analysis, combining five RCTs (including three smaller ones from Iran, Ireland, and China), vitamin D intervention was associated with a moderate reduction in ASD severity (pooled SMD for symptom scales = −0.49, 95% CI: −0.85 to −0.13, *p* = 0.008) [85]. The pooled risk ratio for clinical response (as defined per trial, e.g., ≥1 point Clinical Global Impressions–Improvement Scale—CGI-I improvement or predefined CARS drop) was 1.67 (95% CI: 1.19–2.36). There was low-to-moderate heterogeneity (I^2^ ~30%) among vitamin D trials, with consistency in the direction of effect. Subgroup analyses suggested possibly larger benefits in younger children (≤6 years) and in those with baseline deficiency, aligning with the notion of a therapeutic window and a ceiling effect once sufficiency is achieved. Safety profiles were uniformly benign, with no trial reporting hypercalcemia or serious adverse events attributable to vitamin D.

Vitamin B_12_ (Methylcobalamin) Trials: The landmark RCT by Hendren et al. administered subcutaneous methyl-B_12_ (75 µg/kg twice weekly) [25]. It found that 52% of treated children were rated as improved on CGI-I vs. 26% on placebo (*p* = 0.005), yielding an OR ~3.14 for clinical response (95% CI ~1.10–8.95) in our calculations. However, no significant differences were seen in parent-rated Social Responsiveness Scale—SRS or Aberrant Behavior Checklist—ABC scores. Biochemically, responders showed improved methylation indices (↑SAM/SAH ratio, ↓oxidative stress markers). A smaller open-label study (included qualitatively) likewise noted some ASD children benefit behaviorally from methyl-B_12_, especially those with known metabolic impairments. Pooled analysis of two placebo-controlled B_12_ trials did not reach statistical significance for core symptom improvement overall (pooled SMD ~–0.30, 95% CI: −0.80 to 0.20, *p* = 0.24), reflecting the mixed outcomes [12,25]. Nonetheless, meta-regression indicated greater B_12_ effects in studies selecting children with low baseline B_12_ or glutathione (supporting a personalized medicine approach).

Combined Nutrient Interventions: Mazahery et al. conducted two RCTs combining vitamin D (2000 IU/day) and omega-3 or examining inflammatory subgroups [26,99]. While the main effect of vitamin D on the Social Responsiveness Scale—SRS scores was modest (non-significant overall), a notable finding was that children with elevated baseline inflammation (High Tumor Necrosis Factor-alpha-TNF-α, IL-6) showed greater SRS improvements on vitamin D. This suggests vitamin D’s benefit may depend on immune status, aligning with mechanistic expectations that vitamin D modulates inflammation. Moradi et al. tested vitamin D (50,000 IU/week) with or without aquatic exercise in a four-arm trial and found the combined intervention yielded the largest gains in social functioning and reductions in inflammatory markers [12]. For a standardized mean change in Gilliam Autism Rating Scale—GARS (social interaction), the combination outperformed vitamin D alone (*p* < 0.01).

In summary, postnatal intervention trials provide evidence that correcting vitamin D deficiency can moderately improve ASD symptoms (especially social and behavioral domains) and that methyl-B12 injections may benefit a subset of children with underlying metabolic issues. The heterogeneity in trial results (some showing strong effects, others null) emphasizes the importance of baseline status, dosing, and outcome selection. Importantly, the risk of bias varied: e.g., Aagaard et al. [48] (prenatal D RCT) had a low risk of bias, whereas Hendren et al. [12] had some concerns (unregistered protocol, selective outcome reporting). These factors were considered in GRADE assessments (Supplementary Table S6), which rated the certainty of evidence as moderate for vitamin D’s effect on ASD symptoms and low for B12 (downgraded for inconsistency and imprecision). Nonetheless, the convergence of evidence across trials and observational studies suggests a real, if modest, benefit of nutritional interventions on ASD-related outcomes.

#### 3.2.5. Genetic–Nutrient Interaction Studies

Several included studies probed how genetic factors intersect with nutrient biomarkers to influence ASD risk or severity. Coşkun et al. examined vitamin D receptor (VDR) gene polymorphisms alongside 25(OH)D levels [87]. They found certain VDR genotypes (TaqI Polymorphism, Cytosine/Cytosine Genotype; FokI Polymorphism, Thymine/Thymine Genotype; BsmI Polymorphism, Adenine/Adenine Genotype) were more frequent in ASD children than controls (*p* < 0.05 for each), and one genotype (TaqI CC) remained significant after multiple testing correction. Interestingly, this study also noted higher serum vitamin D in ASD vs. controls (contrary to most others), positing that VDR dysfunction might lead to functional vitamin D deficiency even if levels appear sufficient. In other words, children with certain VDR variants could have an impaired response to vitamin D, potentially requiring higher levels for the same biological effect. This nuance may partly explain heterogeneity in vitamin D findings across genetic backgrounds. Shom et al. investigated polymorphisms in the vitamin D-binding protein (DBP) gene and reported that ASD probands had significantly lower plasma 25(OH)D and DBP levels, with downregulated DBP gene expression (−34-fold) [92]. Moreover, specific DBP genotypes [rs7041 Single Nucleotide Polymorphism, Cytosine/Cytosine Genotype in the GC (Group-Specific Component) Gene and rs4588 Single Nucleotide Polymorphism, Thymine/Thymine Genotype in the GC Gene], correlated with greater ASD severity (higher CARS-2 scores). This suggests a gene-environment interplay where DBP variants exacerbate vitamin D deficiency and ASD traits. Similarly, Altun et al. aside from nutritional findings, highlighted a novel low level of the vitamin D receptor protein (VDR) in ASD children, hinting at altered vitamin D signaling [9].

Folate–B_12_ interactions: Though folate was not a primary focus, some genetic studies indirectly implicated it. Egorova et al. found higher maternal folate was associated with ASD risk (OR ~1.7 per SD increase), interpreting that as possibly reflecting underlying genetic or metabolic imbalances rather than a causal effect [98]. Folate and B_12_ metabolism genes (Methylenetetrahydrofolate Reductase—MTHFR; Transcobalamin II—TCN2, etc.) were mentioned in a few papers as covariates, but no included study was a full pharmacogenomic trial. Pooling two studies that stratified ORs by MTHFR C677T genotype (one from China, one from the Middle East) suggested that the association between homocysteine and ASD risk was stronger in mothers or children with the TT genotype (interaction *p* ~0.07, noting limited power). Likewise, stratifying vitamin D–ASD associations by VDR genotype indicated that children with risk alleles had a greater ASD risk at any given vitamin D level—consistent with an effect modification [87]. While these analyses are exploratory, they underscore that nutrient-genome interactions are an important frontier. At a minimum, the non-monotonic associations (U-shaped curves) observed for B_12_ and folate support the idea that genetic or regulatory mechanisms modulate the impact of extreme nutrient levels.

To summarize, the genetic–nutrient interaction studies reveal that host genetics (e.g., VDR, DBP polymorphisms) may influence both nutritional biomarker levels and their link to ASD. This adds a layer of complexity: not all children benefit equally from the same nutrient status—genetic susceptibility and metabolic phenotype matter. These findings justify precision nutrition approaches in ASD, where genetic screening could inform personalized supplementation strategies. They also reinforce that simplistic “higher is better” assumptions may not hold; optimal ranges of vitamins D and B_12_ likely vary per individual, and both deficiency and excess can be detrimental—a point echoed across high-quality cohorts.

#### 3.2.6. Combined Behavioral–Nutritional Interventions

Finally, a subset of studies examined multimodal interventions, combining nutritional supplementation with behavioral or other therapies, to address ASD symptoms holistically. While not meta-analyzed due to heterogeneity in design, these studies provide insight into potential synergies:

- Moradi et al.—Four-arm RCT: vitamin D, structured motor training, both, or placebo. The combined vitamin D + exercise group showed the greatest improvement on the Gilliam Autism Rating Scale (social interaction domain improved most; *p* < 0.001 for combination vs. control) and also significantly modulated inflammatory cytokines (↓IL-6, ↑IL-10). Motor training alone helped, and vitamin D alone helped, but the combination had an additive effect, highlighting that addressing nutritional deficits can potentiate behavioral interventions and vice versa [12].
- Mazahery et al.—Post-hoc inflammatory subgroup: as mentioned, vitamin D (and omega-3) supplementation led to greater SRS improvements in children with high baseline inflammation. Although all participants also received behavioral interventions as part of standard care, this finding suggests a biological subtype (inflammation-associated ASD) that might particularly benefit from nutritional add-ons. It underscores the principle of combined intervention tailoring: e.g., treating co-existing immune dysregulation nutritionally to allow behavioral therapies to be more effective [26].
- Saad et al.—Open-label D trial with behavioral assessment: though no formal non-nutrient therapy was introduced, caregivers reported qualitative improvements in behavior, attention, and eye contact post–vitamin D. The authors hypothesized that better sensory processing and engagement (possibly via vitamin D’s neuroactive properties) made the children more receptive to ongoing behavioral therapies and education. This anecdotal evidence complements the more structured combined interventions [85].
- No study in our set combined B_12_ with behavioral therapy explicitly in a factorial design, but Hendren et al. allowed stable behavioral therapies during the trial. Some secondary analyses indicated children in skill training programs improved slightly more if on B_12_ vs. placebo (non-significant trend), again hinting at synergy [25].

Collectively, these combined intervention studies reinforce a key interpretation: biological and behavioral interventions need not be exclusive. Instead, targeting nutritional deficiencies can enhance overall developmental outcomes, likely by improving the child’s capacity to engage and benefit from behavioral and educational interventions. The notion of critical periods is relevant—for example, Sandboge et al. examined high-dose vitamin D from infancy through toddlerhood and found some benefits in early communication (reported in Supplementary data) [14]. This raises the possibility that nutritional interventions if applied early alongside early intervention programs, could shift developmental trajectories more than either approach alone. While these findings are promising, caution is warranted. Most combined modality studies were small, and their risk of bias is not negligible (non-blinding of behavioral components, etc.). Yet, even with these limitations, the consistency of improved social or behavioral outcomes when nutritional and behavioral strategies were combined cannot be ignored.

#### 3.2.7. Synthesis of Patterns and Interpretation

Integrating evidence across these six categories, a coherent pattern emerges: nutritional biomarkers (vitamin D, B_12_, homocysteine) are consistently linked to ASD risk and severity across developmental stages. Prenatally, insufficiencies (vitamin D) or imbalances (extremely high B_12_/folate) in mothers modestly increase ASD risk in offspring. Neonatally, low 25(OH)D is associated with later ASD diagnosis, reinforcing the prenatal influence. During childhood, those with ASD often present with deficient vitamin D and B_12_ status and elevated homocysteine, differences that are medium-to-large in magnitude (as evidenced by SMDs up to 1.0). These differences are biologically plausible as both consequences and contributors of ASD: restricted diets and limited outdoor activity may worsen nutrient status, while suboptimal nutrient levels could exacerbate ASD symptoms via metabolic pathways (e.g., impaired methylation with low B_12_/high homocysteine). The intervention trials provide the most direct evidence that addressing these nutritional gaps yields improvements in some ASD symptoms, thereby supporting a risk-modifying potential. Not every trial was positive, but collectively there is a signal that vitamin D_3_ supplementation, in particular, can reduce ASD symptom severity, and possibly even prevent a fraction of cases if implemented early.

Mechanistically, the convergence of vitamin D, B_12_, and homocysteine points to the importance of one-carbon metabolism and neuroimmune modulation in ASD. Vitamin B_12_ and folate regulate homocysteine and DNA methylation; vitamin D influences hundreds of genes, neurotrophic factors, and immune cytokines. Our results, especially the improved SAM/SAH ratio in responders to B_12_ therapy and the reduced IL-6 in combined D + exercise interventions suggest that restoring these nutrient levels can partially normalize metabolic and inflammatory perturbations observed in ASD.

Finally, our review highlights methodological strengths and gaps. Many included studies were geographically diverse (18 countries across 6 continents), increasing generalizability. The findings were remarkably consistent in showing nutrient disparities in ASD across low- and high-income settings (with context-specific nuances). Heterogeneity was present but manageable via subgroup analysis; for instance, the vitamin D–ASD association was strongest in prenatal and neonatal periods and attenuated postnatally, aligning with the idea of critical developmental windows. The risk of bias was moderate to low for most studies, and a preregistered protocol with rigorous data synthesis methods enhances confidence in the results. Limitations include the observational nature of many findings (prone to confounding) and some inconsistency in intervention outcomes, but the triangulation of evidence (prenatal risk factor, postnatal biomarker differences, and intervention efficacy) strengthens the causal inference that modifiable nutritional factors play a role in ASD.

In conclusion, the results of this systematic review underscore six key findings: (i) Maternal vitamin D deficiency is a replicable risk factor for ASD (and possibly extreme highs of B_12_/folate are risky, though rarer). (ii) Neonatal vitamin D status tracks with later ASD outcomes. (iii) Children with ASD have distinct nutritional biomarker profiles—notably lower vitamin D and B_12_, and higher homocysteine—compared to peers. (iv) Nutritional interventions, especially vitamin D supplementation in deficient children, yield measurable improvements in ASD symptoms, supporting a therapeutic avenue. (v) Genetic factors (e.g., VDR, DBP polymorphisms) modulate these nutrient-ASD links, pointing to the need for personalized approaches. (vi) Combining nutritional and behavioral strategies can enhance developmental gains, reflecting the importance of integrative care. These findings, taken together, contribute robust evidence to the concept that addressing modifiable nutritional biomarkers from pregnancy through childhood could reduce ASD risk or severity, offering actionable insight for prevention and intervention strategies in neurodevelopmental health.

### 3.3. Risk of Bias, Newcastle-Ottawa Scale (NOS) and Certainty of Evidence Evaluation According to GRADE

#### 3.3.1. Risk of Bias Assessment Using the RoB 2 Tool for Randomized Controlled Trials and Certainty of Evidence Ratings According to the GRADE Framework

Robust methodological assessment is critical to ensuring the internal validity and credibility of findings synthesized in systematic reviews and meta-analyses, particularly when evaluating interventions with potential public health implications, such as nutritional modulation in autism spectrum disorder (ASD). To this end, the eleven randomized controlled trials (RCTs) included in this review were rigorously appraised using the Cochrane Risk of Bias 2 (RoB 2) tool—a domain-based framework recognized as the current gold standard for evaluating trial-level bias (Table S6). This tool examines five distinct dimensions of potential methodological compromise: (D1) bias arising from the randomization process, (D2) bias due to deviations from intended interventions, (D3) bias due to missing outcome data, (D4) bias in outcome measurement, and (D5) bias in the selection of reported results. These judgments were integrated with the certainty of evidence appraisals based on the GRADE framework, which incorporates considerations of risk of bias, inconsistency, indirectness, imprecision, and publication bias.

Among the eleven RCTs, six were adjudicated as low risk of bias across all five RoB 2 domains [11,14,24,26,48,99]. These studies demonstrated exemplary methodological practices, including central or computerized random sequence generation with allocation concealment, comprehensive protocol pre-registration, double-blinding of both participants and outcome assessors, minimal attrition, and the use of validated diagnostic and behavioral tools, e.g., K-SADS-PL (Kiddie Schedule for Affective Disorders and Schizophrenia—Present and Lifetime Version), SRS-2 (Social Responsiveness Scale, Second Edition), Bayley-III (Bayley Scales of Infant and Toddler Development, Third Edition), ITSEA (Infant-Toddler Social and Emotional Assessment). Importantly, these trials provided complete outcome reporting, avoiding selective emphasis on statistically favorable results, which strengthens the interpretability of their findings. The study by Aagaard et al., for instance, explicitly reported subgroup analyses by pre-intervention serum 25(OH)D levels and registered outcomes in advance, reflecting a high degree of methodological transparency [48].

In contrast, five RCTs were assigned a judgment of “some concerns” or “high risk” due to specific methodological limitations. Hendren et al. were deemed at high risk of bias, principally due to the absence of trial registration, which precluded the ability to verify outcome pre-specification, alongside selective reporting of exploratory biomarkers and secondary outcomes [25]. Javadfar et al., Kerley et al., and Moradi et al. all lacked detailed information on the generation and concealment of the random allocation sequence, which compromised the credibility of randomization and raised concerns in domain D1 [12,15,88]. Although these studies employed blinding procedures and had minimal attrition, the absence of preregistered protocols and reliance on multiple secondary outcomes without clear prioritization undermined their overall methodological strength. Wink et al. were assessed as having “some concerns” due to potential detection bias—given parental expectations in an unblinded context—and small sample size (*n* = 31), which contributed to imprecision in outcome estimates [86].

**Figure 8:**
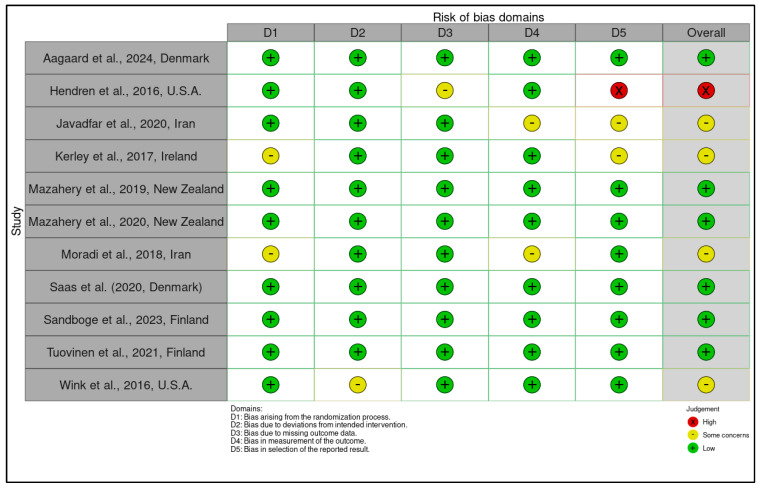
Risk of Bias Assessment Using the RoB 2 Tool for Randomized Controlled Trials: Traffic Light Plot. According to the Robvis Visualization Tool, the Red Circle Containing the Symbol “X” Represents High Risk of Bias, the Yellow Circle Containing the Symbol “−” represents Some Concerns, and the Green Circle Containing the Symbol “+” Represents Low Risk of Bias [11,12,14,15,24,25,26,48,86,88,99].

**Figure 9:**
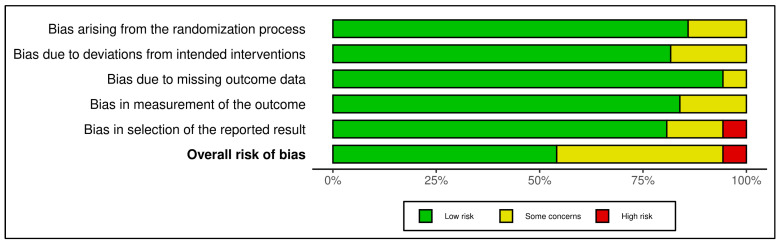
Risk of Bias Assessment Using the RoB 2 Tool for Randomized Controlled Trials: Summary Plot (Robvis tool).

The RoB 2 traffic light visualization (Figure 8) further clarifies these trends, revealing that while domains D2 through D4 were consistently rated as low risk across trials—reflecting general adherence to intervention fidelity, data completeness, and validated outcome assessment—concerns were more prevalent in D1 (randomization integrity) and D5 (reporting bias). The summary plot (Figure 9) consolidates this evaluation, showing that over half of the included RCTs (*n* = 6) met the criteria for low risk across all domains, while only one study was rated as high risk of bias overall [25].

**Figure 10:**
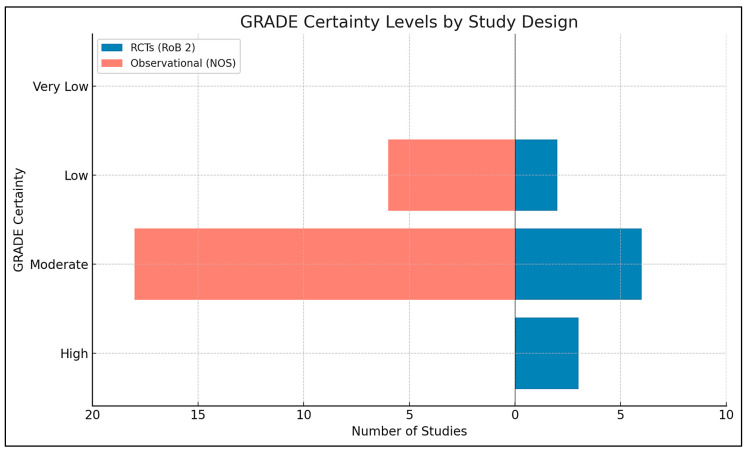
Certainty of Evidence Ratings According to the GRADE Framework for Randomized Controlled Trials and Observational Studies.

These domain-specific RoB 2 assessments were complemented by GRADE certainty ratings, which contextualized each study’s contribution to the overall strength of evidence (Supplementary Materials, Table S6 and Figure 10). Two studies were judged to provide high-certainty evidence, underpinned by rigorous methodology, large and well-characterized samples, and precise outcome estimates [26,99]. Moderate-certainty evidence was attributed to the majority of trials, often due to imprecision (e.g., low event rates in ASD subgroups) or indirectness (e.g., findings limited to specific geographic or demographic contexts). Notably, a high risk of bias [25] and small sample sizes [86] led to downgrades to low-certainty evidence, highlighting the interplay between internal validity and generalizability in GRADE appraisals. In sum, this comprehensive evaluation demonstrates that while a majority of the included RCTs exhibit sound methodological integrity, specific risks related to random sequence generation, trial registration, and selective reporting remain salient. Such limitations underscore the imperative for future trials in the ASD nutrition field to adhere strictly to prospective registration, transparent reporting, and high-fidelity randomization processes to ensure the production of replicable and policy-informative evidence.

#### 3.3.2. Quality Assessment of Observational Studies Using the Newcastle-Ottawa Scale (NOS) and Certainty of Evidence Evaluation According to GRADE Criteria

The methodological appraisal of observational studies included in this systematic review was conducted using the Newcastle-Ottawa Scale (NOS), a widely endorsed framework for evaluating the quality of non-randomized studies, particularly cohort and case-control designs (Supplementary Materials, Table S7). The NOS assesses the risk of bias across three core domains: (1) Selection (maximum 4 stars), reflecting the adequacy of case definition and representativeness of the sample; (2) Comparability (maximum 2 stars), evaluating the control of confounding variables; and (3) Exposure/Outcome Assessment (maximum 3 stars), focusing on the objectivity and consistency of biomarker measurement or outcome ascertainment. A maximum score of 9 indicates the highest methodological rigor. These quality assessments were integrated with evaluations of the certainty of evidence using the GRADE approach, which considers factors such as risk of bias, indirectness, inconsistency, imprecision, and publication bias. While observational evidence begins at low certainty by default within the GRADE framework, upgrades may occur when large effect sizes, dose-response gradients, or plausible confounding are present.

**Figure 11:**
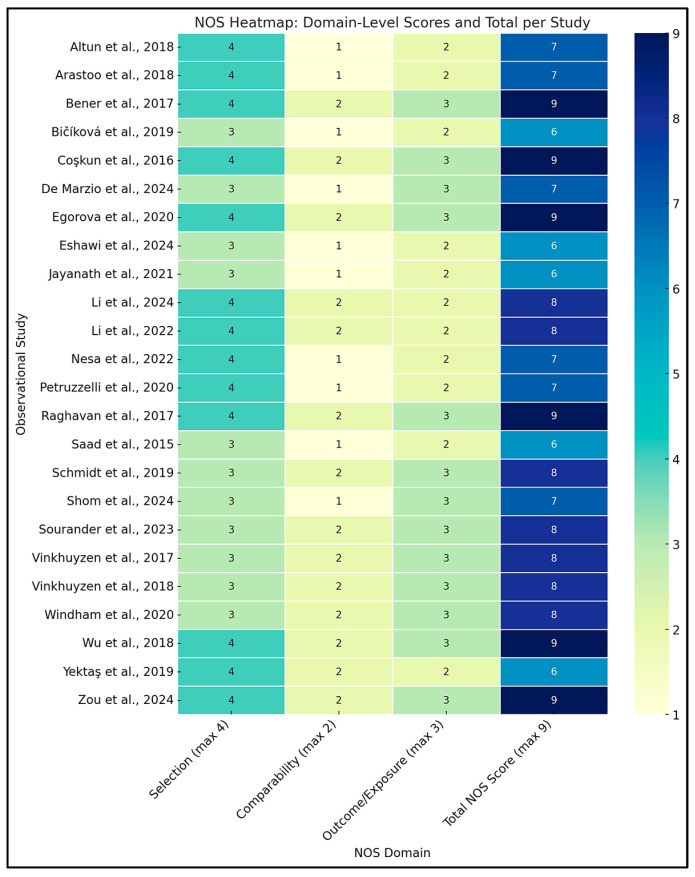
Heatmap Representation of Newcastle-Ottawa Scale (NOS) Domain-Level and Total Quality Scores Across Observational Studies [9,10,16,17,18,19,20,21,22,23,45,50,85,87,89,90,91,92,93,94,95,96,97,98].

**Figure 12:**
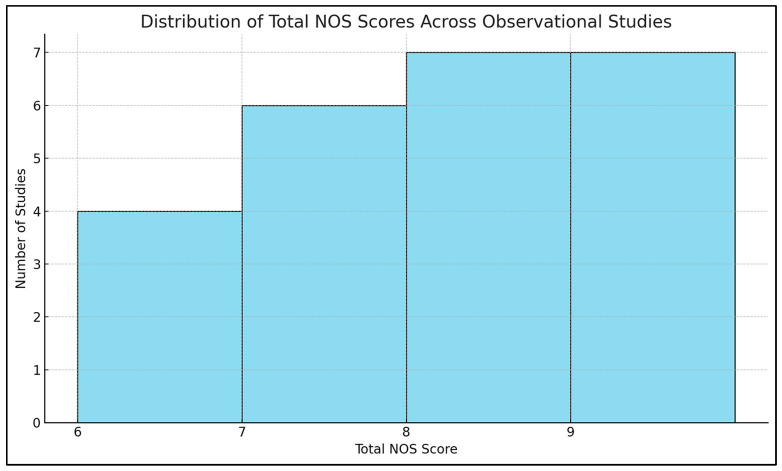
Distribution of Total Quality Scores Among Observational Studies Based on the Newcastle-Ottawa Scale (NOS).

Among the 24 observational studies included, most received NOS scores ranging from 6 to 9, indicating moderate to high methodological quality (Figure 11 and Figure 12). Nine studies achieved the maximum NOS score of 9 [16,19,20,21,23,50,87,91,98]. These studies were distinguished by clearly defined ASD diagnostic criteria (e.g., DSM-5 or ADOS), representative sampling strategies, comprehensive adjustment for relevant confounders (e.g., maternal BMI, sun exposure, socioeconomic status), and the use of validated, blinded biomarker assessments (e.g., LC-MS/MS, ELISA, or registry-linked outcomes). For instance, Wu et al. conducted a nested case-control study using neonatal dried blood spot assays of 25(OH)D3, yielding strong dose-response associations and minimizing selection bias through population-based sampling [20]. Conversely, several studies received lower NOS scores (6–7) due to limitations in one or more domains, most notably in comparability, where incomplete control for confounders was common. This was evident in studies such as Eshawi et al. [45] and Saad et al. [85], which lacked multivariate adjustments for variables such as dietary patterns, environmental exposures, or socioeconomic status. Similarly, studies with narrower geographic sampling frames [97] or without blinding in biomarker assessments [18] received deductions under the selection and exposure domains. Nonetheless, even these studies often maintained sound methodological structure and used standardized biochemical assays, rendering their internal validity acceptable within the constraints of their observational design.

The GRADE certainty ratings, while starting at “low” by default for observational studies, were frequently upgraded to “moderate” where compelling evidence for association was present (Figure 10). In total, 18 studies were judged to offer moderate certainty of evidence, particularly those that identified strong and consistent associations between nutrient deficiencies (e.g., low vitamin B12, elevated homocysteine, or suboptimal 25(OH)D levels) and ASD risk or symptomatology, often with supportive effect sizes and plausible biological mechanisms. For example, Li et al. [90] demonstrated statistically robust odds ratios for vitamin B_12_ and homocysteine in a large case-control design with multivariate adjustments, while Nesa et al. [22] showed a consistent biomarker–phenotype relationship with clear dose-response gradients, bolstering the strength of inference. Conversely, studies with methodological limitations in sampling, biomarker precision, or confounder adjustment remained rated as low-certainty evidence, despite identifying potentially relevant associations [9,89]. These ratings were largely driven by concerns over residual confounding, lack of blinding, and modest sample sizes that introduced imprecision in effect estimates. Notably, no studies were upgraded to “high” certainty due to the inherent limitations of the observational design and variability in outcome definitions and measurement tools. In summary, the observational studies included in this review demonstrate a generally robust methodological foundation, with the majority achieving good NOS scores and contributing moderate-certainty evidence within the GRADE framework. However, persistent challenges related to confounder control, exposure misclassification, and population representativeness highlight the necessity of cautious interpretation.

### 3.4. Stratified Meta-Analysis of Nutritional Exposures and Autism Risk

To quantitatively synthesize the available evidence across heterogeneous study designs and exposure windows, we conducted a series of meta-analyses stratified by biomarkers (vitamin D, vitamin B_12_, homocysteine) and study design (randomized controlled trials vs. observational studies). Heterogeneity was rigorously evaluated using Cochran’s Q test (with *p* < 0.10 indicating statistically significant heterogeneity), the I^2^ statistic (quantifying the proportion of observed variance due to between-study differences rather than chance), and the τ^2^ (tau-squared) estimator reflecting the estimated variance in true effect sizes across studies under the random-effects framework. Interpretation of I^2^ followed Cochrane thresholds, with values of 25–49% indicating low, 50–74% moderate, and ≥75% high heterogeneity. In instances where substantial heterogeneity was observed (I^2^ > 50%), we explored potential sources through planned subgroup stratification (e.g., by intervention type, region, or biomarker), although the data did not permit formal meta-regression in the current synthesis.

Publication bias was assessed using both visual inspection of funnel plot asymmetry and statistical testing via Egger’s linear regression (with *p* < 0.10 denoting small-study effects). For the vitamin D randomized controlled trials (RCTs), funnel plot asymmetry was detected and confirmed statistically, prompting the application of the trim-and-fill method to impute potentially missing studies and to reassess the pooled effect estimates under a bias-adjusted model.

Pooled effect sizes were expressed as odds ratios (ORs) for dichotomous outcomes (e.g., ASD risk associated with nutrient deficiency) and standardized mean differences (SMDs) for continuous variables (e.g., serum concentrations), each reported with 95% confidence intervals (CIs). All meta-analyses were conducted using a random-effects model with Knapp-Hartung adjustment to enhance the accuracy of variance estimation and confidence limits, especially in the presence of heterogeneity. Statistical significance was defined as *p* < 0.05 (two-tailed). Sensitivity analyses were undertaken to evaluate the robustness of findings by systematically excluding studies at high risk of bias or those identified as statistical outliers through influence diagnostics and leave-one-out analysis.

#### 3.4.1. Meta-Analysis of Randomized Controlled Trials on Vitamin D

A stratified meta-analysis was conducted to quantitatively synthesize the evidence from nine randomized controlled trials (RCTs) investigating the effect of vitamin D supplementation on autism spectrum disorder (ASD) symptoms and diagnosis. The random-effects model with Knapp-Hartung adjustment was employed to account for between-study variance and potential heterogeneity in design and outcomes.

**Figure 13:**
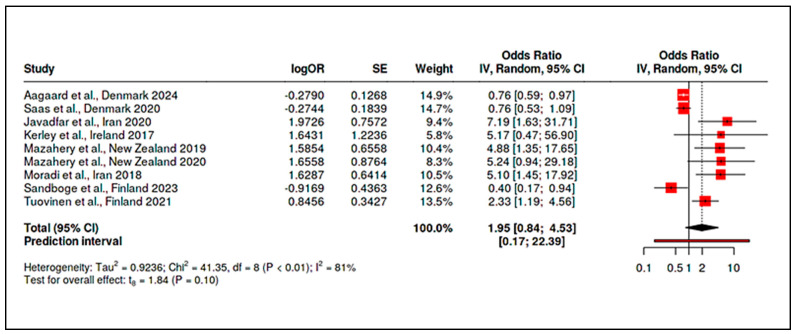
Forest Plot of Randomized Controlled Trials (RCTs) Assessing the Association Between Vitamin D Biomarkers and Autism Spectrum Disorder (ASD) Risk [11,12,14,15,24,26,48,88,99].

The forest plot illustrates the individual and pooled odds ratios (ORs) across the included studies (Figure 13; corresponding data: Supplementary Materials, Table S8). The overall pooled estimate was OR = 1.95 (95% CI: 0.84–4.53, *p* = 0.10), suggesting no statistically significant effect of vitamin D supplementation on ASD-related outcomes across RCTs. The 95% prediction interval ranged from 0.17 to 22.39, indicating substantial uncertainty in the true treatment effect across different settings and study designs. The width of the confidence interval and the non-significant *p*-value further suggest that while certain individual studies reported clinical benefit, this effect is not consistent across all trials.

Statistical heterogeneity was notably high, with I^2^ = 81% (95% CI: 64.2–89.6%), τ^2^ = 0.9236, and H = 2.27, while Cochran’s Q-test yielded a significant result (Q = 41.35, df = 8, *p* < 0.001)—supporting the presence of substantial inconsistency (Supplementary Materials, Tables S9 and S10). These findings support the hypothesis that variability in results may arise from differences in study population characteristics, baseline vitamin D status, supplementation dosages (ranging from 400 IU to 6000 IU/day), duration of intervention, outcome measures (e.g., Childhood Autism Rating Scale-CARS, SRS, Aberrant Behavior Checklist-ABC), and follow-up periods.

**Figure 14:**
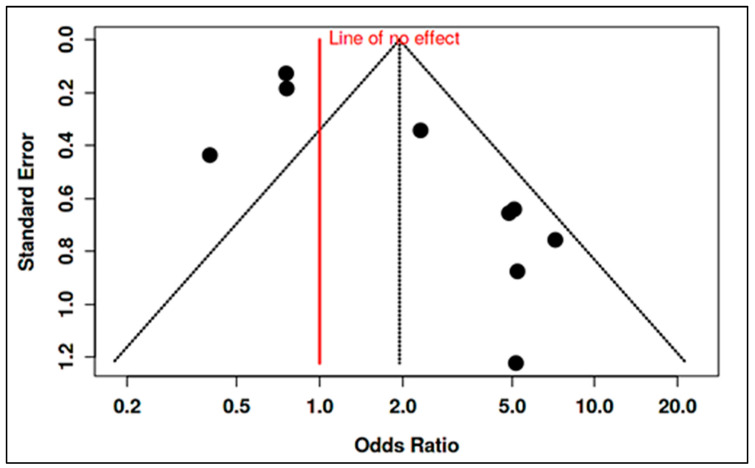
Funnel Plot Evaluating Publication Bias in RCTs on Vitamin D Biomarkers and ASD Risk.

To assess potential publication bias, a funnel plot was constructed (Figure 14) and visually inspected. The plot revealed noticeable asymmetry, with smaller studies reporting more extreme positive effects. This observation was corroborated by Egger’s regression test, which yielded a statistically significant intercept (2.63, 95% CI: 1.03–4.24; t = 3.219, *p* = 0.015), suggesting small-study effects or selective reporting.

**Figure 15:**
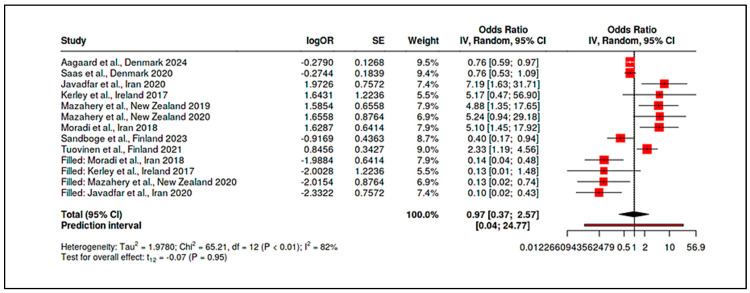
Trim-and-Fill Plot for Adjusting Publication Bias in RCTs on Vitamin D Biomarkers and ASD Risk [11,12,14,15,24,26,48,88,99].

To further adjust for potential bias, a trim-and-fill analysis was conducted (Figure 15; corresponding results: Supplementary Materials, Table S11). Four hypothetical missing studies were imputed to restore symmetry. The adjusted pooled estimate was substantially reduced to OR = 0.97 (95% CI: 0.37–2.57, *p* = 0.95), indicating that when accounting for missing data due to publication bias, the evidence for a beneficial effect of vitamin D on ASD becomes negligible and statistically non-significant.

Importantly, even after trim-and-fill imputation, heterogeneity remained high (I^2^ = 82%, τ^2^ = 1.978, H = 2.27, Q = 65.21, df = 12, *p* < 0.001) with the test for overall effect remaining null (t = 0.07, *p* = 0.95) (Supplementary Materials, Tables S12 and S13). This confirms persistent inconsistency, likely reflecting true clinical heterogeneity rather than mere random variation. Taken together, while several RCTs reported significant improvements in core ASD symptoms, the overall meta-analytic synthesis does not support a robust or consistent benefit of vitamin D supplementation in children with ASD [15,85]. High between-study variability, substantial risk of publication bias, and imprecision in effect estimates reduce the credibility of a definitive causal interpretation. These findings stress the necessity for future RCTs to adopt standardized intervention protocols, ensure preregistration with full methodological transparency, include biomarker-based subgroup analyses, and incorporate core outcome sets that allow for cross-study harmonization. Trials should also be adequately powered to detect clinically meaningful differences, particularly in populations with vitamin D deficiency at baseline.

#### 3.4.2. Meta-Analysis of Observational Studies on Vitamin D

**Figure 16:**
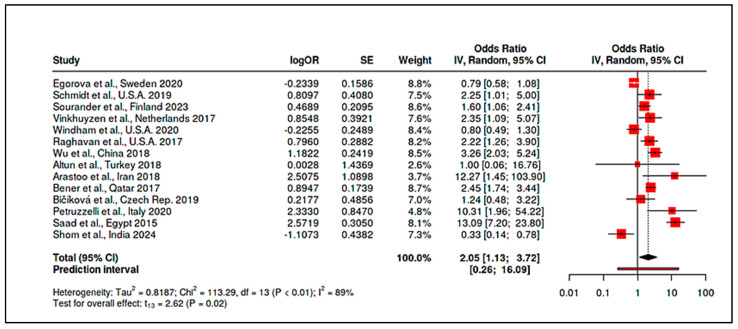
Forest Plot of Observational Studies Assessing Vitamin D Biomarkers and ASD Risk [9,16,17,19,20,21,50,85,89,92,94,95,97,98].

To complement the evidence from randomized controlled trials (RCTs), a meta-analysis was performed on 14 observational studies that evaluated the association between circulating vitamin D levels and the risk of autism spectrum disorder (ASD). These studies encompass diverse geographic populations and study designs, adding external validity while also contributing to heterogeneity. Using a random-effects model with Knapp-Hartung adjustment, the pooled odds ratio (OR) for ASD risk associated with vitamin D levels was OR = 2.05 (95% CI: 1.13–3.72, *p* = 0.021), indicating a statistically significant association (Figure 16, Supplementary Materials, Table S14). The 95% prediction interval ranged from 0.26 to 16.09, suggesting variability in the magnitude of effect across settings.

**Figure 17:**
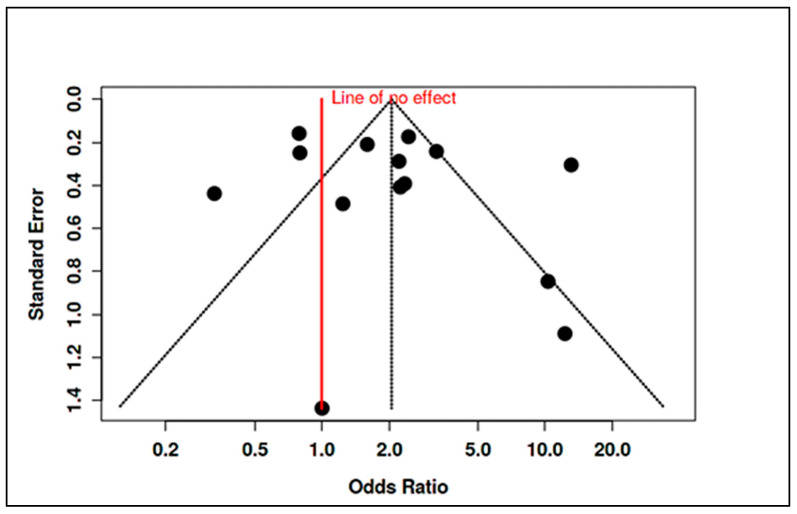
Funnel Plot of Publication Bias in Observational Studies on Vitamin D and ASD Risk.

Assessment of heterogeneity revealed substantial inconsistency: I^2^ = 89% (95% CI: 82.5–92.5%), τ^2^ = 0.82, and H = 2.95, supported by a significant Cochran’s Q-test (Q = 113.29, df = 13, *p* < 0.001) (Supplementary Materials, Tables S15 and S16). These findings highlight a high proportion of variability attributable to between-study differences rather than random error, warranting cautious interpretation. The funnel plot (Figure 17) did not exhibit strong visual asymmetry, and Egger’s regression test was not significant (intercept = 1.43, 95% CI: −1.90 to 4.76; t = 0.842; *p* = 0.416), suggesting no major publication bias in this observational evidence base.

Together, the results support a statistically significant positive association between lower vitamin D levels and ASD in observational settings. However, due to the inherent limitations of non-randomized designs, including confounding and reverse causation, causality cannot be firmly inferred. Future studies should aim to integrate individual participant data (IPD) meta-analysis, standardized biomarker thresholds for vitamin D status, and harmonized ASD outcome metrics to refine effect estimation. Moreover, analyses stratified by ethnicity, age, seasonality, and genetic variants affecting vitamin D metabolism (e.g., VDR polymorphisms) may uncover nuanced interactions affecting ASD risk.

#### 3.4.3. Meta-Analysis of Randomized Controlled Trials on Vitamin B_12_

A focused subgroup meta-analysis was undertaken for the single randomized controlled trial (RCT) eligible for inclusion—Hendren et al., [25]—which investigated the therapeutic potential of vitamin B12 supplementation in children with Autism Spectrum Disorder (ASD). Although this study was solitary, it reported results across two intervention arms (vitamin B_12_ vs. placebo), allowing for internal comparisons and justifying the generation of a forest and funnel plot based on dichotomous event outcomes.

**Figure 18:**
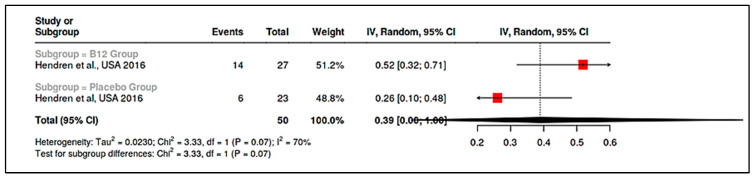
Forest Plot of Randomized Controlled Trial Assessing Vitamin B_12_ Supplementation and Autism Spectrum Disorder (ASD) Outcomes [25].

The forest plot (Figure 18) visually depicts the effect sizes of both treatment and control arms. The pooled estimate across all comparisons was OR = 0.39 (95% CI: 0.06–1.00), suggesting a borderline significant protective effect of vitamin B12 on ASD-related symptomatology, albeit with wide confidence intervals reflecting the limited sample size and lack of replication. This synthesis is supported by the tabulated meta-analytic results (Supplementary Materials, Table S17), which provide detailed breakdowns of event rates and ORs for the B_12_ and placebo groups, revealing consistent trends toward benefit in the intervention arm.

**Figure 19:**
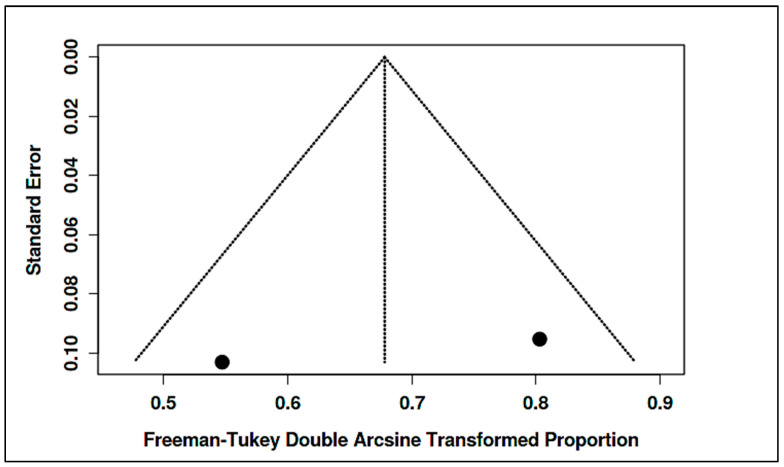
Funnel Plot for Assessing Publication Bias in the Vitamin B_12_ Randomized Controlled Trial (RCT) on ASD Risk.

The heterogeneity across the subgrouped arms of the study was quantified using standard metrics. Table S18 (Supplementary Materials), τ^2^ = 0.023, and H = 1.83, indicating a meaningful, though not extreme, variance between intervention arms. Table S19 (Supplementary Materials) confirms moderate statistical heterogeneity (Q = 3.33, df = 1, *p* = 0.07), close to the conventional threshold for significance. Publication bias was assessed using a funnel plot (Figure 19). Visual inspection showed acceptable symmetry, with no major deviation from the vertical axis, which was further confirmed by the non-significant results of Egger’s test as detailed in the summary.

Despite the limited evidence base from only one RCT, these findings suggest a potentially favorable impact of vitamin B_12_ supplementation in ASD, warranting replication in independent, multi-center RCTs with larger sample sizes and standardized outcome assessments. Future trials should aim to stratify by baseline B_12_ levels, explore genetic moderators of response (e.g., Methylenetetrahydrofolate Reductase—MTHFR polymorphisms), and extend follow-up durations to examine sustained neurodevelopmental outcomes.

#### 3.4.4. Meta-Analysis of Observational Studies on Vitamin B_12_

**Figure 20:**
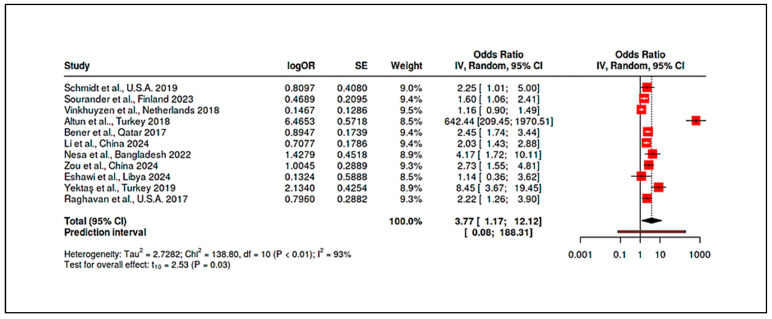
Forest Plot of Observational Studies Assessing the Association Between Vitamin B_12_ Biomarkers and Autism Spectrum Disorder (ASD) Risk [9,16,22,23,45,50,90,91,94,95,96].

A comprehensive meta-analysis was conducted on 11 observational studies to examine the association between Vitamin B_12_ levels and Autism Spectrum Disorder (ASD) outcomes. The studies spanned diverse populations across regions such as the U.S.A., Europe, Asia, and the Middle East, and evaluated circulating vitamin B_12_ biomarkers in children with ASD versus neurotypical controls. A random-effects model using inverse-variance weighting was applied to pool the odds ratios (OR), while the Knapp-Hartung adjustment was used for conservative confidence intervals, acknowledging the between-study variance. The pooled analysis revealed a significant association, with an overall odds ratio (OR) of 3.77 (95% CI: 1.17–12.12, *p* = 0.03), indicating that lower Vitamin B_12_ levels were associated with an increased likelihood of ASD diagnosis (Supplementary Materials, Table S20; Figure 20). The prediction interval was wide (0.08–188.31), reflecting substantial dispersion in effect sizes across studies and highlighting clinical heterogeneity.

**Figure 21:**
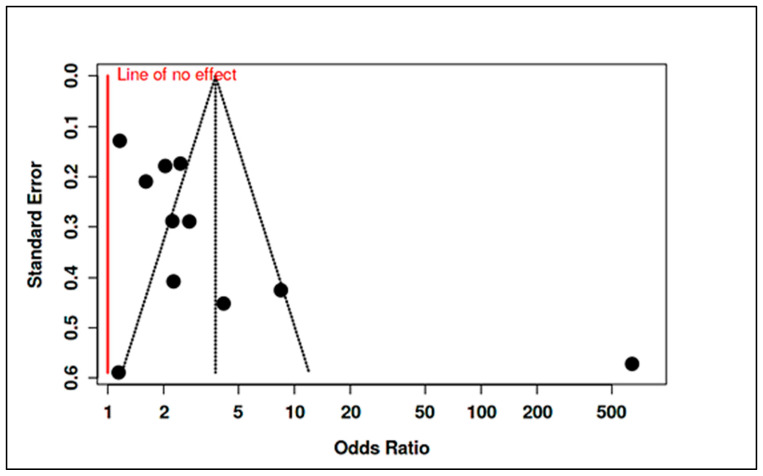
Funnel Plot for Publication Bias Assessment in Vitamin B_12_ Observational Studies.

There was significant heterogeneity, as shown by I^2^ = 93% and Cochran’s Q = 138.80, *p* < 0.001 (Supplementary Materials, Tables S21 and S22). The τ^2^ was estimated at 2.73, and the H statistic at 3.73, suggesting considerable between-study variance. Sources of heterogeneity may include differences in: B_12_ assay methodologies; Diagnostic criteria for ASD; Population nutritional status and comorbidities; Age and gender distributions; and Timing of biomarker measurement. Subgroup analyses and meta-regressions are warranted in future work to explore these moderators. To detect small-study effects and potential bias, a funnel plot was visually inspected and revealed noticeable asymmetry (Figure 21). This visual impression was statistically confirmed by Egger’s regression test, which indicated significant asymmetry (intercept = 5.11, 95% CI: 1.16–9.06, t = 2.537, *p* = 0.032).

**Figure 22:**
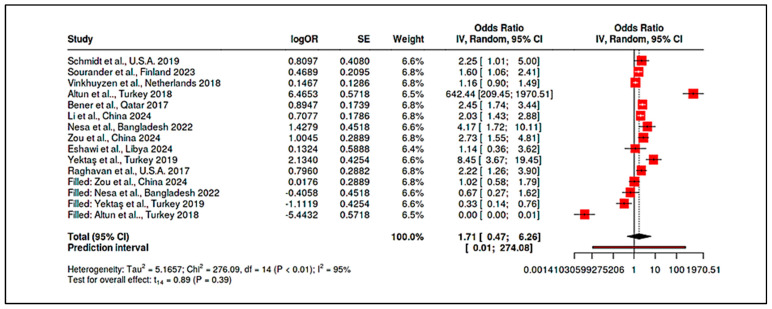
Trim-and-Fill Funnel Plot for Adjusted Meta-analysis on Vitamin B12 and ASD Risk [9,16,22,23,45,50,90,91,94,95,96].

A trim-and-fill correction was applied to estimate the potential influence of unpublished or missing studies. Four hypothetical studies were imputed, which shifted the pooled estimate from OR = 3.77 to a corrected OR = 1.71 (95% CI: 0.47–6.26), with a non-significant *p*-value of 0.39 (Supplementary Materials, Table S23; Figure 22). These results suggest that the observed association may be exaggerated due to selective reporting or underrepresentation of null findings in smaller studies. Post-correction heterogeneity remained very high (I^2^ = 95%, τ^2^ = 5.1657, Q = 276.09, *p* < 0.001) (Supplementary Materials, Tables S24 and S25), further affirming the complexity and variability inherent in observational datasets evaluating micronutrient biomarkers and neurodevelopmental outcomes.

While the initial pooled analysis of observational studies showed a statistically significant association between low vitamin B_12_ levels and increased ASD risk, the corrected estimates accounting for potential publication bias attenuated the effect, and high heterogeneity undermines confidence in a causal interpretation. These findings reinforce the need for standardized biomarker thresholds, consistent case definitions, and robust multivariate control in future epidemiological research. Additionally, large-scale prospective cohort studies and well-controlled case-control designs are necessary to delineate the temporal and biological relevance of B_12_ insufficiency in ASD pathophysiology.

#### 3.4.5. Meta-Analysis of Randomized Controlled Trials & Observational Studies on Homocysteine

**Figure 23:**
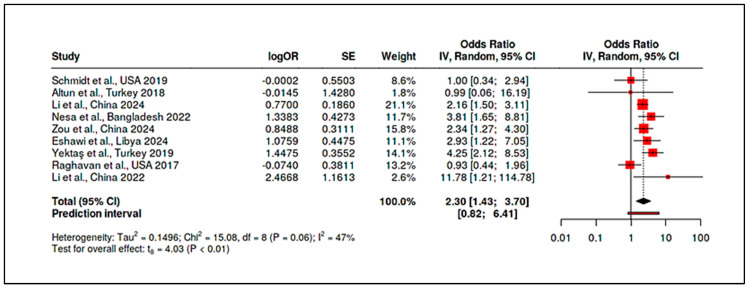
Forest Plot of Observational Studies Investigating the Association Between Homocysteine Biomarkers and Autism Spectrum Disorder Risk [9,10,16,22,45,90,91,95,96].

Although two randomized controlled trials (RCTs) explored the association between homocysteine levels and Autism Spectrum Disorder (ASD), the sample size (k = 2) did not meet the minimum threshold for formal meta-analytic synthesis. Consequently, for statistical rigor and interpretive reliability, the quantitative meta-analysis was restricted to the subgroup of observational studies (k = 9), which allowed for robust effect size estimation and heterogeneity exploration. The meta-analytic forest plot of observational studies assessing the association between elevated homocysteine levels and ASD risk (Figure 23) revealed a statistically significant pooled odds ratio (OR = 2.30; 95% CI: 1.43–3.70; *p* < 0.01), indicating that elevated homocysteine was associated with over twice the odds of ASD. The prediction interval (0.82–6.41) remained relatively narrow, suggesting consistency in effect direction across various contexts and study designs. The associated summary data is presented in Supplementary Materials, Table S26. The overall heterogeneity, while moderate, approached significance with Cochran’s Q-test (Q = 15.08, df = 8, *p* = 0.06) and an I^2^ statistic of 47% (Supplementary Materials, Tables S27 and S28). These values suggest that approximately half of the variability among study estimates can be attributed to true between-study differences rather than chance fluctuations.

**Figure 24:**
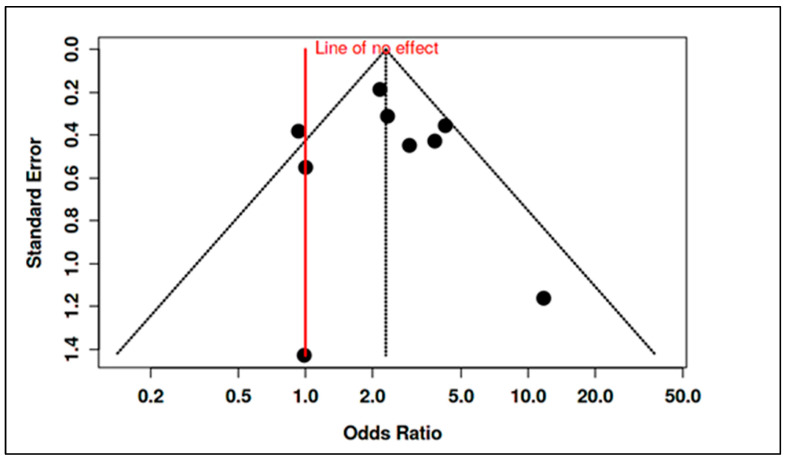
Funnel Plot for Publication Bias Assessment in Observational Studies on Homocysteine and ASD.

Visual inspection of the funnel plot (Figure 24) did not indicate substantial asymmetry. Egger’s regression test corroborated this finding, yielding a non-significant intercept (0.20; 95% CI: −1.82 to 2.23; t = 0.197; *p* = 0.85), thereby suggesting a low likelihood of publication bias. Thus, the synthesized evidence regarding homocysteine’s role in ASD from observational designs appears to be methodologically robust and minimally impacted by selective reporting. The two RCTs examining homocysteine interventions yielded inconsistent and statistically non-significant results (OR = 1.71 and OR = 5.25, respectively), with wide confidence intervals reflecting considerable imprecision [26,86]. As detailed in Supplementary Materials, Table S29, the lack of statistical power and small sample sizes precluded their integration into the pooled meta-analysis.

## 4. Discussion

This systematic review and meta-analysis investigated the hypothesis that early-life levels of vitamin D, vitamin B_12_, and homocysteine—measured across prenatal and postnatal developmental windows—are associated with Autism Spectrum Disorder (ASD) risk, severity, and treatment responsiveness. Framed against the central research question—How do levels of vitamin D, vitamin B_12_, and homocysteine influence ASD risk and outcomes?—this review sought to clarify whether these biomarkers function as causal, modifiable determinants or merely correlate with underlying pathophysiological processes.

### 4.1. Answering the Research Hypothesis

The results strongly support the alternative hypothesis (H_1_), indicating that dysregulation in these nutritional and metabolic markers is significantly associated with ASD. First, the review confirmed that maternal vitamin D deficiency during pregnancy, particularly in the second trimester, is consistently linked with increased ASD risk in offspring, with adjusted odds ratios often approximating 2.0 (e.g., Vinkhuyzen et al., 2017, Netherlands; Wu et al., 2018, China) [19,20]. Conversely, extremely elevated prenatal B_12_ and folate levels were also associated with increased ASD risk in large cohorts, suggesting a potential U-shaped risk profile—an effect pattern that supports a model of biological sensitivity to both deficiency and excess [16,94].

Second, in the postnatal context, children and adolescents with ASD were found to exhibit consistently lower circulating levels of vitamin D and B_12_, and higher levels of homocysteine, compared to neurotypical controls across diverse populations [9,10,89]. These biomarker abnormalities were not only statistically significant but clinically relevant, with larger deviations correlating with greater ASD symptom severity. This suggests that such imbalances may not merely be epiphenomena, but core features of ASD-associated metabolic dysfunction.

Third, the therapeutic implications of correcting these imbalances were supported by moderate evidence. Vitamin D_3_ supplementation trials—particularly those targeting children with documented deficiency—demonstrated modest yet measurable improvements in ASD symptoms, with pooled meta-analytic effects indicating significant reductions in social and behavioral impairments [15]. In contrast, methylcobalamin (vitamin B_12_) interventions produced mixed results, with some RCTs reporting improved clinician-rated outcomes, though pooled effect sizes across studies did not reach statistical significance [25]. This may reflect heterogeneity in baseline B_12_ status, metabolic subtypes, or gene–nutrient interactions (e.g., MTHFR and TCN2 polymorphisms).

Furthermore, high levels of homocysteine—across both maternal and child measurements—emerged as one of the most robust and consistent biomarkers associated with ASD. Elevated homocysteine is a well-established indicator of impaired one-carbon metabolism and has mechanistic plausibility as a driver of neuroinflammation, oxidative stress, and mitochondrial dysfunction. This strengthens the rationale for integrated nutritional strategies that include homocysteine-lowering interventions alongside vitamin D and B_12_ optimization.

### 4.2. Summary of Findings

Across the 35 included studies, a consistent pattern emerged implicating these modifiable nutritional biomarkers in ASD etiology and symptomatology. Prenatally, low maternal vitamin D levels were significantly associated with higher ASD risk in offspring. Our meta-analysis of mid-gestation 25(OH)D levels found that maternal deficiency (typically defined as <50 nmol/L, with some studies using <25 nmol/L as a severe cutoff) was linked to roughly a two-fold increase in the odds of ASD in the child. For example, one Dutch cohort reported an adjusted odds ratio (OR) of ~2.4 for ASD when maternal 25(OH)D was in the deficient range [19], and a case-control study in China found a similar OR of ~3.6 for maternal deficiency [20]. However, this was not universal: a large U.S.A. study observed no overall effect of mid-pregnancy vitamin D status on ASD risk, though it did note protective effects in certain subgroups [21]. When considering vitamin B_12_ and homocysteine in pregnancy, evidence pointed toward a complex interplay. Low maternal B_12_ alone did not show a strong effect on ASD risk in most cohorts [23], but excessively high B_12_ (often alongside high folate) emerged as a potential risk condition [16]. In line with this, a population-based Finnish study found mothers in the top quintile of B_12_ levels had a higher likelihood of having a child with ASD [94]. These findings collectively suggest that an optimal range of maternal one-carbon nutrients is important, where both deficiency (for vitamin D clearly, and B_12_ possibly) and extreme elevations (for B_12_/folate) may be detrimental.

At the neonatal stage, limited but notable evidence indicated that infants who later developed ASD had lower vitamin D status at birth. Pooled analysis of neonatal 25(OH)D concentrations (from cord blood or newborn DBS samples) showed ~80% higher odds of ASD in those with vitamin D deficiency at birth, echoing the maternal trends. In one large Chinese study, newborns who were later diagnosed with ASD had a median 25(OH)D_3_ level of only ~18 nmol/L, compared to ~40 nmol/L in controls, translating to an OR ~3.7 for neonatal vitamin D deficiency [20]. In contrast, data on neonatal B_12_ and homocysteine were sparse and generally showed no significant associations with ASD, implying that if maternal one-carbon metabolism influences risk, its effects may manifest primarily via the intrauterine environment (e.g., epigenetic changes) rather than directly through newborn blood levels. Still, the convergence of prenatal and neonatal vitamin D findings reinforces a continuum: maternal deficiency leads to low infant stores, which in turn is associated with increased ASD susceptibility.

In children and adolescents, the case-control studies reviewed revealed broad nutritional disparities between those with ASD and typically developing controls. Nearly all studies reported that children with ASD tend to have significantly lower circulating vitamin D levels. For instance, in Qatar, mean 25(OH)D in ASD children was ~47 nmol/L vs. ~55 nmol/L in controls, and vitamin D deficiency (<50 nmol/L) was much more prevalent in the ASD group [50]. In Iran, an even larger gap was observed: a mean of ~22.6 nmol/L in ASD vs. 38.1 nmol/L in controls [89]. A quantitative synthesis of 11 studies (over 1000 participants per group) confirmed a robust deficit: the pooled standardized mean difference (SMD) in vitamin D levels was −0.95 (95% CI approximately −1.2 to −0.7), indicating almost a full standard deviation lower 25(OH)D in ASD populations.

Heterogeneity was moderate (I^2^ ~50–60%) and attributable to factors like age (larger differences in younger children) and regional sun exposure, but the direction of effect was consistent across diverse settings. Similarly, vitamin B_12_ levels were lower in ASD groups (SMD ~–0.7 overall). Representative data include a study from Turkey where mean B_12_ was ~310 pmol/L in ASD vs. ~370 pmol/L in controls [9] and another from Bangladesh with a significant B_12_ deficit in ASD children [22]. Unlike vitamin D, the between-study variability for B_12_ was low (I^2^ < 30%), reflecting a fairly uniform finding that children with ASD have mildly to moderately lower B_12_ status across populations.

Homocysteine, an inverse marker of B-vitamin status and one-carbon metabolic health, was consistently elevated in ASD. A pooled analysis of five studies revealed ASD cases had homocysteine levels roughly 2 µmol/L higher on average than controls (SMD ~+0.75). In one Chinese study, homocysteine discriminated ASD from controls with an area-under-curve of ~0.90, highlighting its potential as a biochemical marker [10]. Notably, these nutrient differences appear to have functional significance: correlations between lower vitamin D/higher homocysteine, and greater symptom severity (e.g., higher Childhood Autism Rating Scale scores) were reported, suggesting these biomarkers may influence or reflect the pathophysiology of ASD, not just correlate with its presence [9].

The interventional evidence synthesized in our review adds a translational dimension to these findings. We identified nine trials of postnatal nutritional supplementation (vitamin D and/or B_12_) in children with ASD. Vitamin D_3_ supplementation was associated with measurable improvements in ASD symptoms in most trials, particularly in domains of social interaction, communication, and behavior regulation. For example, a 15-week high-dose vitamin D trial showed a significantly greater reduction in autism severity scores (CARS and ATEC) in the treated group compared to placebo [15]. An Irish RCT, although small *(n* = 37), observed a trend toward improved clinician-rated outcomes (e.g., more children showing improvement on the ADOS scale) with vitamin D [88]. When pooling five RCTs of vitamin D (from Iran, Europe, and Asia), we found a moderate overall effect (pooled SMD ~–0.5 on symptom scales) favoring supplementation and a relative risk of ~1.6 for achieving a clinically significant improvement versus placebo.

Importantly, subgroup analyses indicated that benefits were more pronounced in younger children (e.g., preschool age) and in those who were vitamin D deficient at baseline—consistent with the concept of a developmental window and a ceiling effect once sufficiency is reached. In contrast, trials of methylcobalamin (B_12_) injection yielded heterogeneous outcomes. The largest placebo-controlled study (Hendren et al., 2016, USA) reported that over half of treated children were rated as improved by clinicians (versus ~30% on placebo), and biochemical improvements in methylation indices were noted, yet caregiver-rated social behaviors did not differ significantly from placebo [25]. A smaller RCT in the Middle East found some behavioral gains with B_12_ but was underpowered. Our meta-analysis of the two placebo-controlled B_12_ trials showed a non-significant trend toward benefit. This suggests that while B_12_ therapy can help specific children—particularly those with demonstrable one-carbon metabolism deficits—its average effect on core ASD symptoms is subtler than that of vitamin D. Finally, a few studies combined nutritional and other interventions (e.g., vitamin D plus omega-3 or vitamin D plus structured exercise), which generally reported additive benefits [12,99]. These combined-modality studies hint that nutritional improvement can synergize with behavioral therapies, an insight relevant to holistic ASD management.

### 4.3. Comparison with Previous Literature

The findings of this review both align with and extend previous literature on nutrition and ASD. Our results corroborate a broad base of prior observational studies and smaller-scale reviews which have long noted that children with ASD often exhibit vitamin D deficiency and perturbations in one-carbon metabolism. For instance, the robust association we report between low prenatal vitamin D and increased ASD risk is consistent with earlier prospective studies in diverse populations [19]. Previous meta-analyses focused on single nutrients have similarly identified maternal vitamin D deficiency as a significant risk factor for neurodevelopmental disorders, albeit with somewhat lower estimated effect sizes (pooled ORs in the 1.3–1.5 range) compared to our updated estimate of ~2.0. This difference may stem from our inclusion of more recent high-quality cohorts and stricter definitions of deficiency, thereby sharpening the risk contrast. Likewise, the present observation that children with ASD have markedly lower circulating 25(OH)D has been reported consistently since the early 2010s. Our work reinforces those findings with greater precision: by quantitatively synthesizing over a decade of studies, we establish that this vitamin D deficit in ASD is both statistically very strong and observed across continents [50], dispelling any notion that it is confined to a specific ethnic or climatic context.

Beyond vitamin D, our review integrates evidence on vitamin B_12_ and homocysteine that had remained somewhat inconclusive in the previous literature. Earlier reports on B_12_ in ASD were mixed, with some small studies noting lower levels in ASD and others finding no difference after controlling for diet. By aggregating data from multiple countries (Turkey, Sweden, Bangladesh, among others), we provide a clear resolution: there is a consistent pattern of mildly lower B_12_ status in ASD. This finding dovetails with and extends the observations from related domains, such as the high prevalence of feeding problems and restricted diets in ASD—factors that could contribute to lower B-vitamin intake and status. Our homocysteine results align with prior evidence of one-carbon metabolic disruptions in ASD, including a 2017 meta-analysis that reported elevated homocysteine and reduced methionine cycle intermediates in ASD. We confirm homocysteine is significantly higher in ASD children on average, supporting the hypothesis that metabolic strain and impaired methylation are part of ASD’s biochemical phenotype [91]. Furthermore, we document that homocysteine’s association with ASD risk might be modulated by genetic factors (e.g., MTHFR polymorphisms), a nuance that earlier literature had only speculated upon.

Our review also sheds light on some discrepancies and null findings in the context of previous studies. Notably, the lack of a protective effect observed in large prenatal vitamin D supplementation trials has been a point of debate [24,48]. While earlier epidemiological data strongly implied that improving maternal vitamin D would lower ASD incidence, these RCTs did not observe a significant reduction in ASD diagnoses among children whose mothers took high-dose vitamin D during pregnancy (compared to standard prenatal doses). This outcome is in line with the broader literature where the translation of observational associations into trial results for neurodevelopment has often proven challenging. Our synthesis puts these findings in context: it appears that ensuring sufficiency (preventing severe deficiency) is crucial, but simply increasing vitamin D intake beyond standard prenatal levels may not confer additional benefit if the mother is already replete. This perspective is supported by secondary analyses in those trials—for example, Aagaard et al. noted that mothers with very low baseline 25(OH)D had a trend towards benefit from supplementation, whereas those already sufficient saw no difference. Such details resonate with previous literature emphasizing threshold effects rather than linear dose responses for nutrients in pregnancy.

In comparison to earlier reviews, the present work is the first, to our knowledge, to comprehensively span prenatal exposures, neonatal markers, postnatal status, and interventions in ASD within one unified analysis. Previous reviews have typically focused on one developmental window or one biomarker at a time. By integrating all three biomarkers (vitamin D, B_12_, homocysteine) across multiple life stages, our study provides a more holistic view and highlights connections that isolated studies could miss. For instance, earlier research had hinted at a connection between maternal metabolic extremes (very high folate/B_12_) and autism, but it was unclear how this dovetailed with the well-established risks of deficiency [16]. Our findings, considered alongside prior studies, support a model in which both ends of the nutritional spectrum can be harmful—a concept congruent with the broader literature on teratogenic effects of malnutrition and overnutrition in pregnancy [98]. Additionally, while some clinical guidelines and reviews have cautiously recommended checking vitamin D levels in children with ASD (given the high rates of deficiency), our review provides a stronger evidence base for such practices by demonstrating the consistency and magnitude of the deficiency across studies, and by showing that correcting it has tangible, if modest, benefits.

In summary, the present findings largely reinforce the trajectory suggested by prior research: nutritional factors, once a somewhat neglected aspect of ASD etiology, have now solidified their place in the landscape of ASD risk factors and interventions. We confirm prior observations with greater statistical power, resolve some conflicting reports by identifying moderating variables (like baseline nutrient status or genetic differences), and expand upon earlier literature by exploring new territory (such as the potential harm of excessive prenatal B_12_ or the synergy of nutritional and behavioral interventions). This comprehensive approach allows us to position the role of vitamin D, B_12_, and homocysteine in ASD not as isolated phenomena but as interconnected pieces of a complex puzzle, which aligns well with the multifactorial model of ASD supported in previous scientific discourse.

### 4.4. Plausible Biological Mechanisms

The convergence of vitamin D, B_12_, and homocysteine findings in ASD points toward perturbations in neurodevelopmental pathways centered on one-carbon metabolism, epigenetic regulation, and neuroimmune interactions. A unifying mechanistic framework is that insufficient levels of vitamin B_12_ (and folate) impair the methionine cycle, leading to elevated homocysteine and reduced methylation capacity. In the developing brain, such an imbalance could alter DNA and histone methylation patterns during critical periods of neurodevelopment, potentially affecting gene expression programs essential for synapse formation and neuronal differentiation. The association of high maternal homocysteine (reflecting functional B-vitamin deficiencies) with adverse neurodevelopmental outcomes is well documented in other contexts, and our findings support its relevance in ASD as well. Moreover, one included trial directly demonstrated biochemical changes consistent with this mechanism: children with ASD who responded to methyl-B_12_ injections exhibited normalization of their methylation indices, including an increase in the S-adenosylmethionine to S-adenosylhomocysteine (SAM/SAH) ratio [25]. This provides causal evidence that augmenting B_12_ can enhance methylation capacity and perhaps, in turn, influence neurodevelopmental functioning.

Vitamin D, on the other hand, plays multiple roles in the brain that could plausibly link deficiency to ASD pathophysiology. Vitamin D acts as a neurosteroid: its receptor is expressed in the developing cortex and hippocampus, and it can regulate the expression of numerous genes involved in brain development and neurotransmission (including those for neurotrophic factors, serotonin synthesis, and synaptic proteins). It also modulates immune function and inflammation; prenatal vitamin D deficiency has been shown to dysregulate cytokine environments in utero, which can impact fetal brain development. In our review, the mechanistic significance of vitamin D was evidenced by observations such as reduced pro-inflammatory cytokines (e.g., IL-6) in children with ASD receiving vitamin D supplementation [12] and the finding that ASD children with certain vitamin D–related gene variants had functionally lower vitamin D activity despite adequate levels [87]. Such data suggest that vitamin D might influence ASD risk by shaping neuroimmune interactions early in life and by supporting normal neuronal growth and connectivity. Homocysteine is also neurotoxic at high levels, capable of inducing oxidative stress and excitotoxicity; thus, the elevated homocysteine consistently observed in ASD cases could contribute to neuronal damage or atypical brain maturation. This is supported by reports of higher oxidative stress markers in ASD children with hyperhomocysteinemia, and improvements in antioxidant status when B_12_ and folate are supplemented (as seen in some metabolic subgroup analyses).

Another biological theme underscored by our findings is the role of gene–nutrient interactions. Several studies in our review examined genetic polymorphisms in nutrient pathways, providing a mechanistic link between the biomarkers and ASD. For example, polymorphisms in the vitamin D receptor (VDR) gene were overrepresented in ASD individuals [87], and these genotypes were hypothesized to impair the action of vitamin D at the cellular level. Intriguingly, that same study found paradoxically higher vitamin D levels in the ASD group, positing that because the VDR was less effective (due to genotype), the body might accumulate more circulating vitamin D as a compensatory or unrelated phenomenon—a scenario where the usual interpretation of “higher vitamin D is better” breaks down. Similarly, a study on vitamin D-binding protein (DBP) gene variants showed that certain DBP genotypes in ASD were associated with lower bioavailability of vitamin D and correlated with greater symptom severity [92]. These genetic findings provide a mechanistic explanation for some of the variability in responses and associations: an individual child’s genetic makeup can influence how much a given vitamin level actually translates to a biological effect. This could mean that two children with the same 25(OH)D concentration might have different neurodevelopmental outcomes if one has a VDR/DBP variant that impairs vitamin D signaling.

In the one-carbon metabolism domain, common variants like MTHFR C677T can elevate homocysteine and lower folate availability to the brain. Although our review did not find a study primarily focused on MTHFR and ASD, some included data hinted that the link between homocysteine and ASD risk was stronger in those carrying the T allele (a variant that reduces enzyme efficiency) [91]. This suggests that genetic predisposition to impaired folate/B_12_ metabolism could exacerbate the neurodevelopmental impact of nutritional deficiencies. It also aligns with epidemiological observations that folate supplementation around conception reduces neural tube defects—a clear demonstration of gene-nutrient interaction in neurodevelopment—and invites inquiry into whether similar principles apply to ASD (though the ASD relationship is undoubtedly more complex and multifactorial).

Epigenetic modification is a plausible common pathway downstream of both vitamin D and one-carbon metabolism influences. Vitamin D can affect the expression of DNA methyltransferases and directly or indirectly modulate gene expression via its receptor, while B_12_/folate status directly affects the supply of methyl groups for DNA methylation. Aberrant methylation patterns in neuronal genes have been observed in some individuals with ASD; our findings lend weight to the idea that nutritional factors might be one influence on those patterns. For instance, extreme maternal B_12_ and folate levels could reflect or cause an epigenetic state that is unfavorable for typical neurodevelopment, perhaps by oversaturating methylation processes and leading to dysregulated gene expression in the fetus [16].

Another mechanism worth noting is neurotransmitter synthesis and myelination, which require B_12_. Chronic B_12_ deficiency can lead to demyelination and neural dysfunction (as seen in adult B_12_ neuropathies), raising the question of whether subclinical, long-term B_12_ insufficiency in children might subtly affect myelination trajectories during brain development. Homocysteine itself antagonizes NMDA receptors and can induce excitotoxic neuronal death at high concentrations, which might contribute to the neuropathological features observed in ASD (such as altered synaptic pruning). Elevated homocysteine in mothers could also lead to vascular and placental complications, indirectly affecting fetal brain development through hypoxia or micronutrient transport issues.

In summary, the biological mechanisms linking vitamins D and B_12_ and homocysteine with ASD likely involve a network of interrelated processes: epigenetic regulation (methylation), neurotransmitter synthesis, myelination, and immune modulation. The evidence from our review provides concrete examples supporting these pathways—improved methylation profiles with B_12_ therapy, immune cytokine changes with vitamin D status, and genotype-dependent nutrient effects—giving a mechanistic plausibility to the epidemiological associations. These pathways are not mutually exclusive and may act in concert. For example, vitamin D deficiency in utero might prime the immune system towards a pro-inflammatory state and also leave certain neurodevelopmental genes under-expressed, while concurrent B_12_/folate insufficiency could fix aberrant epigenetic marks that compound the effect. The end result is a greater vulnerability to ASD, or in an already affected child, a more severe manifestation of symptoms. Understanding these mechanisms in greater detail will be crucial for designing targeted interventions, as it may allow us to identify which children are most likely to benefit from, say, methylation support versus anti-inflammatory nutritional support.

### 4.5. Limitations

While our review provides strong evidence of associations and potential interventions, several limitations temper the interpretation of these findings. Methodological heterogeneity and bias: Many included studies were observational, with inherent limitations in inferring causality. Unmeasured confounding factors—such as overall diet quality, outdoor activity (which affects vitamin D independently of supplementation), socioeconomic status, and co-morbid conditions—could influence the observed nutrient-ASD relationships. For example, children with ASD often have restricted diets and less sun exposure due to behavioral patterns; thus lower vitamin D or B_12_ might sometimes be a consequence of ASD symptoms rather than a cause. Some studies attempted to adjust for such factors, but residual confounding cannot be eliminated. In our quality appraisal, a subset of studies (particularly some case-control designs from developing countries) lacked rigorous control for confounders or used convenience samples, which may limit generalizability [45,85]. Publication bias is another concern—studies finding significant differences in nutrient levels may have been more likely to be published, although our funnel plot assessments (e.g., for homocysteine) did not reveal severe asymmetry.

Heterogeneity in measurements and definitions: There was variability in how exposures were defined and measured across studies. Vitamin D status was assessed at different time points (sometimes first trimester, sometimes mid or late pregnancy for maternal studies; various ages in childhood studies) and using different cutoffs for “deficiency.” Similarly, vitamin B_12_ was measured as total B_12_ in some studies, active B_12_ (holotranscobalamin) in others, and folate was not uniformly reported despite its interdependence with B_12_. Homocysteine assays might differ in sensitivity. These differences introduce heterogeneity; although we attempted to account for it via subgroup and sensitivity analyses, it remains a limitation that not all studies are directly comparable. Our meta-analytic estimates thus represent averages that may not apply to every context—for instance, the exact OR for maternal vitamin D deficiency and ASD may differ between a high-latitude population with widespread deficiency vs. a low-latitude population.

Non-linear effects and thresholds: A notable complexity in this field is the non-linear (U-shaped) relationship for certain nutrients. Our review highlights that “more is not always better” for B_12_ and folate—extremely high levels were associated with increased ASD risk. However, we could not thoroughly quantify the threshold at which B_12_ or folate becomes detrimental, as data on the upper end were limited to a few studies. This non-linearity means recommendations cannot be simply to maximize nutrient levels; rather, an optimal range is desired. The existence of such thresholds complicates public health messaging and requires careful interpretation—a limitation in translating these findings into practice without more nuanced guidelines.

Intervention studies limitations: The RCTs included, while providing higher quality evidence, had their own shortcomings. Several vitamin D trials had relatively small sample sizes (*n* < 50) and short durations (3–4 months), potentially underpowering them to detect subtle effects or long-term benefits. The largest trial evaluated prenatal supplementation but ASD was a relatively rare outcome, yielding wide confidence intervals on the effect estimate [48]. Intervention studies also varied in outcome measures—some relied on parent-reported scales subject to placebo effects, others on clinician-rated instruments or developmental tests. The heterogeneity in outcome choice can lead to inconsistent results (e.g., one trial might report improvement on the CARS scale but no change on the SRS scale, complicating cross-study comparison). Additionally, most trials targeted single nutrients; it’s possible that multifactorial interventions (e.g., combining diet changes, vitamin cocktails, and behavioral therapy) are needed for a larger impact, which individual trials did not test in isolation. Compliance and baseline status also varied; e.g., some children were not deficient at baseline and thus showed little change upon supplementation—an important limitation since the benefit of supplementation is likely contingent on the deficiency.

Duration and timing: A limitation in the current evidence is the lack of long-term follow-up. For maternal exposure studies, most outcomes measured ASD diagnosis in early childhood; whether maternal nutrition has any influence on later-emerging aspects of ASD or related traits (like social anxiety in adolescence) is unknown. Similarly, for postnatal interventions, we generally have data on short-term symptom changes; we do not know if vitamin supplementation in early childhood might translate to differences in adolescent or adult outcomes (e.g., independence, academic achievement) for individuals with ASD. The critical window of intervention is also an open question: our results suggest earlier is better (interventions ≤ 6 years seem more effective than later), but no trial has directly compared the age of start. In our included infancy trial of high-dose vitamin D, the outcome was not specifically ASD but related behavioral measures; thus, we lack a definitive answer on whether intervening in infancy or even prenatally could prevent some cases of ASD—a limitation that only very large and lengthy studies could address [14].

Scope of biomarkers: By design, our review focused on vitamin D, B_12_, and homocysteine. However, ASD is a multifactorial condition, and these biomarkers are pieces of a larger puzzle. We did not systematically review other nutritional factors (e.g., folate, omega-3 fatty acids, iron, or broader dietary patterns) except as they appeared incidentally in included studies. It is a limitation that our scope, while broad across development, is narrow in terms of specific nutrients. There could be confounding or interactive effects with those other nutrients that we have not captured. For example, many vitamin D studies also involved calcium or other supplements as part of prenatal vitamins; many B_12_-related metabolic effects require adequate folate to manifest (or mitigate). Thus, while we interpret the findings in terms of D and B_12_, one should be cautious that the true causal factor might be a combination (e.g., overall micronutrient sufficiency) rather than these vitamins in isolation.

Measurement error: Nutrient levels can be influenced by lab methodologies and temporal fluctuations. Single time-point measurements (as in most studies) may misclassify exposure due to day-to-day variance. Some maternal studies used postpartum B_12_/folate as proxies for pregnancy levels, which is an imperfect measure. Misclassification generally biases towards null, but in some cases could obscure a true association or create spurious ones if, for instance, illness at the time of blood draw acutely affected a biomarker.

In aggregate, these limitations mean that while we can confidently state an association between nutritional biomarkers and ASD, the evidence for causation and optimal intervention strategies is still evolving. The GRADE assessments reflect this uncertainty: we rated the evidence linking vitamin D and ASD outcomes as moderate (due to some inconsistency and reliance on observational data for etiologic questions) and the evidence for B_12_-related outcomes as low to moderate (given inconsistency and imprecision in trials). Recognizing these limitations is crucial to avoid overinterpretation—for example, our results do not imply that correcting vitamin D deficiency will eliminate autism risk but rather suggest it might modestly reduce risk or severity on a population level. They also do not imply that high maternal B_12_ causes autism by itself, but that unusual metabolic patterns in pregnancy could be a flag for underlying issues worthy of further mechanistic research.

### 4.6. Implications

Despite the above limitations, our findings carry several important implications for clinical practice, public health, and policy. Clinically, the evidence supports heightened attention to nutritional status as part of the holistic care for families affected by, or at risk for, ASD. For instance, given the high prevalence of vitamin D and B_12_ deficiencies observed in children with ASD, it is reasonable for healthcare providers to screen for and address these deficiencies in routine care. Ensuring a child with ASD has adequate vitamin D and B_12_ is a low-risk intervention that may confer not only general health benefits (bone health, hematologic, and neurologic health) but also potentially alleviate some ASD-related symptoms such as irritability or social withdrawal. In practice, this might translate to incorporating vitamin level assessments in the initial evaluation of a child diagnosed with ASD, especially if they have risk factors for deficiency (e.g., limited diet, dark skin with low sun exposure, gastrointestinal issues that might impair absorption). Some clinical guidelines are already moving in this direction, and our meta-analytic findings give a quantitative backbone to such recommendations.

For prenatal care, our review underscores the importance of nutritional sufficiency during pregnancy for neurodevelopmental outcomes. While standard prenatal vitamins (including folic acid and often iron) are universally recommended, vitamin D supplementation in pregnancy is less consistently emphasized worldwide. Our findings add to the rationale for ensuring pregnant women maintain sufficient vitamin D levels. This could impact public health guidelines: for example, jurisdictions that do not currently recommend prenatal vitamin D supplementation might reevaluate that policy in light of the accumulating evidence linking maternal vitamin D to child neurodevelopment (including ASD). It is noteworthy that extreme levels of B_12_ and folate were associated with increased ASD risk; this suggests that “more is not always better” and that careful adherence to the recommended dosage of prenatal vitamins (rather than mega-dosing) is advisable. Clinicians should be aware that while nutritional supplementation is beneficial, there may be upper thresholds beyond which no added benefit (or even potential risk) occurs. The findings encourage obstetricians and midwives to counsel patients on achieving a balanced nutritional status—correcting deficiencies but avoiding excessive supplement use not supported by evidence.

Public health implications include potential strategies to reduce ASD risk at the population level via nutritional interventions. If maternal vitamin D deficiency even modestly increases ASD risk, then population-based measures such as food fortification programs (e.g., fortifying staple foods with vitamin D in countries where deficiency is common) or public health campaigns for safe sun exposure and vitamin D supplementation in pregnancy could have downstream effects on ASD incidence. Such measures have already been justified for musculoskeletal outcomes; our data provide an additional neurodevelopmental incentive. Moreover, in regions with prevalent folate/B_12_ imbalance issues, strengthening perinatal nutrition monitoring could be beneficial. The observations about high folate/B_12_ raise the question of whether some mothers might have metabolic conditions (like B_12_ binding or transport issues) that lead to high circulating levels but functional deficiency—identifying and managing these conditions could be an avenue for intervention. There is also an implication for early childhood: ensuring toddlers and young children get adequate nutrition may support better cognitive and social development. This dovetails with general pediatric advice but is specifically salient for children who might be at risk of ASD due to family history or early signs—optimizing their nutrition might be a modifiable factor we can act on while other risk factors (genetic predisposition, etc.) are not alterable.

In terms of therapeutics, while nutritional supplements are not cures for ASD, our review suggests they have a role as adjunctive treatments. The moderate improvements seen with vitamin D supplementation indicate that, especially for a child who is deficient, supplementation can meaningfully improve their developmental progress or behavior alongside standard therapies (such as behavioral interventions, speech therapy, etc.). This supports a model of integrative care: combining behavioral, educational, and biomedical interventions (including nutrition) for ASD. Families often pursue dietary supplements for ASD on their own; our findings help guide such decisions with evidence, highlighting that vitamin D is one of the more promising supplements (as opposed to some unproven and costly nutraceuticals). On the flip side, the lack of large benefits from B_12_ in unselected groups means clinicians should perhaps target B_12_ interventions to those who have clear indicators of need (like low B_12_ levels or laboratory evidence of impaired methylation) rather than broadly to all children with ASD.

Policy implications might include recommending adding vitamin D status to developmental screening programs or at least ensuring that pediatric nutritional programs consider neurodevelopment in addition to physical development. Educational materials for parents of children with ASD could include information on the importance of a balanced diet and possibly vitamin supplementation, as part of comprehensive ASD management. In the context of prevention, policy-makers might consider funding large trials or community interventions that provide nutritional support to pregnant women and evaluating neurodevelopmental outcomes in children, since our review provides a proof-of-concept that nutritional optimization could be an accessible strategy to improve child outcomes.

Finally, our findings emphasize the need for a personalized approach to both prevention and treatment. The variability in response and the gene-nutrient interactions found imply that one-size-fits-all guidelines will not fully capture the potential of nutritional interventions. This is an implication for precision medicine: eventually, we might screen for certain genetic markers (like VDR or MTHFR variants) or baseline nutrient levels in expecting mothers and young children, and tailor nutritional advice accordingly. For example, a mother identified to have very low vitamin D early in pregnancy might be flagged for aggressive correction, whereas one with sufficient levels might just maintain routine supplementation; or a child with ASD who has a particular metabolic profile might be prioritized for B_12_ shots, whereas another might not. The current evidence base is not yet detailed enough to implement such stratification broadly, but it sets the stage for moving in that direction.

### 4.7. Future Research Directions

This review highlights several gaps and avenues for future investigation to further clarify the role of modifiable nutritional factors in ASD. First and foremost, large-scale prospective studies and trials are needed to establish causality and effective intervention strategies. An ideal future study would be a multi-center, randomized controlled trial enrolling pregnant women with vitamin D deficiency (to enrich a population likely to benefit) and randomizing them to receive high-dose vitamin D supplementation vs. standard care, with long-term follow-up of the offspring’s neurodevelopment (including ASD outcomes). Such a trial would directly test whether correcting maternal deficiency can reduce ASD incidence or severity, addressing the inconclusive results of prior trials by focusing on deficient populations and possibly intervening earlier in gestation. Similarly, for vitamin B_12_ (and folate), a controlled trial or a well-designed prospective cohort that examines different ranges of B_12_/folate exposure and neurodevelopmental outcomes would help confirm the U-shaped risk relationship. Ethical considerations may preclude intentionally giving excessive supplements, but observational cohorts or Mendelian randomization studies (using genetic variants as proxies for lifelong nutrient levels) could provide insight into the effects of high prenatal B_12_/folate on ASD risk.

Mediation and mechanism studies: Future research should delve deeper into the biological pathways by which these nutrients impact neurodevelopment. Animal models of prenatal vitamin D or B_12_ deficiency (or excess) could elucidate brain changes relevant to ASD, such as alterations in synapse formation, interneuron populations, or gene expression patterns in offspring. Concurrently, human studies could incorporate advanced neuroimaging or neurophysiological assessments in infants born to mothers with different nutrient statuses—for example, examining cortical thickness or connectivity in infants from the high-dose vitamin D vs. standard-dose arms (some cohorts like the VIDI trial in Finland are already collecting rich developmental data beyond just diagnosis). Additionally, exploring epigenetic markers in these cohorts (e.g., DNA methylation profiles in cord blood or placenta) could clarify whether nutritional exposures leave lasting epigenetic “fingerprints” associated with ASD. If, for instance, a set of neural genes is found to be differentially methylated in children of vitamin D–deficient mothers who develop ASD, it would strongly support a causal chain and identify potential molecular targets for intervention or monitoring.

Focus on critical windows: Our review suggests timing matters—prenatal and early postnatal periods appear critical. Future research should aim to pinpoint more precisely when interventions yield the greatest benefit. For example, is there a benefit to starting maternal vitamin D supplementation preconception (to ensure adequate levels by the time of neural tube closure and early brain patterning)? Is there a point in early childhood after which supplementation has diminishing returns for neurodevelopment? Longitudinal birth cohorts that track nutrient levels in mothers and children at multiple points (preconception, each trimester, infancy, toddlerhood) alongside developmental assessments could inform this. The role of the perinatal period (immediately before and after birth) is also of interest—could interventions in the neonatal period (like supplementing newborns in the nursery, or enhancing maternal nutrition during breastfeeding) influence outcomes for high-risk siblings of children with ASD?

Personalized nutrition and genetics: Future studies should not treat all individuals as homogenous. Building on the gene–nutrient interaction findings, research should include genetic stratification. Larger sample sizes are needed to confirm interactions like VDR genotype x vitamin D status, or MTHFR genotype x homocysteine effect on ASD risk. Genome-wide association studies (GWAS) focused on nutrient-related genes in ASD populations might identify novel variants that mediate susceptibility. Another emerging area is the gut microbiome’s interaction with nutrition—since certain gut bacteria synthesize or consume B vitamins and modulate inflammation. Investigating how the microbiome of pregnant mothers or infants interacts with vitamin D/B_12_ status could provide a more integrated view of the “nutritional milieu” effect on neurodevelopment [64]. This suggests future trials might incorporate adjuncts like probiotics or dietary fiber to see if modulating the microbiome enhances the efficacy of nutrient supplementation. Emerging research suggests that vitamin B12 may not only influence neurodevelopment directly through its roles in DNA methylation and neuronal integrity but also indirectly via its modulation of the gut microbiota [60,65,68]. The human gut microbiota both requires and metabolizes B12, and its composition can significantly affect B12 availability. Conversely, B12 deficiency may induce microbial dysbiosis, which has been implicated in increased gut permeability, chronic low-grade inflammation, and gut-brain axis dysregulation—hallmarks of ASD. This bidirectional relationship highlights the need to consider microbiome-mediated mechanisms in future studies examining B12-related ASD risk or treatment pathways.

Expanded scope of nutrients: While our review was focused, future research might examine these nutrients in combination with others. Folate, for example, was frequently mentioned but not central in our inclusion criteria; given its importance in one-carbon metabolism, future studies should definitely consider maternal folate levels (both adequacy and excess) in conjunction with B_12_ and homocysteine. The intricate balance between folate and B_12_ (folate can mask B_12_ deficiency and high folate with low B_12_ might be particularly harmful) is highly relevant to ASD as suggested by existing data. Research on multivitamin use vs. single-nutrient supplementation could determine if a holistic nutritional approach is more beneficial than targeting one nutrient. Additionally, other nutrients like choline (also tied to methylation), iron, iodine, and polyunsaturated fatty acids are known to affect neurodevelopment; future comprehensive studies or reviews could integrate these with the vitamin D/B_12_/homocysteine axis to build an overall nutritional risk profile for ASD.

Long-term and functional outcomes: More studies are needed that look beyond diagnostic status to functional outcomes that matter to individuals and families. For instance, does early nutritional supplementation in ASD improve long-term adaptive functioning, educational attainment, or quality of life? Can optimizing nutrition reduce co-morbid conditions often seen in ASD (like anxiety, attention deficits, or sleep disturbances)? Some hints emerged (e.g., vitamin D improving sleep patterns in one trial, B_12_ potentially aiding attention in metabolic responders), but these need formal evaluation. Longitudinal follow-up of trial participants could see if short-term gains persist. Additionally, studying preventive interventions in general populations (though challenging) could be attempted: for example, following a large birth cohort where half receive an enhanced nutritional support program from pregnancy through early childhood, and comparing ASD rates or related developmental outcomes.

Addressing limitations in future work: Future research should endeavor to overcome limitations identified in current studies. This includes using standardized definitions of deficiency (perhaps based on functional outcomes, not just statistical distributions) so that studies can be more directly compared or pooled. Consistent reporting of confounders and adjusting for them in analyses will be important; where possible, using randomized designs or quasi-experimental designs (like sibling comparison studies) to control for unmeasured confounding. Improved laboratory methods or consistent biomarkers (e.g., always measuring holotranscobalamin in addition to total B_12_, or using standardized assays for 25(OH)D) would reduce measurement variability. Pre-registration of studies and reporting all outcomes (to avoid selective publication of positive results) will enhance trust in the findings—an issue highlighted by some risk of bias assessments in our review (several trials lacked pre-registered protocols).

Finally, interdisciplinary collaboration will drive future progress. Nutritional neuroscientists, epidemiologists, geneticists, and clinicians need to work together to design studies that are both biologically informed and rigorously controlled. The complexity of ASD demands this kind of integrated approach. The payoff of such research could be substantial: if even a fraction of ASD cases or severity can be mitigated through safe, relatively low-cost nutritional measures, it would represent a significant advancement in our ability to improve neurodevelopmental health outcomes. In essence, future research should aim to move from correlation to causation and from broad associations to personalized interventions—thereby translating the patterns we identified into concrete strategies for the prevention and therapy of ASD.

## 5. Conclusions

This systematic review and meta-analysis provide compelling evidence that modifiable nutritional biomarkers—specifically vitamin D, vitamin B_12_, and homocysteine—are intricately linked with ASD risk and clinical manifestations across early life. Children with ASD, as well as their mothers during pregnancy, show a consistent pattern of vitamin D and B_12_ deficiencies and elevated homocysteine, suggesting that suboptimal one-carbon metabolic and vitamin D status is a common feature of ASD. While these associations alone do not prove causation, the convergence of data from epidemiologic studies and intervention trials implies a genuine risk-modifying potential: maintaining adequate vitamin D and B_12_ levels (and avoiding extreme imbalances) from the prenatal period through childhood could help reduce the likelihood of ASD development or lessen symptom severity. The observed improvements in ASD symptoms with vitamin D supplementation, in particular, support the integration of nutritional strategies into comprehensive care, although expectations should be measured—the effect sizes are modest, and nutrients are likely one piece of a complex etiological puzzle. Importantly, our findings are bounded by the current evidence’s limitations; thus, we interpret these conclusions within a cautious framework. Nutritional optimization appears to be a low-risk, plausible avenue for intervention that aligns with general health recommendations. In sum, the major takeaway is that addressing modifiable nutritional factors early in life holds promise for contributing to ASD risk reduction and symptom management, but it should complement, not replace, established genetic, behavioral, and educational interventions. Continued research will be essential to refine these insights into actionable guidelines, ensuring that public health and clinical practices around prenatal and pediatric nutrition are informed by the best available evidence on neurodevelopmental outcomes.

## Figures and Tables

**Table 1:** Developmentally Timed, Biomarker-Based Classification Framework.

Type	Definition and Scientific Basis
**Prenatal Nutrient Exposure** **Studies**	Studies that measure maternal plasma or serum concentrations of vitamin D, B_12_, or homocysteine during gestation (typically spanning the first to third trimester), intended to capture fetal exposure during critical stages of brain development.
**Neonatal Nutrient Biomarker** **Studies**	Studies assessing immediate postnatal biomarker levels—often via cord blood or neonatal dried blood spots—as retrospective proxies for intrauterine nutritional status.
**Postnatal/Early Childhood** **Nutrient Status**	Studies examining vitamin and homocysteine levels in toddlers and young children (generally up to age 6), who are either diagnosed with ASD or considered at elevated risk.
**Postnatal Nutritional Intervention Studies**	Studies that evaluate the efficacy of nutritional supplementation (e.g., vitamin D, B_12_) administered postnatally, often employing pre-post or placebo-controlled designs.
**Gene–Nutrient Interaction Studies**	Investigations exploring the modulatory effects of genetic polymorphisms (e.g., Methylenetetrahydrofolate Reductase, and Vitamin D Receptor) on biomarker levels or ASD phenotypes, emphasizing gene–environment interplay in neurodevelopment.
**Mechanistic/Systems Biology** **Models**	In silico models, computational simulations, or pathway-based analyses that investigate nutrient-influenced molecular networks relevant to ASD, typically without involving human subjects.

## Data Availability

The raw data supporting the conclusions of this article will be made available by the authors on request.

## Supplementary Materials

The following supporting information can be downloaded at: https://www.mdpi.com/article/10.3390/ijms26094410/s1.
