# Supplementary material for: Modifiable Nutritional Biomarkers in Autism Spectrum Disorder: A Systematic Review and Meta-Analysis of Vitamin D, B12, and Homocysteine Exposure Spanning Prenatal Development Through Late Adolescence
Source: Int J Mol Sci. 2025 May 6;26(9):4410. doi: 10.3390/ijms26094410 (PMC12072606; doi:10.3390/ijms26094410)
Supplement: Supplementary file 1 [file ijms-26-04410-s001.zip › ijms-3612163-supplementary.pdf]

Article

# Modifiable Nutritional Biomarkers in Autism Spectrum Disorder: A Systematic Review and Meta-Analysis of Vitamin D, $B_{12}$, and Homocysteine Exposure Spanning Prenatal Development through Late Adolescence

**Oana-Elisabeta Avram [1], Elena-Alexandra Bratu [1], Cecilia Curis [2], Lavinia-Alexandra Moroianu [3,*] and Eduard Drima [4]**

1. Doctoral School of Biomedical Sciences, “Dunărea de Jos” University, 800201 Galați, Romania; titeoana@yahoo.com (O.-E.A.); alexandra.brt99@gmail.com (E.-A.B.)
2. Medical Department, Faculty of Medicine and Pharmacy, Dunărea de Jos University, 800201 Galați, Romania; cecilia_curis@yahoo.com
3. Department of Pharmaceutical Sciences, Faculty of Medicine and Pharmacy, Dunărea de Jos University, 800201 Galați, Romania
4. Clinical Medical Department, Faculty of Medicine and Pharmacy, Dunărea de Jos University, 800201 Galați, Romania; drima_edi1963@yahoo.com

* Correspondence: lavinia.moroianu@yahoo.com

## SUPPLEMENTARY MATERIALS

**Table S1**. Computational Equations Applied for Data Extraction and Meta-Analytic Estimations.

| Formula | Equation |
| --- | --- |
| **Estimating the Experimental Group Mean (Reverse Calculation from SMD):** | **(1)** |

$$\mu_{Exp} = \mu_{Control} + SMD \times \sigma_{pooled}$$

Where:

- **μExp** → Estimated Mean of the Autistic Spectrum Disorders (ASD experimental) group.
- **μControl** → Reported Mean in the control group.
- **SMD** → Standardized Mean Difference.
- **σpooled** → Pooled Standard Deviation (SD), assumed equal between groups.

| Formula | Equation |
|---|---|
| **Estimating Standard Deviation from Confidence Interval and Sample Size:** $$\mathbf{SD} = \frac{\mathbf{Upper\ CI} - \mathbf{Lower\ CI}}{\mathbf{2} \times \mathbf{t}} \times \sqrt{\mathbf{n}}$$ Where: <br>• $\mathbf{Upper\ CI}, \mathbf{Lower\ CI}$ → Upper and Lower bounds of the Confidence Interval. <br>• $\mathbf{t}$ → Critical t-value for a given confidence level and degrees of freedom. <br>• $\mathbf{n}$ → Sample size of the respective group. | **(2)** |
| **Estimating the Control Group Mean (Assuming Symmetrical Effect):** $$\boldsymbol{\mu}_{\mathbf{Control}} = \boldsymbol{\mu}_{\mathbf{Exp}} - \mathrm{SMD} \times \boldsymbol{\sigma}_{\mathbf{pooled}}$$ This is the algebraic inverse of **Equation (1)**, used when the experimental mean is known. | **(3)** |
| **Estimating Standard Deviation from Event Rate (Binary Outcome):** $$\mathbf{SD} = \sqrt{\mathbf{p} \cdot (\mathbf{1} - \mathbf{p}) \cdot \mathbf{n}}$$ Where: <br>• $\mathbf{p}$ → Proportion of Events (Event Rate) in the Control group. <br>• $\mathbf{n}$ → Sample size of the group. | **(4)** |
| **Calculating Standardized Mean Difference (Hedges' g)**: $$\mathbf{g} = \frac{\mathbf{M}_{\mathbf{Exp}} - \mathbf{M}_{\mathbf{Control}}}{\mathbf{SD}_{\mathbf{pooled}}}$$ Where: <br>• $\boldsymbol{M}_{\mathbf{Exp}}, \boldsymbol{M}_{\mathbf{Control}}$ → Group Means. <br>• $\boldsymbol{SD}_{\mathbf{pooled}}$ → Pooled Standard Deviation. | **(5)** |
| **Calculating the Pooled Standard Deviation:** $$\mathbf{SD}_{\mathbf{pooled}} = \sqrt{\frac{(\mathbf{n}_{\mathbf{Exp}} - \mathbf{1}) \cdot \mathbf{SD}^{\mathbf{2}}_{\mathbf{Exp}} + (\mathbf{n}_{\mathbf{Control}} - \mathbf{1}) \cdot \mathbf{SD}^{\mathbf{2}}_{\mathbf{Control}}}{\mathbf{n}_{\mathbf{Exp}} + \mathbf{n}_{\mathbf{Control}} - \mathbf{2}}}$$ Where: <br>• $\mathbf{SD}_{\mathbf{Exp}}, \mathbf{SD}_{\mathbf{Control}}$ → Standard Deviations of each group. <br>• $\mathbf{n}_{\mathbf{Exp}}, \mathbf{n}_{\mathbf{Control}}$ → Sample sizes | **(6)** |

| Formula | Equation |
|---|---|
| **Confidence Interval for SMD**: $$\mathbf{CI = g \pm t_{\alpha/2} \cdot SE}$$ Where: <br>• $\mathbf{g}$ → Hedges' g (SMD).<br>• $\mathbf{t_{\alpha/2}}$ → Critical t-value.<br>• $\mathbf{SE}$ → Standard Error of SMD. | **(7)** |
| **Standard Error of SMD (Hedges' g):** $$\mathbf{SE = \sqrt{\frac{1}{n_{Exp}} + \frac{1}{n_{Control}} + \frac{g^2}{2\left(n_{Exp} + n_{Control}\right)}}}$$ This SE is used to compute the confidence interval in **Equation (7).** | **(8)** |
| **Weight for Meta-Analysis (Inverse Variance Weighting):** $$\mathbf{w = \frac{1}{SE^2}}$$ Where, SE is the standard error of the SMD or log OR, depending on the outcome type. | **(9)** |

**Notes:**

- Where values are not explicitly reported, systematic review guidelines (Cochrane Handbook, MOOSE, PRISMA 2020) permit scientific inference or imputation under certain conditions. Common Estimation Methods:
  - **Standard Deviation (SD)**: Can be derived from Standard Error (SE), Confidence Intervals, Interquartile Range (IQR), p-values, or from similar studies using pooled SDs.
  - **Event Rates**: Can be estimated from proportions, percentages, or total Ns if provided graphically or textually.
  - **Effect Sizes (SMD/OR)**: Can be calculated using group means + SDs, raw event data, or from test statistics (e.g., t-values).
  - **Confidence Intervals**: Can be back-calculated from standard errors, sample sizes, or effect size ± margin error.
  - **Weights in Meta-Analysis**: Automatically computed once SMD or OR and SE are known — typically derived in forest plots or meta-regression tools (RevMan, meta, metafor).

**Citation:** All references are cited in the main text.

**Table S2**. Comprehensive Search Strategies by Database for the Systematic Review.

| Search ID | Access Platform | Primary Database | Type of Resources Covered | Search Terms Strategy |
|---|---|---|---|---|
| **1** | NCBI PubMed. | MEDLINE / PubMed Records. | Peer-reviewed articles, biomedical literature. | A comprehensive Boolean search combining controlled vocabulary and free-text terms:<br>**ASD terms**: ("autism" OR "autism spectrum disorder" OR "ASD" OR "pervasive developmental disorder");<br>**Nutrient/Biomarker terms**: ("vitamin D" OR "25-hydroxyvitamin D" OR "cholecalciferol" OR "vitamin B12" OR "cobalamin" OR "homocysteine" OR "folate" OR "one-carbon metabolism");<br>**Population**: ("pregnancy" OR "prenatal" OR "maternal" OR "infant" OR "child" OR "early life");<br>**Filters Applied:** Date range: 2015–2025, Free Full Text, Humans, Clinical Trial, Randomized Controlled Trial. |
| **2** | Cochrane Library. | CENTRAL (Cochrane Central Register of Controlled Trials). | Clinical trials, Cochrane Reviews, protocols. | Keyword and MeSH term combinations tailored to Cochrane indexing:<br>**Condition terms**: ("autism spectrum disorder" OR "ASD" OR "autism");<br>**Nutrient/Biomarker terms**: ("vitamin D" OR "25-hydroxyvitamin D" OR "cholecalciferol" OR "vitamin B12" OR "homocysteine" OR "folate" OR "one-carbon metabolism");<br>**Life stage terms**: ("pregnancy" OR "prenatal" OR "maternal" OR "infant" OR "child").<br>**Filters Applied:** Date range: 2015–2025, Humans, Clinical Trials, Cochrane Reviews. |
| **3** | Google Scholar. | Aggregated Scholarly Sources. | Scholarly articles, theses, books, conference papers. | Free-text Boolean search refined by title filtering:<br>**Main condition**: "autism spectrum disorder";<br>**Nutrient/Biomarker**: ("vitamin D" OR "vitamin B12" OR "homocysteine");<br>**Population scope**: ("pregnancy" OR "prenatal" OR "early life");<br>**Study type filter (title level)**: Date range: 2015–2025. |

| Search ID | Access Platform | Primary Database | Type of Resources Covered | Search Terms Strategy |
|---|---|---|---|---|
| **4** | ClinicalTrials.gov | ClinicalTrials.gov Registry | Registered clinical trials | Clinical condition and intervention fields used in advanced search:<br>**Condition**: "Autism Spectrum Disorder";<br>**Interventions**: ("vitamin D" OR "vitamin B12" OR "homocysteine");<br>**Filters applied**: Interventional studies (Clinical Trials);<br>**Status:** Recruiting or Completed;<br>**Time frame:** 2015–2025. |
| **5** | ProQuest (Dissertations & Theses / Central) | ProQuest Grey Literature Databases | Unpublished research, dissertations, theses, reports | Free-text Boolean search adapted for unpublished and grey sources:<br>**Condition keywords**: ("autism" OR "ASD");<br>**Nutritional exposure terms**: ("vitamin D" OR "vitamin B12" OR "homocysteine");<br>**Life stage filters**: ("pregnancy" OR "early life");<br>**Publication types**: Dissertations, Theses, Conference Proceedings, Scholarly Journals;<br>**Time filter**: Last 10 years. |

**Citation:** All references are cited in the main text.

**Table S3.** Summary of Studies Included in the Qualitative and/or Quantitative Evidence Synthesis.

| No. | Authors (Year, Country) | Study Design | Systematic Review (SR) | Meta-Analysis (MA) | Support for Decision |
|---|---|---|---|---|---|
| 1 | Aagaard et al., 2024, Denmark | Randomized Controlled Trial (RCT) | ✅ Included | ✅ Included | RCT assessing prenatal vitamin D supplementation; eligible for both qualitative synthesis and meta-analysis; full data on sample size, means, and event rates available. |
| 2 | Altun et al., 2018, Turkey | Observational (Case-Control) | ✅ Included | ✅ Included | Eligible observational study with neurotypical control group, quantifiable biomarker levels (vitamin D, B12, homocysteine, others), standardized assays, and matched sampling design; full data on means, SDs, and sample sizes available for meta-analytic synthesis. |
| 3 | Arastoo et al., 2018, Iran | Observational (Case-Control) | ✅ Included | ✅ Included | Included due to availability of complete case-control data, serum vitamin D values, standard measurement protocol (ELISA), and statistical outcomes (means, SDs, p-values); fulfills inclusion criteria for both synthesis types. |
| 4 | Bener et al., 2017, Qatar | Observational (Case-Control) | ✅ Included | ✅ Included | Large-scale case-control study with full biomarker data (mean, SD, n), matched design, and OR (95% CI) for multiple exposures. Meets inclusion criteria for both qualitative and quantitative synthesis. |
| 5 | Bičíková et al., 2019, Czech Republic | Observational (Case-Control) | ✅ Included | ✅ Included | Included due to full reporting of mean 25(OH)D levels and standard deviations; estimated OR (95% CI) derived from narrative prevalence data. Matched control group and seasonal adjustment improve comparability. |
| 6 | Coşkun et al., 2016, Turkey | Observational (Case-Control) | ✅ Included | ✅ Included | Large sample size, genotype and serum biomarker data reported. OR and CI available for genetic variants. Serum 25(OH)D presented with mean and SD, convertible to standardized units (nmol/L). Subgroup data allows extraction for stratified meta-analysis. |
| 7 | De Marzio et al., 2024, U.S.A. | Observational (Prospective, systems biology) | ✅ Included | ⛔ Excluded | High-quality mechanistic modeling linking maternal and child vitD to communication via serotonin pathways; no OR/CI available; not eligible for quantitative pooling. |

| No. | Authors (Year, Country) | Study Design | Systematic Review (SR) | Meta-Analysis (MA) | Support for Decision |
|---|---|---|---|---|---|
| 8 | Egorova et al., 2020, Sweden | Observational (Retrospective case-control) | ✅ Included | ✅ Included | Study reports ASD outcome with quantifiable biomarker exposures including folate and vitamin D; Effect estimates provided (OR, CI, p-values); appropriate comparison group (neurotypical controls); sufficient data to compute effect sizes. |
| 9 | Eshawi et al. (2024, Libya) | Observational (Case-Control) | ✅ Included | ✅ Included | Extractable biomarker data (means/SDs) and estimated ORs for folate and homocysteine; B12 not significant. |
| 10 | Hendren et al., 2016, U.S.A. | Randomized Controlled Trial (RCT) | ✅ Included | ✅ Included | Placebo-controlled RCT with well-defined methyl B12 intervention; CGI-I and biomarker outcomes reported; Responder data used to estimate OR with CI and p-value; valid outcome measures and subgroup stratification |
| 11 | Javadfar et al., 2020, Iran | Randomized Controlled Trial (RCT) | ✅ Included | ✅ Included | Placebo-controlled RCT with standardized outcome measures (CARS, ATEC), reported biomarker data (Vitamin D), and complete pre-post values; OR estimated from response threshold; meets all criteria for inclusion. |
| 12 | Jayanath et al., 2021, Malaysia | Observational (Cross-sectional) | ✅ Included | ⛔ Excluded | Included in SR for insights on vitamin D deficiency and subgroup analysis; excluded from meta-analysis due to absence of control group and lack of responder-level effect size (OR/SMD) on ASD symptoms. |
| 13 | Kerley et al., 2017, Ireland | Randomized Controlled Trial (RCT) | ✅ Included | ✅ Included | Placebo-controlled RCT reporting pre-post 25(OH)D levels and standardized ASD outcomes (ADOS-2, ABAS-II, SRS); although underpowered, estimates could be included in subgroup meta-analysis with caution. OR estimated. |
| 14 | Li et al., 2024, China | Observational (Case-Control) | ✅ Included | ✅ Included | Large observational study comparing serum B12, folate, and homocysteine between ASD and TD groups; ORs for biomarkers estimated; suitable for pooled analysis in meta-analysis on postnatal biomarker status. |
| 15 | Li et al., 2022, China | Observational (Case-Control) | ✅ Included | ✅ Included | Case-control design with clear biomarker exposure quartiles; reported ORs for folate and homocysteine with full CI and estimable p-values. Suitable for quantitative synthesis. |

| No. | Authors (Year, Country) | Study Design | Systematic Review (SR) | Meta-Analysis (MA) | Support for Decision |
|---|---|---|---|---|---|
| 16 | Mazahery et al., 2019, New Zealand | Randomized Controlled Trial (RCT) | ✅ Included | ✅ Included | Placebo-controlled 4-arm RCT testing Vitamin D and Omega-3; reported mean changes and SDs for ASD core symptom scale (SRS); OR estimated for >10-point SRS improvement; suitable for subgroup synthesis in meta-analysis. |
| 17 | Mazahery et al., 2020, New Zealand | Randomized Controlled Trial (RCT) | ✅ Included | ✅ Included | Placebo-controlled 4-arm RCT including postnatal Vitamin D and Omega-3 supplementation; validated outcome (SRS); reported mean effects, p-values, and estimated OR for clinical response. Suitable for subgroup meta-analysis on inflammatory status. |
| 18 | Moradi et al., 2018, Iran | Randomized Controlled Trial (RCT) | ✅ Included | ✅ Included | Four-arm RCT evaluating vitamin D supplementation and/or motor training in ASD. Outcomes reported using validated tools (GARS-2); intervention effects quantifiable with calculated ORs. Sufficient data for effect synthesis and subgroup analysis. |
| 19 | Nesa et al., 2022, Bangladesh | Observational (Case-Control) | ✅ Included | ✅ Included | Comparative case-control study with clear ASD and control group; serum biomarkers measured using standard lab methods; effect estimates reported and/or estimable; suitable for pooled analysis of biomarker levels. |
| 20 | Petruzzelli et al., 2020, Italy | Observational (Case-Control) | ✅ Included | ✅ Included | Case-control study comparing serum 25(OH)D levels in ASD vs non-ASD neuropsychiatric controls. Reported means, SDs, and OR for deficiency; all extractable and suitable for meta-analytic integration. |
| 21 | Raghavan et al., 2017, U.S.A. | Observational (Prospective Cohort) | ✅ Included | ⛔ Excluded | Included in SR for high-quality prospective cohort design and biomarker data; excluded from meta-analysis due to use of HR instead of OR/SMD and unique outcome modeling. |
| 22 | Saad et al., 2015, Egypt | Observational (Case-Control Study & Open-Label Interventional Trial) | ✅ Included | ✅ Included | Included due to direct measurement of Vitamin D before and after supplementation, ASD diagnosis confirmed, and standardized tools used for symptom tracking (CARS, ABC). |

| No. | Authors (Year, Country) | Study Design | Systematic Review (SR) | Meta-Analysis (MA) | Support for Decision |
|---|---|---|---|---|---|
| 23 | Saas et al. (2020, Denmark) | Randomized Controlled Trial (RCT) | ✅ Included | ✅ Included | RCT with biomarker-based prenatal vitamin D intervention; reports standardized outcome metrics; includes sufficient sample size and extractable effect data. |
| 24 | Sandboge et al., 2023, Finland | Randomized Controlled Trial (RCT) | ✅ Included | ✅ Included | High-quality RCT, biomarker-based vitamin D intervention from infancy; ASD-relevant behavioral outcomes; extractable ORs and standardized dosing protocol. |
| 25 | Schmidt et al., 2019, U.S.A. | Observational (Prospective Cohort) | ✅ Included | ✅ Included | Included due to well-defined exposure (maternal biomarkers), validated ASD diagnosis, extractable group means, SDs, and ORs |
| 26 | Shom et al., 2024, India | Observational (Case-control & Cross-sectional) | ✅ Included | ✅ Included | Included due to detailed gene–nutrient interaction findings, biomarker comparisons, standardized outcome tools, and extractable ORs. |
| 27 | Sourander et al., 2023, Finland | Observational (Nested Case-Control) | ✅ Included | ✅ Included | Meets inclusion criteria for biomarker exposure and ASD outcome; extractable OR, CI, and maternal B12 stratified comparisons. |
| 28 | Tuovinen et al., 2021, Finland | Randomized Controlled Trial (RCT) | ✅ Included | ✅ Included | Meets all inclusion criteria: postnatal vitamin D intervention; validated biomarker and outcomes; extractable effect size (OR, CI) for meta-analysis. |
| 29 | Vinkhuyzen et al., 2017, Netherlands | Observational (Nested Case-Control) | ✅ Included | ✅ Included | Mid-gestation vitamin D and ASD diagnosis both quantified and extractable; eligible for stratified meta-analysis of biomarker levels. |
| 30 | Vinkhuyzen et al., 2018, Netherlands | Observational (Cohort) | ✅ Included | ✅ Included | Included due to extractable adjusted ORs for B12, folate, and interaction term; high-quality exposure and ASD diagnostic data |
| 31 | Windham et al., 2020, U.S.A. | Observational (Case-Control) | ✅ Included | ✅ Included | Reported adjusted ORs stratified by ASD and ID subtypes; vitamin D in standardized nmol/L; eligible for inclusion in meta-analysis. |
| 32 | Wink et al., 2016, U.S.A. | Randomized Controlled Trial (RCT) | ✅ Included | ✅ Included | Standardized biomarker (GSH, Homocysteine) outcomes and CGI-I data support inclusion in meta-analysis. |

| No. | Authors (Year, Country) | Study Design | Systematic Review (SR) | Meta-Analysis (MA) | Support for Decision |
|---|---|---|---|---|---|
| 33 | Wu et al. (2018, China) | Observational (Nested Case-Control) | ✅ Included | ✅ Included | Clear ORs and CI; high-quality biomarker data (25(OH)D3 from DBS); large sample; supports inclusion in both synthesis arms |
| 34 | Yektaş et al. (2019, Turkey) | Observational (Cross-Sectional Case-Control) | ✅ Included | ✅ Included | Reported means/IQRs for biomarkers, stratified groups (ASD, ADHD, Control), estimated ORs available for all biomarkers. |
| 35 | Zou et al. (2024, China) | Observational (Case-Control & Cross-Sectional) | ✅ Included | ✅ Included | Fully reported biomarker outcomes with OR, CI, and p-values; valid for quantitative synthesis. |

**Notes:**

- **ABAS-II:** Adaptive Behavior Assessment System, Second Edition.
- **ADOS-2**: Autism Diagnostic Observation Schedule, Second Edition.
- **ATEC**: Autism Treatment Evaluation Checklist.
- **CARS**: Childhood Autism Rating Scale.
- **CGI-I**: Clinical Global Impression – Improvement Scale.
- **ELISA**: Enzyme-Linked Immunosorbent Assay.
- **TD**: Typically Developing children.

**Citation:** All references are cited in the main text.

**Table S4**. List of Excluded Studies from Qualitative and/or Quantitative Synthesis with Justification Based on Predefined Eligibility Criteria.

| No. | Authors (Year), Country | Study Design | Eligibility Decision | Potential Use in Narrative Sections | Support for Decision |
|---|---|---|---|---|---|
| **1** | Azzini et al. (2020), Italy | Narrative/Systematic Review (non-meta-analytic) | ❌ Excluded | Provides comprehensive background on homocysteine metabolism and its role across the lifespan, including in ASD—valuable for discussion and context. | No PRISMA checklist or structured methodology. No risk of bias assessment, narrative synthesis only. Lack of statistical synthesis. |
| **2** | Adibsaber et al. (2024), Iran | Systematic Review/Meta-analysis | ❌ Excluded | Comparative background. | Secondary synthesis only. No original data. Excluded from Qualitative and/or Quantitative Synthesis inclusion but cited in comparison framework. |
| **3** | Brîndușe et al. (2024), Romania | Narrative Review | ❌ Excluded | Introduction context. | Conceptual review. Lacks empirical biomarker data. Excluded for not meeting PICO. |
| **4** | Bonetti et al. (2016), Italy | Narrative Review | ❌ Excluded | Background and Theoretical Framing | Does not follow PRISMA methodology, lacks search strategy, inclusion/exclusion criteria, and structured critical appraisal. No quantitative synthesis (meta-analysis) is provided. The focus is on general neurodegeneration rather than ASD or developmental neurodisorders. |
| **5** | Cannell et al. (2017), U.S.A. | Narrative Review | ❌ Excluded | Comparative reference in the Discussion section | No PRISMA adherence, no risk of bias assessment, no structured data extraction. Primarily hypothesis-driven narrative with mechanistic interpretation. |
| **6** | Chiriță-Emandi et al. (2015), Romania | Observational (Cross-sectional) | ❌ Excluded | Population-level vitamin D context. | No Autism Spectrum Disorder (ASD) outcome analyzed. Vitamin D prevalence only. No relevance to ASD association. |
| **7** | Dekkers et al. (2015), Ecuador | Observational (Epigenetic Cohort) | ❌ Excluded | Methylation mechanisms in ASD. | Focused on DNA (Deoxyribonucleic acid) methylation without nutritional biomarkers (Vitamin D/B12/Homocysteine). Outside scope. |

| No. | Authors (Year), Country | Study Design | Eligibility Decision | Potential Use in Narrative Sections | Support for Decision |
|---|---|---|---|---|---|
| **8** | Dias et al. (2020), U.S.A. | Narrative Review | ❌Excluded | Background source for genetic overlap and pathways. | Focused exclusively on genetic architecture, no mention of vitamin D, B12, Homocysteine, or related nutritional markers. Not aligned — Lacks interventional or comparative structure. No nutritional exposure or outcome variables that could be mapped to our PICO criteria. |
| **9** | Doi et al. (2024), Japan | Observational (Genome-Wide Association /Immune Genetics Study) | ❌Excluded | Immune-genetic pathways. | Immune gene pathways; lacks nutritional biomarker focus. Not relevant to nutrient-ASD linkage. |
| **10** | Endres et al. (2016), Germany | Observational (Case-Control) | ❌Excluded | Comparative value for adult ASD biomarker studies | Adult-only sample. Outside critical exposure window (prenatal/early childhood). |
| **11** | Gao et al. (2024), China | Observational (Case-Control) | ❌Excluded | Referenced in the Discussion | Branched-Chain Amino Acids (BCAAs) – not Vitamin D, B12, or Homocysteine. Outside of predefined biomarkers. |
| **12** | Gerges et al. (2024), Lebanon | Observational (Microbiota Study) | ❌Excluded | Microbiome–neurodevelopment axis. | No Vitamin D/B12/Homocysteine measured. Entirely microbiome-focused. |
| **13** | Ghiga et al. (2024), Romania | Observational (Cross-sectional) | ❌Excluded | National prevalence for vitamin D. | Vitamin D levels assessed but no ASD diagnosis. General pediatric data only. |
| **14** | Grant et al. (2019), U.S.A. | Narrative Review | ❌Excluded | Comparative reference in the discussion section—supports contextual understanding of vitamin D's broad impact and ASD relevance. | No PRISMA framework or risk of bias assessment; broad narrative approach based on literature synthesis from observational data. Lacks pooled effect sizes or extractable quantitative estimates. |
| **15** | Guerini et al. (2024), Italy. | Observational (Immunological Subtyping) | ❌Excluded | Immunogenetic background context. | Human Leukocyte Antigen (HLA) and immune marker focus. Lacks nutritional biomarkers. |
| **16** | Herdea et al. (2024), Romania | Observational (Prevalence Study) | ❌Excluded | Deficiency background in pediatrics. | Descriptive vitamin D data. No ASD outcome. |

| No. | Authors (Year), Country | Study Design | Eligibility Decision | Potential Use in Narrative Sections | Support for Decision |
|---|---|---|---|---|---|
| **17** | Hewitson et al. (2024), U.S.A. | Observational (Proteomics Study) | ❌Excluded | Future biomarker pathway hypotheses. | Protein markers only. No Vitamin D/B12/Homocysteine measured. Outside scope. |
| **18** | Keehn et al. (2024), U.S.A. | Observational (Eye-tracking/Behavioral Study) | ❌Excluded | Biomarker diversity commentary. | Behavioral biomarker only. No biochemical measurement. |
| **19** | Kong et al. (2019), China | Observational (Microbiota Study) | ❌Excluded | Gut-brain-nutrition background. | No nutritional biomarkers. Focused on gut–brain axis only. |
| **20** | Krakowiak et al. (2017), USA | Observational (Immune Cytokine Study) | ❌Excluded | Immune models in ASD etiology. | Focused on cytokines. Lacks Vitamin D/B12/Homocysteine. |
| **21** | Li et al. (2017), China | Systematic Review (non-meta-analytic) | ❌Excluded | Contextual narrative reference only. | Not aligned – no exposure to nutritional biomarkers (Population: ASD children, but Intervention/Exposure: neuroimaging only). |
| **22** | Manghi et al. (2024), Italy | Observational (Oral Microbiome) | ❌Excluded | Alternative microbiota context. | No nutritional biomarkers. Focused on microbial enzymes. |
| **23** | Mogire et al. (2021), Kenya | Observational (Population Survey) | ❌Excluded | Vitamin D deficiency prevalence. | Vitamin D prevalence data only. No ASD-related outcomes. |
| **24** | Myat et al. (2025), Australia | Observational (Sociodemographic Cross-sectional) | ❌Excluded | Environmental risk context. | No biomarker data included. Focus on perinatal risk factors. |
| **25** | Niculescu et al. (2017), Romania | Observational (Cross-Sectional with Retrospective Data Extraction) | ❌Excluded | Contextual background reference only. | No mention of ASD, neurodevelopmental disorders, or cognitive/behavioral outcomes. Not aligned: Lacks outcome related to ASD; while vitamin D is measured, there is no target population with ASD and no interventional/comparative component relevant to neurodevelopment. |
| **26** | Noori et al. (2024), Iran | Observational (Inflammatory Biomarkers) | ❌Excluded | Immuno-inflammatory ASD mechanisms. | Only cytokines measured. Lacks nutritional biomarkers. |

| No. | Authors (Year), Country | Study Design | Eligibility Decision | Potential Use in Narrative Sections | Support for Decision |
|---|---|---|---|---|---|
| **27** | Peralta-Marzal et al. (2021), Netherlands | Systematic Review | ❌ Excluded | Comparative reference for contextual framing of the gut-brain-microbiota link in ASD. | Gut microbiota-derived metabolites, primarily SCFAs, (Short-Chain Fatty Acids) aromatic amino acids, kynurenine derivatives, indolic compounds. |
| **28** | Ramirez-Celis et al. (2022), U.S.A | Observational (Immune Autoantibodies) | ❌ Excluded | Maternal immune activation models. | Autoimmune markers. No vitamin D, B12, or Homocysteine. |
| **29** | Ranjan et al. (2015), U.S.A. | Narrative Review | ❌ Excluded | Comparative reference in the Discussion section. | No formal PRISMA adherence; narrative synthesis of studies grouped by anthropometric, biochemical, and dietary dimensions; search strategy is described, but lacks structured bias assessment. Does not provide extractable effect sizes. |
| **30** | Russell-Jones et al. (2022), Australia | Observational (B2 Metabolism Study) | ❌ Excluded | One-carbon metabolism discussion. | Vitamin B2 only. No vitamin D, B12 or Homocysteine measured. |
| **31** | Sharma et al. (2015), U.S.A. | Narrative MiniReview | ❌ Excluded | Valuable background source on the biological and mechanistic implications of homocysteine in ASD and other neurodevelopmental and neurodegenerative conditions. | No PRISMA adherence, no risk of bias assessment, no structured database search. Purely narrative synthesis. Lacks pooled data or statistical synthesis. Discusses findings from various studies but provides no effect sizes or unified summary statistics for comparison with our primary dataset. |
| **32** | Soares et al. (2024), Brazil | Observational (Nutritional: Zinc/Calcium) | ❌ Excluded | Food rigidity, diet patterns. | Non-target nutrients. No D/B12/ Homocysteine biomarkers reported. |
| **33** | Sultan et al. (2025), Saudi Arabia | Narrative Review | ❌ Excluded | Comparative narrative reference. | No PRISMA adherence; narrative synthesis; includes tabled evidence and summarization of observational studies, RCTs, and meta-analyses, but lacks protocol registration and structured methodology. |
| **34** | Tran et al. (2024), Vietnam | Observational (Epidemiological Survey) | ❌ Excluded | Epidemiological framing. | No biomarker data. Prevalence study only. |

| No. | Authors (Year), Country | Study Design | Eligibility Decision | Potential Use in Narrative Sections | Support for Decision |
|---|---|---|---|---|---|
| **35** | Ye et al., (2025), China | Systematic Review and Meta-analysis of Mendelian Randomization Studies | ❌ Excluded | Core comparative reference in both the Discussion and Introduction sections. | Study Design. |
| **36** | Zhang et al. (2018), China | Observational (VDR Genetic Study) | ❌ Excluded | Genotype–environment interaction section. | Focused on polymorphisms. No serum vitamin D levels. |
| **37** | Zhang et al. (2024), China | Observational (Vitamin D prevalence) | ❌ Excluded | Regional Vitamin D baseline | No ASD outcomes measured. General population data only. |
| **38** | Zhao et al. (2023), China | Study Protocol | ❌ Excluded | Future research direction | No results available. Ongoing study. Design only. |

**Citation:** All references are cited in the main text.

**Table S5.** Summary of Included Studies and Synthesis of Key Characteristics, Organized by Type of Intervention.

| No. | Authors | Study | Population | Intervention | Comparison | Outcome | Biomarker | Data Interpretation |
|---|---|---|---|---|---|---|---|---|
| **INTERVENTION TYPE 1: Prenatal Nutrient Exposure** Studies that measure maternal plasma or serum concentrations of vitamin D, B12, or homocysteine during gestation (typically spanning the first to third trimester), intended to capture fetal exposure during critical stages of brain development. | | | | | | | | |
| **1** | Aagaard et al. (2024, Denmark) | Randomized Controlled Trial (RCT) | 700 total (496 analyzed; 246 high-dose, 250 standard dose). | Prenatal Nutrient Exposure — High-dose vs. standard-dose prenatal vitamin D3 (2800 IU vs. 400 IU daily from week 24 gestation to 1 week postpartum). | Randomized arms: high-dose vs. standard-dose. | Autism at 10y: 5/246 (2.0%) high-dose vs. 7/250 (2.8%) standard dose; ADHD: 27 (11%) vs. 31 (12.4%); Autistic symptom load: 15 (6.1%) vs. 25 (10%); ADHD symptom load: 67 (27.2%) vs. 76 (30.4%); Maternal 25(OH)D preintervention per 10 nmol/L: OR autism = 0.76 (CI: 0.59–0.97, p = 0.034); β autistic symptoms = –0.03 (p = 0.024); OR ADHD = 0.88 (CI: 0.78–0.99, p = 0.033). | Vitamin D (25(OH)D). | No effect of high-dose intervention overall; higher maternal preintervention vitamin D associated with lower autism and ADHD risk and autistic symptoms; possible protective effect in those with ≥75 nmol/L preintervention levels; U-shaped threshold effect suggested. |
| **2** | Egorova et al. (2020, Sweden) | Observational (Retrospective case-control) | 200 total (100 ASD cases, 100 controls). | Prenatal Nutrient Exposure - Serum folate and other biomarkers at ~14 weeks gestation. | ASD vs. typically developing controls. | Folate (nmol/L): OR per 1 SD increase = 1.70 (95% CI: 1.22–2.37), p = 0.002; Vitamin D (nmol/L): OR per 1 SD = 0.78 (95% CI: 0.58–1.08), p = 0.11; CRP (mg/L): OR per 1 SD = 1.20 (95% CI: 0.89–1.61), p = 0.24; KTR (unitless): OR = 0.91 (95% CI: 0.66–1.26), p = 0.58; Neopterin (nmol/L): OR per 1 SD = 0.97 (95% CI: 0.72–1.31), p = 0.86. | Folate, Vitamin D, CRP, KTR, Neopterin. | Higher maternal serum folate (nmol/L) at ~14 weeks associated with increased ASD risk in offspring. No significant associations found for vitamin D, CRP, or immune markers. All ORs are expressed per 1 SD change in biomarker concentration, in units standardized to meta-analysis conventions. |

| No. | Authors | Study | Population | Intervention | Comparison | Outcome | Biomarker | Data Interpretation |
|---|---|---|---|---|---|---|---|---|
| **3.** | Saas et al. (2020, Denmark) | Randomized Controlled Trial (RCT) | 623 total (551 analyzed: 277 high-dose, 274 standard-dose). | Prenatal Nutrient Intervention — High-dose vs. standard-dose Vitamin D3 (2800 IU vs. 400 IU daily, week 24 to 1 week postnatal). | Randomized arms: high-dose (2800 IU) vs. standard dose (400 IU); stratified by sex; co-intervention: n-3 LCPUFA (both groups); subgroup without n-3 included. | Motor milestones: $\beta = 0.08$ (95% CI: –0.26 to 0.43), p = 0.64; Cognitive development (Bayley-III at 2.5y): $\beta = 0.34$ (95% CI: –1.32 to 1.99), p = 0.70; Language at 2y: median 232 (113–346) vs. 253 (149–382.5), p = 0.02; Emotional/behavioral SDQ: OR = 0.76 (95% CI: 0.53–1.09), p = 0.14; Vitamin D levels at birth: 43.18 ± 14.13 vs. 28.94 ± 12.47 ng/mL → Converted: 107.95 vs. 72.35 nmol/L. | Vitamin D (25(OH)D). | No consistent effect of high-dose supplementation on neurodevelopment; isolated lower word production at age 2 in high-dose group; co-supplementation and sex interaction considered. |
| **4** | Schmidt et al. (2019, USA) | Observational (Prospective Cohort) | Mother-child pairs from the MARBLES cohort; ASD = 46, Non-TD = 55, TD = 62. | Prenatal Nutrient Exposure — Maternal blood samples collected during pregnancy; analysis of one-carbon metabolites. | ASD vs. TD vs. Non-TD; Tertiles of biomarker concentrations. | B12: ASD = 424.0 ± 122.5 pg/mL, TD = 466.8 ± 138.5 pg/mL, p = 0.049 → Converted: 313.3 vs. 344.6 pmol/L; Folate: 13.5 ± 4.1 vs. 14.9 ± 4.3 ng/mL, p = 0.09 → Converted: 30.7 vs. 33.8 nmol/L; Homocysteine: 5.47 ± 1.32 vs. 5.19 ± 1.49 μmol/L, p = 0.34; OR for lowest $B_{12}$ tertile vs. highest = 2.25 (95% CI: 1.01–5.00), p = 0.047. | Vitamin $B_{12}$ (pg/mL → pmol/L); Folate (ng/mL → nmol/L); Homocysteine (μmol/L). | Lower maternal $B^{12}$ during mid-pregnancy associated with increased ASD risk; Folate and homocysteine not statistically significant but show suggestive trends. |
| **5** | Sourander et al. (2023, Finland) | Observational (Nested Case-Control) | 3116 total (1558 ASD cases, 1558 matched controls). | Prenatal Nutrient Exposure — Maternal vitamin $B_{12}$ concentration measured during early gestation (mean gestational week ~10). | $B_{12}$ exposure in quintiles; matched by DOB, sex, place of birth. | High $B_{12}$ (≥81st percentile): OR = 1.59 (95% CI: 1.06–2.41), p = 0.026<br>Low B12 (<20th percentile): OR = 1.49 (95% CI: 0.98–2.26), p = 0.064<br>Log-B12: OR = 0.90 (95% CI: 0.77–1.06), p = 0.209 (unadj.), OR | Vitamin $B_{12}$ (pmol/L) — standardized unit. | High maternal vitamin $B_{12}$ during early pregnancy (≥165 pmol/L) associated with increased odds of childhood autism. U-shaped association suggested. No effect found on overall |

| No. | Authors | Study | Population | Intervention | Comparison | Outcome | Biomarker | Data Interpretation |
|---|---|---|---|---|---|---|---|---|
| | | | | | | = 0.94 (95% CI: 0.79–1.10), p = 0.441 (adj.). | | ASD, Asperger's, or PDD-NOS subtypes. |
| **6** | Vinkhuyzen et al. (2017, Netherlands). | Observational (Nested Case-Control) | 4334 children with mid-gestation or neonatal serum 25(OH)D data; 68 ASD cases. | Prenatal Nutrient Exposure / Neonatal Nutrient Biomarkers — Maternal and cord serum 25(OH)D levels assessed via LC-MS/MS at mid-gestation (~20.6 weeks) and at birth. | 25(OH)D Deficient (<25 nmol/L) vs. Sufficient (≥50 nmol/L) at mid-gestation and at birth. | Mid-gestation 25(OH)D deficiency associated with increased ASD risk: OR = 2.42 (95% CI: 1.09–5.07), p = 0.03; Cord blood 25(OH)D deficiency: OR = 0.94 (95% CI: 0.36–2.42), p = 0.90; Insufficiency (25–49.9 nmol/L): mid-gestation OR = 0.86 (CI: 0.43–1.62), p = 0.64; cord OR = 1.25 (CI: 0.61–2.69), p = 0.54. | Vitamin D (25(OH)D). | Mid-gestational vitamin D deficiency (<25 nmol/L) significantly associated with increased ASD risk; No significant association with cord blood vitamin D status; results robust across sensitivity analyses. |
| **7** | Vinkhuyzen et al. (2018, Netherlands). | Observational (Cohort) | 4,229 children (ABCD cohort), 68 ASD cases. | Prenatal Nutrient Exposure — Maternal serum vitamin $B_{12}$ and folate levels during early pregnancy. | ASD vs. neurotypical children (from clinical ASD diagnosis using national registers); stratified analyses for high vs. low vitamin $B_{12}$ and folate levels. | Vitamin $B_{12}$: OR = 1.16 (95% CI: 0.90–1.49), p = 0.25<br>Folate: OR = 1.17 (95% CI: 0.93–1.49), p = 0.17<br>$B_{12}$ × Folate interaction: OR = 1.26 (95% CI: 0.97–1.63), p = 0.08. | Vitamin $B_{12}$, Folate. | No significant associations for individual $B_{12}$ or folate; suggestive interaction effect (high $B_{12}$ + folate) possibly increasing ASD risk. |
| **8** | Windham et al. (2020, U.S.A.) | Observational (Case-Control) | 929 total (474 ASD, 154 ID only, 301 controls); maternal serum at 15–19 weeks | Prenatal Nutrient Exposure — Maternal 25(OH)D measured in mid- | ASD vs. controls; ASD with ID vs. controls; ASD without ID vs. controls; ID only vs. controls. | Vitamin D (25(OH)D) – Geometric Mean (nmol/L): ASD = 84.3; ID = 82.5; Control = 83.8<br>Deficiency (<50 nmol/L)<br>ASD: OR = 0.79 (95% CI: 0.49–1.30), p > 0.05. ASD + ID: OR = | Vitamin D (25(OH)D). | No statistically significant associations for maternal vitamin D status and ASD/ID outcomes; all results adjusted. Non-linear trends suggested |

| No. | Authors | Study | Population | Intervention | Comparison | Outcome | Biomarker | Data Interpretation |
|---|---|---|---|---|---|---|---|---|
| | | | | gestation (nmol/L). | | 0.95 (0.54–1.67), p > 0.05. ASD – ID: OR = 0.67 (0.34–1.33), p > 0.05. ID only: OR = 1.15 (0.60–2.19), p > 0.05. Insufficiency (50–74 nmol/L): ASD: OR = 0.93 (0.68–1.28), p > 0.05. ASD + ID: OR = 1.10 (0.76–1.58), p > 0.05. ASD – ID: OR = 0.82 (0.54–1.25), p > 0.05. ID only: OR = 0.75 (0.47–1.12), p > 0.05. Per 25 nmol/L increase ASD: OR = 0.95 (0.86–1.05), p > 0.05. ASD + ID: OR = 0.92 (0.82–1.04), p > 0.05. ASD – ID: OR = 0.98 (0.86–1.11), p > 0.05. ID only: OR = 1.01 (0.88–1.16), p > 0.05. | | via spline models but not statistically conclusive. |
| **INTERVENTION TYPE 2: Neonatal Nutrient Biomarkers** Studies assessing immediate postnatal biomarker levels—often via cord blood or neonatal dried blood spots—as retrospective proxies for intrauterine nutritional status. | | | | | | | | |
| **9** | Raghavan et al. (2017, USA) | Observational (Prospective Cohort) | 1257 mother-child pairs (ASD = 86, Neurotypical = 1171). | Neonatal Nutrient Biomarkers + Prenatal Nutrient Exposure — Maternal multivitamin intake during pregnancy; neonatal serum folate, $B_{12}$, homocysteine measured at birth. | ASD vs. neurotypical; multivitamin use: ≤2x/week, 3–5x/week (ref), >5x/week; folate/$B_{12}$/homocysteine percentile groups. | HR ≤2x/week = 3.5 (95% CI: 1.7–7.4), p<0.001; >5x/week = 2.1 (95% CI: 1.2–3.6), p<0.01; Folate ≥90th = HR = 2.5; $B_{12}$ ≥90th = HR = 2.5; Combined high folate + B12 = HR = 13.7 | Estimated ORs: Vitamin $B_{12}$ (≥536.8 pmol/L): OR = 2.22 (95% CI: 1.26–3.90), p = 0.010; Folate (≥60.3 nmol/L): OR = 2.22 (95% CI: 1.26–3.90), p = 0.010; Homocysteine (high levels): OR = 0.92 (95% CI: | U-shaped risk for multivitamin intake; high folate and $B_{12}$ associated with ASD risk; homocysteine not significant |

| No. | Authors | Study | Population | Intervention | Comparison | Outcome | Biomarker | Data Interpretation |
|---|---|---|---|---|---|---|---|---|
| | | | | | | | 0.44–1.96), p = 1.000 | |
| **10** | Wu et al. (2018, China) | Observational (Nested Case-Control) | 1550 total (310 ASD, 1240 controls). | Neonatal Nutrient Biomarker — 25(OH)D3 levels from dried blood spots (DBS) collected 24–48h after birth | ASD vs. controls; quartiles & quintiles; subgroup analysis for ASD with ID. | Median 25(OH)D3: ASD = 17.6 nmol/L [IQR: 12.4–27.4] vs. Control = 40.2 nmol/L [IQR: 27.7–48.8], p<0.0001. Vitamin D Deficiency (<30 nmol/L): ASD = 77.1%, Control = 28.5%; OR = 8.46 (95% CI: 6.32–11.33), p<0.001. Multivariate adjusted OR = 3.68 (95% CI: 2.03–5.24), p<0.001. Quartiles vs. Q4: Q1 = OR 3.6 (1.8–7.2), Q2 = 2.5 (1.4–3.5), Q3 = 1.9 (1.1–3.3). ID group: 25(OH)D3 = 17.3 nmol/L vs. Control = 40.5 nmol/L, p<0.0001. | Vitamin D (25(OH)D3, nmol/L). | Strong association between low neonatal 25(OH)D3 and ASD/ID risk; clear dose-response effect; robust results. |
| **INTERVENTION TYPE 3: Postnatal/Early Childhood Nutrient Status** Studies examining vitamin and homocysteine levels in toddlers and young children (generally up to age 6), who are either diagnosed with ASD or considered at elevated risk. | | | | | | | | |
| **11** | Altun et al. (2018, Turkey) | Observational (Case-Control) | 105 total (60 ASD, 45 controls); Age: 3–12 years; Sex: ASD 52M/8F, Control 36M/9F | Primary: Postnatal/Early Childhood Nutrient Status; Secondary: Gene–Nutrient Interaction Studies — Serum levels of vitamin D, VDR, B6, $B_{12}$, folate, and homocysteine | ASD vs. neurotypical control group, matched by age, sex, and season of blood collection. | Vitamin D (25(OH)D): 13.79 ± 1.03 ng/mL (ASD) vs. 16.58 ± 1.06 ng/mL (Control), p < 0.001; Converted: 34.48 vs. 41.45 nmol/L; Estimated OR = 1.00 (95% CI: 0.06–16.76), p ≈ 1.000. Vitamin D Receptor (VDR): 1.24 ± 0.11 vs. 1.92 ± 0.26, p < 0.001 (unitless); Homocysteine: 8.90 ± 0.19 µmol/L vs. 7.46 ± 0.21 µmol/L, p < 0.001; | Vitamin D, Vitamin D Receptor (VDR), Homocysteine, Vitamin B6, $B_{12}$, Folate. | Children with ASD had significantly lower levels of vitamin D, VDR, B6, B12, and folate, and higher homocysteine levels compared to controls. Strong correlations with ASD severity (CARS). First study to assess serum VDR levels in ASD; supports role of multiple |

| No. | Authors | Study | Population | Intervention | Comparison | Outcome | Biomarker | Data Interpretation |
|---|---|---|---|---|---|---|---|---|
| | | | | measured in children aged 3–12. | | Estimated OR = 0.97 (95% CI: 0.06–16.19), p ≈ 0.981. Vitamin B6: 25.17 ± 3.64 vs. 53.06 ± 7.95, p < 0.001; (units not specified); Folate: 121.16 ± 8.04 vs. 172.31 ± 17.19 pg/mL, p < 0.001; Converted: 89.44 vs. 131.26 nmol/L (using 1 ng/mL = 2.27 nmol/L → 1 pg/mL = 0.00227 nmol/L); Vitamin $B_{12}$: 181.5 ± 41.61 pg/mL vs. 382.06 ± 71.34 pg/mL, p < 0.001; Converted: 134.00 vs. 282.00 pmol/L; Estimated OR = 60.00 (95% CI: 7.12–505.94), p ≈ 0.0002. CARS (Childhood Autism Rating Scale) score: 42.3 ± 7.46 (ASD) vs. 15.9 ± 1.02 (Control), p < 0.001. Correlations: Vitamins (B6, Folate, $B_{12}$, D): r = −0.687 to −0.840 with CARS score, p < 0.001. Homocysteine: r = +0.688 with CARS score, p < 0.001. | | biochemical disruptions in ASD etiopathogenesis. |
| **12** | Arastoo et al. (2018, Iran) | Observational (Case-Control) | 62 total (31 ASD, 31 controls); Age: 5–12 years; Sex: ASD 26M/5F, Control 28M/3F. | Postnatal/Early Childhood Nutrient Status — Serum 25(OH)D levels measured via ELISA in fasted morning samples. | ASD vs. neurotypical control group; matched by age, sex, food intake, socioeconomic status, sun exposure, residence. | Vitamin D (25(OH)D): 9.03 ± 4.14 ng/mL (ASD, n = 31) vs. 15.25 ± 7.89 ng/mL (Control, n = 31), p < 0.001; Converted: 22.58 ± 10.35 nmol/L vs. 38.13 ± 19.72 nmol/L. Deficiency defined as ≤20 ng/mL (= 50 nmol/L): ASD = 30/31 (96.8%), Control = 22/31 | Vitamin D (25(OH)D) | Children with ASD had significantly lower serum vitamin D levels than controls. OR = 12.27 for vitamin D deficiency indicates a strong association with ASD risk. Study did not assess other nutritional |

| No. | Authors | Study | Population | Intervention | Comparison | Outcome | Biomarker | Data Interpretation |
|---|---|---|---|---|---|---|---|---|
| | | | | | | (70.97%). Estimated OR = 12.27 (95% CI: 1.45–103.9), p ≈ 0.021. Insufficiency (21–29 ng/mL): ASD = 1/31 (3.2%), Control = 9/31 (29.03%). OR not calculated due to small cell frequency and asymmetry. | | biomarkers; results support hypothesis linking vitamin D status and ASD. |
| **13** | Bener et al. (2017, Qatar) | Observational (Case-Control) | 616 total (308 ASD, 308 controls); Age: <8 years; Sex: ASD 153M/155F, Control 137M/171F. | Postnatal/Early Childhood Nutrient Status — Serum measurements of Vitamin D, Iron, Ferritin, Calcium; blood samples analyzed via RIA and hematology analyzer. | ASD vs. age- and sex-matched neurotypical controls; adjusted in logistic regression for BMI, sun exposure, consanguinity. | Vitamin D (25(OH)D): 18.79 ± 8.35 ng/mL (ASD, n = 308) vs. 22.18 ± 9.00 ng/mL (Control, n = 308), p = 0.004; Converted: 46.98 ± 20.88 nmol/L vs. 55.45 ± 22.50 nmol/L. OR for vitamin D deficiency: 2.36 (95% CI: 1.74–3.44), p = 0.002. Serum Iron: 74.13 ± 21.61 µg/dL (ASD) vs. 87.59 ± 23.36 µg/dL (Control), p = 0.003; OR for iron deficiency: 2.83 (95% CI: 1.81–4.72), p < 0.001. Ferritin: 36.57 ± 5.12 ng/mL (ASD) vs. 38.49 ± 5.73 ng/mL (Control), p < 0.001; OR: 2.45 (95% CI: 1.86–3.93), p = 0.004. Calcium: 2.09 ± 0.12 mmol/L (ASD) vs. 2.39 ± 0.14 mmol/L (Control), p < 0.001; OR for low calcium: 2.74 (95% CI: 1.65–4.81), p < 0.001. | Vitamin D (Iron, Ferritin, Calcium). | Children with ASD had significantly lower serum levels of vitamin D, iron, ferritin, and calcium. Multivariate logistic regression confirmed each deficiency as an independent predictor of ASD. Findings support a strong association between multiple micronutrient deficiencies and ASD risk in a young Qatari population. |
| **14** | Bičíková et al. (2019, Czech Republic) | Observational (Case-Control) | 85 boys total (ASD: 45; Controls: 40); Age 4–7 years. | Postnatal/Early Childhood Nutrient Status — Serum 25(OH)D | ASD vs. neurotypical controls; matched | Vitamin D (25(OH)D): Mean serum 25(OH)D (calcidiol): 65.07 ± 25.95 nmol/L (ASD, n = 45) vs. 70.35 ± 20.73 nmol/L (Control, n | Vitamin D (25(OH)D, calcidiol). | No statistically significant difference in serum 25(OH)D levels between ASD and controls. |

| No. | Authors | Study | Population | Intervention | Comparison | Outcome | Biomarker | Data Interpretation |
|---|---|---|---|---|---|---|---|---|
| | | | | (calcidiol) levels measured using ECLIA method. | by age and season. | = 40). Median values: 65.22 nmol/L (ASD), 64.46 nmol/L (Control). Suboptimal vitamin D levels defined as <75 nmol/L: Estimated: ASD = 29/45 (65%), Control = 24/40 (60%). Estimated OR = 1.24 (95% CI: 0.48–3.22), p ≈ 0.673. | | Estimated OR = 1.24 (95% CI: 0.48–3.22) suggests minimal difference in risk of vitamin D insufficiency. Study limited by small sample and absence of exact deficiency frequencies. |
| **15** | Li et al. (2024, China) | Observational (Case-Control) | ASD: n=254; TD: n=265; Age range: 2–7 years; Male %: 84% (ASD), 83% (TD). | Postnatal/Early Childhood Nutrient Status — Blood levels of vitamin B12, folate, and homocysteine measured at baseline. | ASD vs. TD (Typically Developing) children. | Vitamin $B_{12}$: ASD = 374.4 ± 145.5 pg/mL, TD = 426.7 ± 146.3 pg/mL; $p < 0.001$ → Converted: 276.3 vs. 314.9 pmol/L. Estimated OR (<300 pg/mL): 2.03 (95% CI: 1.43–2.88), $p < 0.0001$. Folate: ASD = 6.8 ± 2.6 ng/mL, TD = 7.6 ± 3.2 ng/mL; $p < 0.01$ → Converted: 15.4 vs. 17.3 nmol/L. Estimated OR (<5 ng/mL): 1.98 (95% CI: 1.32–2.97), p = 0.0009. Homocysteine: ASD = 6.1 ± 2.1 µmol/L, TD = 5.3 ± 1.6 µmol/L; $p < 0.001$. Estimated OR (>6.5 µmol/L): 2.16 (95% CI: 1.50–3.11), $p < 0.0001$. | Vitamin $B_{12}$, Folate, Homocysteine. | ASD group had significantly lower serum levels of vitamin $B_{12}$ and folate, and higher homocysteine levels. Estimated ORs indicate higher odds of ASD in children with low $B_{12}$ (<300 pg/mL), low folate (<5 ng/mL), and elevated Hcy (>6.5 µmol/L). Findings support hypothesis of disrupted one-carbon metabolism in ASD. |
| **16** | Li et al. (2022, China). | Observational (Case-Control) | ASD: n=124; TD: n=124; Age: 3–7 years; Matched for age and sex. | Postnatal/Early Childhood Nutrient Status — Serum levels of folate and homocysteine measured at baseline. | ASD vs. TD (Typically Developing) children; comparisons by biomarker quartiles. | Homocysteine (highest vs. lowest quartile): OR = 11.769 (95% CI: 1.207–114.784), p ≈ 0.034. Folate (lowest vs. highest quartile): OR = 4.227 (95% CI: 1.022–17.488), p ≈ 0.047. | Folate, Homocysteine. | Children with ASD had significantly higher odds of elevated homocysteine and lower folate levels. Dose-response patterns support altered one-carbon metabolism hypothesis in ASD etiology. |

| No. | Authors | Study | Population | Intervention | Comparison | Outcome | Biomarker | Data Interpretation |
|---|---|---|---|---|---|---|---|---|
| **17** | Nesa et al. (2022, Bangladesh) | Observational (Case-Control) | 100 total (50 ASD children, 50 neurotypical controls), 66% male. | Postnatal/Early Childhood Nutrient Status — Serum levels of Vitamin $B_{12}$, Folate, and Homocysteine. | Children with ASD vs age- and sex-matched neurotypical controls (mean age: ASD = 5.71±2.06, Control = 6.00±1.99). | Vitamin $B_{12}$: 241.46±60.51 vs 302.58±76.66 pg/mL ($p<0.001$), Converted: 178.3 vs 223.3 pmol/L; Folate: 7.12±2.16 vs 9.72±2.96 ng/mL ($p<0.001$), Converted: 16.16 vs 22.07 nmol/L; Homocysteine: 8.08±2.11 vs 6.12±1.71 µmol/L ($p<0.001$); Estimated OR for low B12 = 4.17 (95% CI: 1.72–10.11), $p < 0.002$; OR for low folate = 4.44 (95% CI: 1.82–10.82), $p < 0.001$; OR for high homocysteine = 3.81 (95% CI: 1.65–8.81), $p < 0.002$. | Vitamin $B_{12}$, Folate, Homocysteine | Children with ASD had significantly lower serum $B_{12}$ and folate, and higher homocysteine compared to controls. Findings support impaired methylation and increased oxidative stress in ASD. |
| **18** | Petruzzelli et al. (2020, Italy) | Observational (Case-Control) | 90 total (54 ASD children, 36 children with other neuropsychiatric disorders); ASD: 81.5% male. | Postnatal/Early Childhood Nutrient Status — Serum 25(OH)D concentration. | ASD group vs. non-ASD neuropsychiatric group (including ID, ADHD, learning/language /motor disorders, epilepsy, psychiatric disorders). | Vitamin D: 18.61 ± 8.33 ng/mL (ASD) vs. 24.62 ± 13.18 ng/mL (non-ASD), $p = 0.014$; Converted: 46.5 vs. 61.6 nmol/L; Deficiency (<20 ng/mL): ASD = 64.8%, Control = 33.3%; Multivariate adjusted OR for vitamin D deficiency in ASD = 10.31 (95% CI: 1.96–54.22), $p = 0.006$. | Vitamin D (25(OH)D). | Children with ASD had significantly lower vitamin D levels than peers with other neuropsychiatric conditions. High odds of vitamin D deficiency suggest potential etiologic or pathophysiological involvement in ASD. |
| **19** | Zou et al. (2024, China) | Observational (Case-Control & Cross-Sectional) | 230 children total (120 ASD, 110 TD); preschoolers. | Early Childhood Nutrient Status — serum levels of $B_{12}$, folate, D, homocysteine; BMI, diet, sleep habits. | ASD vs. TD; subgroups stratified by BMI, CARS, SRS, sleep, and food preference. | Vitamin $B_{12}$: 736.52 (550.4–1039.4) vs. 919 (710.4–1131.4) pg/mL, $p < 0.001$; Converted: 543.54 vs. 678.22 pmol/L Folate: 11.33 ± 3.29 vs. 12.48 ± 3.10 ng/mL, $p = 0.007$; Converted: 25.74 vs. 28.33 nmol/L. Homocysteine: 5.66 | Vitamin $B_{12}$ (pg/mL → pmol/L), Folate (ng/mL → nmol/L), Homocysteine (µmol/L), Vitamin A, | ASD children show significantly lower $B_{12}$ and folate, higher homocysteine. Nutrient status, BMI, sleep, and diet are linked to ASD symptom severity. |

| No. | Authors | Study | Population | Intervention | Comparison | Outcome | Biomarker | Data Interpretation |
|---|---|---|---|---|---|---|---|---|
| | | | | | | (4.95–6.52) vs. 5.39 (4.63–6.13) µmol/L, p = 0.047. Estimated ORs: B12 deficiency (<600 pmol/L): OR = 2.73 (95% CI: 1.55–4.81), p < 0.001.<br>Folate <25 nmol/L: OR = 1.92 (95% CI: 1.08–3.40), p = 0.025<br>Homocysteine >6.0 µmol/L: OR = 2.33 (95% CI: 1.27–4.30), p = 0.006. | Vitamin D3, BMI, Sleep disturbances, Diet preference. | |
| **20** | Eshawi et al. (2024, Libya) | Observational (Case-Control) | 47 total (27 ASD+ADHD, 20 Controls). | Early Childhood Nutrient Status — serum levels of vitamin $B_{12}$, folate, and homocysteine in children. | ASD+ADHD group vs. neurotypical controls | Homocysteine: 9.2 ± 2.9 vs. 7.22 ± 1.2 µmol/L, p = 0.002.<br>Vitamin $B_{12}$: 212.9 ± 124.2 vs. 221.3 ± 112.1 pg/mL, p = 0.064; Converted: 157.92 vs. 163.16 pmol/L.<br>Folate: 2.0 ± 0.9 vs. 3.0 ± 1.1 ng/mL, p = 0.035; Converted: 4.54 vs. 6.81 nmol/L.<br>Estimated OR (Homocysteine >8.5 µmol/L): OR = 2.93 (95% CI: 1.22–7.05), p = 0.016.<br>Estimated OR (Folate <5 nmol/L): OR = 2.62 (95% CI: 1.11–6.17), p = 0.028.<br>Estimated OR (Vitamin B12 <150 pmol/L): OR = 1.13 (95% CI: 0.36–3.62), p = 0.831. | Vitamin $B_{12}$ (pg/mL → pmol/L), Folate (ng/mL → nmol/L), Homocysteine (µmol/L). | Children with ASD+ADHD had significantly higher homocysteine and lower folate levels compared to controls. Vitamin $B_{12}$ showed no significant difference; estimated OR indicates a weak and non-significant association. |
| **21** | Yektaş et al. (2019, Turkey) | Observational (Cross-Sectional & Case-Control) | 118 children (48 ADHD, 35 ASD, 35 controls); 81.4% male. | Postnatal/Early Childhood Nutrient Status — Serum levels of | ASD vs. Control; ADHD vs. Control; ASD vs. ADHD. | Vitamin $B_{12}$ (pg/mL): ASD = 268 (IQR 407), ADHD = 929 (IQR 774), Control = 1611 (IQR 357). Converted: ASD = 197.78 | Vitamin $B_{12}$, Homocysteine, Folate. | Children with ASD had the lowest Vitamin $B_{12}$ and highest Homocysteine levels; |

| No. | Authors | Study | Population | Intervention | Comparison | Outcome | Biomarker | Data Interpretation |
|---|---|---|---|---|---|---|---|---|
| | | | | Vitamin $B_{12}$, Folate, and Homocysteine. | | pmol/L, ADHD = 686.36 pmol/L. Control = 1189.52 pmol/L. Homocysteine (μmol/L): ASD = 19.12 (IQR 5.98), ADHD = 15.82 (IQR 2.38), Control = 12.97 (IQR 4.87). Folate (ng/mL): No significant difference. Estimated ORs: ASD vs. Control: - B12: OR = 8.45 (95% CI: 3.67–19.45), $p < 0.001$. - Homocysteine: OR = 4.25 (95% CI: 2.12–8.53), $p < 0.001$. ADHD vs. Control: - B12: OR = 3.67 (95% CI: 1.85–7.29), $p < 0.001$. - Homocysteine: OR = 2.01 (95% CI: 1.02–4.01), p = 0.043. | | ADHD had intermediate levels; $B_{12}$ levels were inversely correlated with oppositionality and hyperactivity/impulsivity in ADHD; Folate levels showed no group differences. |
| | | | | **INTERVENTION TYPE 4: Postnatal Nutritional Intervention** Studies that evaluate the efficacy of nutritional supplementation (e.g., vitamin D, $B_{12}$) administered postnatally, often employing pre-post or placebo-controlled designs. | | | | |
| **22** | Hendren et al. (2016, U.S.A.) | Randomized Controlled Trial (RCT) | 57 children with ASD (27 $B_{12}$, 23 placebo; mean age = 5.3y; 79% male). | Postnatal Nutritional Intervention — subcutaneous methyl B12 (75 μg/kg) vs. placebo every 3 days for 8 weeks. | Randomized arms: methyl $B_{12}$ vs. placebo. | CGI-Improvement at 8w: $B_{12}$ = 2.4 ± 0.8, placebo = 3.1 ± 0.8; Mean difference = -0.7 (95% CI: -1.2 to -0.2), p = 0.005. Responder rate (CGI-I ≤ 2): 52% B12 vs. 26% placebo; Estimated OR = 3.05 (95% CI: 0.92–10.11), p = 0.064. No significant differences on parent-rated ABC or SRS total scores. CGI-I improvement correlated with ↑ methionine (p = 0.05), ↓ SAH (p = 0.007), ↑ | Vitamin $B_{12}$ (Methylcobalamin), Homocysteine, Methionine, SAM, SAH. | Methyl $B_{12}$ led to statistically significant improvements in clinician-rated CGI-I scores. Estimated OR suggests a 3-fold increased chance of clinical response, but without statistical significance. Response was correlated with improvements in |

| No. | Authors | Study | Population | Intervention | Comparison | Outcome | Biomarker | Data Interpretation |
|---|---|---|---|---|---|---|---|---|
| | | | | | | SAM/SAH ratio (p = 0.007). No significant change in GSH, homocysteine, or oxidative markers (e.g., GSSG). | | methylation markers (methionine, SAH, SAM/SAH). No consistent changes in oxidative stress markers. No OR calculable from continuous biomarker levels. |
| **23** | Javadfar et al. (2020, Iran) | Randomized Controlled Trial (RCT) | Total N = 43 (Vitamin D group: 22, Placebo group: 21); Age: 3–13 years; 36 boys, 7 girls. | Postnatal Nutritional Intervention — High-dose Vitamin D (300 IU/kg/day, max 6000 IU) vs. Placebo for 15 weeks. | Vitamin D group vs. Placebo group; pre-post within group; subgroup analyses based on scale changes. | Vitamin D (25(OH)D): 8.19 ± 6.78 ng/mL → 39.10 ± 33.71 ng/mL (Vitamin D group), p = 0.001; Placebo: 10.84 ± 16.80 → 8.94 ± 8.03, p = 0.728; Between-group p = 0.0001; Converted: 20.48 → 97.75 nmol/L (Vitamin D); CARS Score: -2.11 ± 3.18 vs. -0.33 ± 0.80, p = 0.021; Estimated OR for CARS improvement (≥2 points): 7.20 (95% CI: 1.63–31.71), p = 0.006; ATEC Score: -4.69 ± 5.12 vs. -0.76 ± 0.88, p = 0.020; Serotonin Δ = −4.07 ng/mL, p = 0.085; IL-6 Δ = −1.8 ng/mL, p = 0.082. | Vitamin D (25(OH)D); Serotonin; Interleukin-6 (IL-6). | Significant improvement in ASD symptoms (CARS, ATEC) with Vitamin D; Estimated OR for clinical response (CARS) = 7.20 (95% CI: 1.63–31.71), p = 0.006. No significant effect on serotonin or IL-6; Large increase in serum 25(OH)D post-intervention; high baseline deficiency (~86%); Vitamin D supplementation shows promise for symptom modulation. |
| **24** | Jayanath et al. (2021, Malaysia) | Observational (Cross-sectional) | 103 children with ASD (85.4% male); Mean age: 6.2 ± 2.4 years. | Postnatal Nutritional Intervention Study — Daily Vitamin D3 (cholecalciferol) | Vitamin D deficient (<35 nmol/L, n=20) vs. non-deficient (≥35 nmol/L, n=83); subgroup analysis | Baseline 25(OH)D: Deficient = 27.5 ± 5.2 nmol/L; Non-deficient = 50.3 ± 10.9 nmol/L, p < 0.001. Vitamin D increased from 27.3 ± 5.0 to 48.9 ± 17.6 nmol/L after 3 months (n=14), p < 0.001. ASD severity (CARS-2) improved in | Vitamin D (25(OH)D) | Vitamin D deficiency prevalent (19%) among Malaysian children with ASD. Vitamin D supplementation led to statistically significant improvements in ASD |

| No. | Authors | Study | Population | Intervention | Comparison | Outcome | Biomarker | Data Interpretation |
|---|---|---|---|---|---|---|---|---|
| | | | | 1200 IU for 3 months. | of pre/post treatment. | deficient group: Raw score 38.0 ± 7.3 to 35.9 ± 7.7, p = 0.013; Percentile score: 52.4 ± 26.9 to 43.4 ± 28.9, p = 0.023; T-score: 50.9 ± 8.9 to 48.4 ± 9.5, p = 0.023. Female gender associated with vitamin D deficiency: OR = 5.05 (95% CI: 1.56–16.31), p = 0.007. | | severity (CARS-2) scores in the deficient group. No improvement observed in behavioral domains (ABC-2). Female gender associated with higher risk of deficiency (OR = 5.05, 95% CI: 1.56–16.31). |
| **25** | Kerley et al. (2017, Ireland) | Randomized Controlled Trial (RCT) | 21 children with ASD (Vit D group: 11, Placebo: 10); Mean age: 8.4 ± 2.4 years. | Postnatal Nutritional Intervention — Oral Vitamin D3 (2000 IU daily) vs. Placebo for 20 weeks. | Randomized arms: Vitamin D vs. Placebo. | 25(OH)D baseline: Vit D = 40.5 ± 17.7 nmol/L; Placebo = 48.1 ± 21.0; p = 0.38. Post: Vit D = 89.5 ± 32.6 vs. Placebo = 53.4 ± 22.2; p = 0.003. ADOS-2 (Social Affect): Δ Vit D = -0.3 ± 0.8; Placebo = +0.2 ± 0.8; p = 0.2. Estimated OR for improvement ≥1 point: 5.14 (95% CI: 0.47–56.90), p ≈ 0.157. RRB: no change. ABAS-II and SRS: no significant change. | Vitamin D (25(OH)D) | Vitamin D supplementation significantly increased serum 25(OH)D levels. No statistically significant improvements in ASD symptoms. Estimated OR suggests possible clinical trend despite underpowered design. |
| **26** | Mazahery et al. (2019, New Zealand) | Randomized Controlled Trial (RCT) | 111 children with ASD; 4 groups: A) Vit D + Omega-3 (n=29), B) Vit D (n=27), C) Omega-3 (n=27), D) Placebo (n=28); Age 2.5–8 years; 91% male. | Postnatal Nutritional Intervention — 12-month supplementation: Vitamin D3 (2000 IU/day), Omega-3 (722 mg/day EPA+DHA), both or placebo. | Four-arm RCT: A vs. B vs. C vs. D; Pre-post and inter-group comparisons. | SRS Total Score at 12 months: A = −10.6 ± 17.1, B = −7.2 ± 16.2, C = −4.5 ± 19.0, D = −0.9 ± 12.1. A vs. D: p < 0.05. Estimated OR for >10-point improvement (A vs. D): 4.88 (95% CI: 1.35–17.65), p ≈ 0.012. Other outcomes (CSHQ, GI Index, Sensory Profile) non-significant. | Vitamin D (25(OH)D). | Combined Vitamin D and Omega-3 supplementation improved social responsiveness vs. placebo. OR = 4.88 (95% CI: 1.35–17.65), p ≈ 0.012. Suggests synergistic effect. |

| No. | Authors | Study | Population | Intervention | Comparison | Outcome | Biomarker | Data Interpretation |
|---|---|---|---|---|---|---|---|---|
| **27** | Mazahery et al. (2020, New Zealand) | Randomized Controlled Trial (RCT) | 67 children (VID=15, OM=21, VIDOM=15, Placebo=16); 52 with elevated IL-1β. | Postnatal Nutritional Interventions — Vitamin D (2000 IU/day), Omega-3 (722 mg DHA/day), Combined, or Placebo. | VID vs Placebo, OM vs Placebo, VIDOM vs Placebo; subgroup analysis based on IL-1β levels (Normal <3.2 pg/mL vs Elevated ≥3.2 pg/mL). | SRS scores (12 months):<br>- SRS-awareness (OM: P=0.01, $\eta p^2$=0.11), VIDOM: P=0.01, $\eta p^2$=0.11;<br>- SRS-total (OM: P=0.06), VIDOM: P=0.11;<br>- Subgroup (Elevated IL-1β): OM → SRS-total: P=0.01, $\eta p^2$=0.14; VIDOM → SRS-awareness: P=0.01, $\eta p^2$=0.14. Estimated OR for clinical response (>10 point improvement) in OM vs Placebo: OR = 5.25 (95% CI: 0.94–29.18), p ≈ 0.045. | Vitamin D (25(OH)D), Omega-3 Index, IL-1β. | Omega-3 and combined treatment showed benefits in ASD symptoms, particularly among children with elevated IL-1β. Estimated OR suggests clinical improvement; inflammation may modulate response. |
| **28** | Moradi et al. (2018, Iran) | Randomized Controlled Trial (RCT) | 100 total (4 groups: A=25, B=25, C=25, D=25). | Postnatal Nutritional Interventions — Group A: Perceptual-motor exercises; Group B: Vitamin D3 supplementation (300 IU/kg/day up to 5000 IU/day, 3 months); Group C: Combination; Group D: Placebo | Group A (Perceptual-motor exercises only, ASD) vs Group D (Placebo, ASD); Group B (Vitamin D supplementation only, ASD) vs Group D (Placebo, ASD); Group C (Combined Vitamin D + Exercises, ASD) vs Group D (Placebo, ASD); | GARS-2 stereotypy subscale (mean ± SD): Group A: 15.84±3.44 to 13.08±2.69, p=0.01; Group B: 17.08±3.88 to 14.76±2.80, p=0.01; Group C: 17.40±3.47 to 12.36±2.82, p=0.01; Group D: 16.28±3.19 to 15.92±4.01, p=0.51. ANCOVA: F=14.44, p=0.01. Bonferroni: C significantly better than others. Estimated OR (C vs D): 8.50 (95% CI: 2.34–30.91), p ≈ 0.0006; B vs D: OR = 5.09 (95% CI: 1.45–17.92), p ≈ 0.0087; A vs D: OR = 3.69 (95% CI: 1.05–12.96), p ≈ 0.0366; C vs A: OR = 2.30 (95% CI: 0.73–7.27), p ≈ 0.152; C vs B: | Vitamin D (25(OH)D) | All interventions showed improvement in stereotypy, with the strongest effect in the combination group. Vitamin D alone and exercise alone were both effective vs placebo. Synergistic benefit evident. Vitamin D measured via intervention dosing; serum levels not reported. |

| No. | Authors | Study | Population | Intervention | Comparison | Outcome | Biomarker | Data Interpretation |
|---|---|---|---|---|---|---|---|---|
| | | | | | Group C vs Group A and Group B to assess additive effects. | OR = 1.67 (95% CI: 0.53–5.29), p ≈ 0.382. | | |
| **29** | Saad et al. (2015, Egypt) | Observational (Case-Control Study & Open-Label Interventional Trial) | ASD = 122 (3–9y), Control = 100; Intervention (Vitamin D3) = 106 (83 completed). | Postnatal Nutritional Intervention (non-RCT) + Early Childhood Nutrient Status — Baseline 25(OH)D in ASD vs. Controls; Open-label Vitamin D3 (300 IU/kg/day; max 5000 IU) × 3 months. | ASD vs. neurotypical controls; CARS severity (severe vs. mild/moderate); Pre-Post Vitamin D3 intervention. | Baseline 25(OH)D: ASD = 18.02 ± 8.75 ng/mL (45.05 ± 21.87 nmol/L), Control = 42.51 ± 9.48 ng/mL (106.28 ± 23.70 nmol/L), $p < 0.0001$; Severe ASD = 12.16 ± 5.83 ng/mL vs. Mild/Moderate = 21.10 ± 8.47 ng/mL, $p < 0.0001$; Correlation CARS: r = –0.502, $p < 0.0001$; Post-treatment CARS: 37.7 ± 2.4 → 30.7 ± 2.8 (Δ = −7.0), $p < 0.001$; Improved: 67/83 (80.72%) after 3 months Vitamin D; ABC Subscales improved: Irritability (p = 0.021), Social withdrawal (p = 0.028), Hyperactivity (p = 0.01), Stereotypy (p = 0.04); Estimated OR for Vitamin D Deficiency (<20 ng/mL): 13.1 (95% CI: 7.2–23.8), $p < 0.001$. | Vitamin D (25(OH)D). | Vitamin D significantly lower in ASD vs. controls; inversely correlated with ASD severity; High-dose Vitamin D3 supplementation improved behavioral scores; stronger improvements when serum levels >40 ng/mL |
| **30** | Sandboge et al. (2023, Finland) | Randomized Controlled Trial (RCT) | 346 children (169: 400 IU, 177: 1200 IU). | Postnatal Nutritional Interventions — High-dose (1200 IU) vs. Standard-dose (400 IU) Vitamin D3 from | 1200 IU vs. 400 IU Subgroups: 25(OH)D <30 ng/mL vs. ≥30 ng/mL maternal level. | Clinically significant internalizing problems at age 6–8 years: 10/177 (5.6%) in 1200 IU vs. 20/169 (11.8%) in 400 IU. OR = 0.40 (95% CI: 0.17–0.94), p = 0.04. Externalizing problems: OR = 0.89 (95% CI: 0.42–1.91), p = 0.77. Total problems: OR = 0.81 | Vitamin D (25(OH)D): 1 year: 34.4 vs. 47.6 ng/mL (converted: 86.0 vs. 119.0 nmol/L). 2 years: 35.5 vs. | High-dose vitamin D3 (1200 IU) supplementation significantly reduced risk for internalizing problems in childhood. No effect observed for externalizing or total |

| No. | Authors | Study | Population | Intervention | Comparison | Outcome | Biomarker | Data Interpretation |
|---|---|---|---|---|---|---|---|---|
| | | | | 2 weeks to 2 years. | | (95% CI: 0.29–2.23), p = 0.68. Maternal 25(OH)D <30 ng/mL. higher risk unless child received 1200 IU. | 48.1 ng/mL (converted: 88.8 vs. 120.2 nmol/L). Maternal 25(OH)D: 33.7 ± 9.1 vs. 33.4 ± 7.9 ng/mL. | problems. Effect stronger in those with low maternal 25(OH)D. |
| **31** | Tuovinen et al. (2021, Finland) | Randomized Controlled Trial (RCT) | 801 total (404 received 400 IU, 397 received 1200 IU). | Postnatal Nutritional Intervention — Randomized controlled trial of high-dose (1200 IU) vs. standard-dose (400 IU) vitamin D3 supplementation from age 2 weeks to 24 months. | 1200 IU vs. 400 IU vitamin D3 supplementation. | ASQ total score at 12 mo: 45.0 ± 7.1 (400 IU) vs. 46.2 ± 7.9 (1200 IU), MD = 1.17 (95% CI: −0.06 to 2.38), p = 0.06. ASQ at 24 mo: 50.9 ± 5.3 vs. 51.5 ± 5.5, MD = 0.48 (95% CI: −0.40 to 1.36), p = 0.29. ITSEA externalizing domain score ≥1.5 SDs: OR = 2.33 (95% CI: 1.19–4.56), p = 0.01. No significant differences in internalizing, dysregulation, or competencies. | Vitamin D (25(OH)D): 33.3 ± 7.9 ng/mL vs. 46.2 ± 11.0 at 12 mo; 34.8 ± 7.8 vs. 47.3 ± 10.6 at 24 mo → Converted: 83.25 vs. 115.5 nmol/L; 87.0 vs. 118.25 nmol/L. | No significant benefit of high-dose vitamin D3 on overall neurodevelopment; increased externalizing symptoms in high-dose group. 25(OH)D levels not associated with primary outcomes. |
| **32** | Wink et al. (2016, USA) | Randomized Controlled Trial (RCT) | 31 children with ASD (NAC: 16, Placebo: 15; 25 completed). | Postnatal Nutritional Interventions — NAC (N-acetylcysteine) 60 mg/kg/day orally for 12 weeks. | NAC vs. Placebo. | CGI-I Responder Rate (score ≤2): NAC = 6/13 (46.2%) vs. Placebo = 4/12 (33.3%) → OR = 1.71, 95% CI: 0.37–7.86, p ≈ 0.49. GSH: NAC = 780.3 μM vs. Placebo = 640.4 μM, p < 0.05. GSSG: NAC = 16.7 μM vs. Placebo = 12.5 μM, p = 0.09. GSH/GSSG Ratio: no significant difference. | GSH, GSSG, Homocysteine. | NAC significantly increased GSH levels but did not significantly improve clinical CGI-I outcomes; responder OR = 1.71 (95% CI: 0.37–7.86), p ≈ 0.49. |

| No. | Authors | Study | Population | Intervention | Comparison | Outcome | Biomarker | Data Interpretation |
|---|---|---|---|---|---|---|---|---|
| | | | | | | Homocysteine: NAC = 9.9 µM vs. Placebo = 5.6 µM, p > 0.16. | | |
| **INTERVENTION TYPE 5: Gene–Nutrient Interaction** Investigations exploring the modulatory effects of genetic polymor-phisms (e.g., Methylenetetrahydrofolate Reductase, and Vitamin D Receptor) on biomarker levels or ASD phenotypes, emphasizing gene–environment interplay in neurodevelopment. | | | | | | | | |
| **33** | Coşkun et al. (2016, Turkey) | Observational (Case-Control) | 480 total (237 ASD, 243 controls); Serum 25(OH)D only in 167 (85 ASD, 82 controls); Age: ASD = 51.1±33.8 months, Control = 49.1±17.2 months; Sex: ASD = 195M/42F, Control = 205M/38F. | Gene–Nutrient Interaction; Postnatal/Early Childhood Nutrient Status — VDR gene polymorphisms (TaqI, BsmI, FokI, ApaI, Cdx2); Serum 25(OH)D measured via ELISA. | ASD vs. healthy neurotypical controls; subgroup comparisons by VDR genotype. | Serum 25(OH)D: 79.4 ± 25.9 ng/mL (ASD) vs. 65.1 ± 23.9 ng/mL (Control), p < 0.001; Converted: 198.5 vs. 162.75 nmol/L; Significant genotype association with FokI polymorphism: TT ("ff") = 96.23 ± 17.68 vs. CT ("Ff") = 71.21 ± 28.35 and CC ("FF") = 83.78 ± 23.36; Genotype frequencies (rare homozygous): TaqI CC (ASD = 17.7%, Control = 9.1%), OR = 2.16 (95% CI: 1.25–3.75), p = 0.005; BsmI AA (22.4% vs. 15.2%), OR = 1.60 (95% CI: 1.01–2.55), p = 0.045; FokI TT (7.2% vs. 2.9%), OR = 2.61 (95% CI: 1.06–6.40), p = 0.031; GTTT haplotype: OR = 2.32 (95% CI: 1.11–4.88), p = 0.022. | Vitamin D (25(OH)D), VDR genotypes. | Significant associations between VDR polymorphisms (TaqI, BsmI, FokI) and ASD; GTTT haplotype conferred 2.32-fold increased risk; Elevated serum 25(OH)D in ASD and associated with FokI TT genotype; study suggests VDR gene variations influence vitamin D metabolism and ASD risk. |
| **34** | Shom et al. (2024, India) | Observational (Case-control & Cross-sectional) | Total N = 411 ASD, 397 Controls; Plasma biomarker subset: 25 ASD, 26 Controls; | Gene–Nutrient Interaction Studies / Mechanistic/Systems Biology Models. | ASD vs. Neurotypical; stratified by genotypes rs7041, rs4588, rs3755967 and GC isoforms. | Plasma 25(OH)D (ng/mL): 10.52 ± 1.32 (ASD) vs. 18.48 ± 1.13 (Control), p < 0.001; Converted: 26.30 vs. 46.20 nmol/L \n DBP (µg/mL): 24.60 ± 0.83 (ASD) vs. 50.64 ± 10.57 (Control), p = 0.015 \n DBP mRNA expression: | Vitamin D (25(OH)D), DBP (Vitamin D Binding Protein), DBP mRNA. | Lower 25(OH)D and DBP in ASD probands. Genetic variants associated with severity and lower DBP expression. Suggests vitamin D malfunction due to deficient DBP. |

| No. | Authors | Study | Population | Intervention | Comparison | Outcome | Biomarker | Data Interpretation |
|---|---|---|---|---|---|---|---|---|
| | | | DBP mRNA: 74 ASD, 44 Controls. | | | 34.48-fold downregulated in ASD, p < 0.0001 \n Genetic association: rs7041 'CC' (p = 0.04), rs4588 'TT' (p = 0.004), rs3755967 'TT' (p = 0.0005) with CARS2-ST scores \n Haplotype analysis: 'C-T-C' (rs7041–rs4588–rs3755967) less frequent in ASD, OR = 0.34 (95% CI: 0.14–0.78), p = 0.04. | | Highlights gene–environment interaction in ASD etiology. |
| | | | | | | | | |
| | | | | **INTERVENTION TYPE 6: Mechanistic/Systems Biology Models** In silico models, computational simulations, or pathway-based analyses that investigate nutrient-influenced molecular networks re-levant to ASD, typically without involving human subjects. | | | | |
| **35** | De Marzio et al. (2024, U.S.A.) | Observational (Prospective, systems biology) | n = 381 children from VDAART (blood samples and communication screening at age 3); maternal data collected at 32–38 weeks gestation and 1-year postpartum. | Postnatal/Early Childhood Nutrient Status + Prenatal Nutrient Exposure + Mechanistic/Systems Biology Model. | Comparison of ASQ-comm score subgroups (On Schedule, Monitoring, Evaluation Needed); stratified by maternal and child vitamin D levels. | Maternal Vitamin D (32–38 weeks): low levels associated with lower ASQ-comm score; Interaction vitamin D × ASQ score: significant metabolomic pathway activation (tryptophan/serotonin). Key edge 5HIAA↔L-kynurenine: p = 0.0064; Serotonin/5HIAA ratio positively associated with ASQ-comm (p = 0.038); No OR reported. | Vitamin D (nmol/L); Metabolites: 5HIAA, L-kynurenine, serotonin. | Significant metabolic effects of low maternal and child vitD on tryptophan-serotonin pathways affecting communication; supports mechanistic pathway model for vitamin D–ASD risk. |

**Notes:**

- **5HIAA**: 5-hydroxyindoleacetic acid.
- **25(OH)D**: 25-Hydroxyvitamin D (formă circulantă a vitaminei D).
- **ABAS-II:** Adaptive Behavior Assessment System, Second Edition.
- **ABC-C**: Aberrant Behavior Checklist – Community Version.

| No. | Authors | Study | Population | Intervention | Comparison | Outcome | Biomarker | Data Interpretation |
|---|---|---|---|---|---|---|---|---|

- **ABCD**: Amsterdam Born Children and their Development cohort.
- **ADHD**: Attention-Deficit/Hyperactivity Disorder.
- **ADHD-RS**: Attention-Deficit/Hyperactivity Disorder Rating Scale.
- **ADOS-2:** Autism Diagnostic Observation Schedule, Second Edition.
- **ASD**: Autism Spectrum Disorder.
- **ASQ**-comm: Ages and Stages Questionnaire - Communication Domain.
- **ATEC**: Autism Treatment Evaluation Checklist.
- **Bayley-III:** Bayley Scales of Infant and Toddler Development, Third Edition
- **BMI**: Body Mass Index.
- **CARS**: Childhood Autism Rating Scale.
- **CARS2-ST**: Childhood Autism Rating Scale, Second Edition – Standard Test.
- **CC ("FF")**: Homozygous for the major allele of the FokI polymorphism → Typically associated with higher VDR activity.
- **CI**: Confidence Interval.
- **CGI-I:** Clinical Global Impression – Improvement Scale.
- **CRP**: C-Reactive Protein.
- **CT ("Ff")**: Heterozygous for the FokI polymorphism→ One major (F) and one minor (f) allele; intermediate activity.
- **CSHQ**: Children's Sleep Habits Questionnaire.
- **DBP**: Vitamin D Binding Protein.
- **DHA**: Docosahexaenoic Acid.
- **DOB**: Date of Birth.
- **ECLIA**: Electrochemiluminescence Immunoassay.
- **ELISA**: Enzyme-Linked Immunosorbent Assay.
- **EPA**: Eicosapentaenoic Acid.
- **GC isoforms**: Genetic isoforms of group-specific component (vitamin D binding protein gene).
- **Genetic Polymorphisms:** TaqI (rs731236): Located in exon 9 of the VDR gene; may affect mRNA stability. BsmI (rs1544410): In intron 8; may influence gene expression via mRNA regulation. FokI (rs2228570): In exon 2; alters VDR protein length and function (functional SNP). ApaI (rs7975232): In intron 8; may affect transcriptional regulation.
- **GI Index**: Gastrointestinal Severity Index.
- **GW**: Gestational Week.
- **GTTT (VDR haplotype)**: Refers to a specific allele combination across four Vitamin D Receptor (VDR) polymorphisms: ApaI major allele (G); BsmI minor allele (T); TaqI minor allele (T); FokI minor allele (T).
- **GSH**: Glutathione.
- **GSSG**: Glutathione Disulfide.
- **IL-6**: Interleukin-6 (a pro-inflammatory cytokine).
- **ITSEA**: Infant-Toddler Social Emotional Assessment — A validated tool for assessing social-emotional problems and competencies in toddlers.
- **ITT**: Intention-To-Treat (Analysis).

| No. | Authors | Study | Population | Intervention | Comparison | Outcome | Biomarker | Data Interpretation |
|---|---|---|---|---|---|---|---|---|

- **IU**: International Units. Cdx2 (rs11568820): In the VDR promoter; affects binding of transcription factor Cdx2, influencing gene expression.
- **KTR:** Kynurenine-to-Tryptophan Ratio
- **LC-MS/MS**: Liquid Chromatography–Tandem Mass Spectrometry.
- **LCPUFA**: Long-Chain Polyunsaturated Fatty Acids.
- **LIONESS**: Linear Interpolation to Obtain Network Estimates for Single Samples.
- **MARBLES**: Markers of Autism Risk in Babies – Learning Early Signs.
- **mmol/L**: Millimoles per Liter.
- **mRNA**: Messenger Ribonucleic Acid.
- **NAC**: N-acetylcysteine.
- **ng/ml**: Nanograms per Milliliter.
- **nmol/L**: Nanomoles per Liter.
- **OR**: Odds Ratio.
- **p**: p-value (statistical significance level).
- **PDD-NOS**: Pervasive Developmental Disorder - Not Otherwise Specified.
- **pg/ml**: Picograms per Milliliter.
- **qPCR**: Quantitative Polymerase Chain Reaction.
- **RIA**: Radioimmunoassay.
- **RCT**: Randomized Controlled Trial.
- **RRB:** Restricted and Repetitive Behaviors (ADOS-2 domain).
- **rs7041/rs4588/rs3755967**: Single Nucleotide Polymorphisms (SNPs) in the GC gene.
- **SAH**: S-adenosylhomocysteine.
- **SAM**: S-adenosylmethionine.
- **SD**: Standard Deviation.
- **SDQ**: Strengths and Difficulties Questionnaire.
- **SES**: Socioeconomic Status.
- **SMD**: Standardized Mean Difference.
- **TD:** Typically Developing children.
- **TT ("ff")**: Homozygous for the minor allele of the FokI polymorphism→ Indicates reduced activity of the Vitamin D Receptor (VDR).
- **µg/dL**: Micrograms per Deciliter.
- **µmol/L**: Micromoles per Liter.
- **VDAART**: Vitamin D Antenatal Asthma Reduction Trial.
- **VDR**: Vitamin D Receptor.

**Citation**: All references are cited in the main text.

**Table S6**. Risk of Bias Assessment Using the RoB 2 Tool for Randomized Controlled Trials and Certainty of Evidence Ratings According to the GRADE Framework.

| No. | Study (Author, Year/Country) | Study Design | D1 (Authors' Judgement/ Support) | D2 (Authors' Judgement/ Support) | D3 (Authors' Judgement/ Support) | D4 (Authors' Judgement/ Support) | D5 (Authors' Judgement/ Support) | Overall RoB 2 (Authors' Judgement/ Support) | GRADE Certainty (Authors' Judgement/ Support) |
|---|---|---|---|---|---|---|---|---|---|
| **1** | Aagaard et al., 2024, Denmark | Randomized Controlled Trial (RCT) | Low (Central randomization and allocation concealment procedures well-documented). | Low (No deviations from intended intervention; adherence verified). | Low (Low attrition and missing data handled appropriately). | Low (Outcome assessment blinded; validated tools used: K-SADS-PL, SRS-2, ADHD-RS). | Low (Protocol pre-registered; full results reported with subgroup analyses). | Low (Robust design, minimal bias across domains). | Moderate (Downgraded for imprecision (low event counts for ASD); otherwise high quality). |
| **2** | Hendren et al., 2016, U.S.A. | Randomized Controlled Trial (RCT) | Some concerns (Randomization mentioned, but sequence generation and allocation concealment not clearly described). | Low (Double-blinded design with indistinguishabl e placebo and active injections) | Low (Minimal attrition; Intention-to-Treat analysis used). | Some concerns (Clinician-rated outcomes blinded; unclear for secondary parent-rated tools) | High (No pre-registration; multiple secondary outcomes selectively reported). | High (Due to lack of protocol registration and potential selective reporting). | Low (High RoB, small sample size, exploratory biomarker analysis limits generalizability). |
| **3** | Javadfar et al., 2020, Iran | Randomized Controlled Trial (RCT) | Some concerns (Randomization mentioned, but sequence generation and allocation concealment not clearly described). | Low (Blinding of participants and assessors described; use of placebo). | Low (Minimal attrition; data presented for all participants) | Low [Standardized scales (CARS, ATEC) used by trained clinicians]. | Some concerns (No registered protocol; multiple exploratory outcomes analyzed). | Some concerns (Primarily due to unclear randomization process and lack of preregistration). | Moderate (Positive findings on primary outcomes, limited by small sample and exploratory biomarkers). |

| No. | Study (Author, Year/Country) | Study Design | D1 (Authors' Judgement/ Support) | D2 (Authors' Judgement/ Support) | D3 (Authors' Judgement/ Support) | D4 (Authors' Judgement/ Support) | D5 (Authors' Judgement/ Support) | Overall RoB 2 (Authors' Judgement/ Support) | GRADE Certainty (Authors' Judgement/ Support) |
|---|---|---|---|---|---|---|---|---|---|
| **4** | Kerley et al., 2017, Ireland | Randomized Controlled Trial (RCT) | Some concerns (Randomization mentioned but method and concealment process not detailed). | Low (Placebo-controlled, double-blinded intervention) | Low (Complete outcome data available for all participants). | Low (Use of standardized tools (ADOS-2, ABAS-II, SRS); outcome assessors likely blinded). | Some concerns (No pre-registered protocol; multiple secondary outcomes, potential for selective reporting) | Some concerns (Due to lack of protocol registration and unclear randomization details). | Moderate (High internal validity, but small sample and exploratory nature reduce confidence). |
| **5** | Mazahery et al., 2019, New Zealand | Randomized Controlled Trial (RCT) | Low (Randomization method described and allocation concealment ensured). | Low (Double-blinded design, identical placebo capsules). | Low (Low attrition and reasons for withdrawal reported). | Low (Validated outcome measures (SRS, CSHQ, GI Index); assessors blinded). | Low (Outcomes prespecified, trial registered, full reporting). | Low (High methodological quality, all domains satisfied). | High (Large sample, low bias, precise outcome estimates, valid instruments). |
| **6** | Mazahery et al., 2020, New Zealand | Randomized Controlled Trial (RCT) | Low (Randomization and allocation concealment described). | Low (Double-blind, identical capsules) | Low (Low attrition, clear dropout reporting). | Low (Validated outcomes, blinded assessors). | Low (Prespecified protocol, full reporting). | Low (All domains satisfied with low bias). | High (Robust design, moderate sample, validated outcomes). |
| **7** | Moradi et al., 2018, Iran | Randomized Controlled Trial (RCT) | Some Concerns (Randomization mentioned but not fully described; no allocation concealment info). | Low (Four-arm design with matching procedures across groups). | Low (No attrition reported, all participants analyzed). | Some Concerns (No mention of blinding or independent outcome assessment). | Low (Outcomes reported for all arms, consistent with objectives). | Some Concerns (Randomization and blinding insufficiently detailed). | Moderate (Strong effect sizes, but some methodological limitations in bias protection). |

| No. | Study (Author, Year/Country) | Study Design | D1 (Authors' Judgement/ Support) | D2 (Authors' Judgement/ Support) | D3 (Authors' Judgement/ Support) | D4 (Authors' Judgement/ Support) | D5 (Authors' Judgement/ Support) | Overall RoB 2 (Authors' Judgement/ Support) | GRADE Certainty (Authors' Judgement/ Support) |
|---|---|---|---|---|---|---|---|---|---|
| **8** | Saas et al. (2020, Denmark) | Randomized Controlled Trial (RCT) | Low (Central randomization, allocation concealed). | Low (Protocolized dosing, adherence monitored). | Low (Minimal attrition, ITT analysis). | Low (Validated neurodevelopmental tools (Bayley-III, SDQ) | Low (Prespecified primary outcomes, reported in full). | Low (All domains low risk; robust methodology). | Moderate (Well-conducted RCT; imprecision in effect estimates; limited generalizability due to co-supplementation). |
| **9** | Sandboge et al., 2023, Finland | Randomized Controlled Trial (RCT) | Low (Randomized with adequate concealment, no baseline imbalances). | Low (Adherence monitored, no protocol deviations). | Low (Minimal attrition, ITT analysis). | Low (Standardized assessments for child behavior outcomes). | Low (Outcomes prespecified and fully reported). | Low (No major bias risks identified in any domain). | Moderate (High internal validity, but limited behavioral outcome domains and generalizability). |
| **10** | Tuovinen et al., 2021, Finland | Randomized Controlled Trial (RCT) | Low (Central computerized randomization with allocation concealment). | Low (High adherence reported; similar dropout across arms). | Low (Low attrition; ITT analysis used). | Low (Validated tools (ASQ, ITSEA) for neurodevelopmental outcomes). | Low (All primary and secondary outcomes reported). | Low (All domains judged as low risk). | Moderate (High internal validity; generalizability limited to healthy term infants in high-latitude populations). |

| No. | Study (Author, Year/Country) | Study Design | D1 (Authors' Judgement/ Support) | D2 (Authors' Judgement/ Support) | D3 (Authors' Judgement/ Support) | D4 (Authors' Judgement/ Support) | D5 (Authors' Judgement/ Support) | Overall RoB 2 (Authors' Judgement/ Support) | GRADE Certainty (Authors' Judgement/ Support) |
|---|---|---|---|---|---|---|---|---|---|
| **11** | Wink et al., 2016, U.S.A. | Randomized Controlled Trial (RCT) | Low (Randomized, though allocation details not fully reported). | Some concerns (Potential unblinding due to parental awareness or expectations). | Low (25/31 participants completed the study-low attrition) | Low (CGI-I instrument used; blinded raters likely). | Low (Reported as per trial protocol). | Some concerns (Risk due to reporting transparency and small sample). | Low (Imprecise estimates, small sample, indirectness regarding ASD symptom improvement). |

**Notes:**

- **ABAS-II**: Adaptive Behavior Assessment System, Second Edition.
- **ADHD**: Attention-Deficit/Hyperactivity Disorder.
- **ADHD-RS**: Attention-Deficit/Hyperactivity Disorder Rating Scale.
- **ADOS-2:** Autism Diagnostic Observation Schedule, Second Edition.
- **ASQ-comm**: Ages and Stages Questionnaire - Communication Domain.
- **ATEC**: Autism Treatment Evaluation Checklist.
- **Bayley-III**: Bayley Scales of Infant and Toddler Development, Third Edition
- **CARS:** Childhood Autism Rating Scale.
- **CSHQ**: Children's Sleep Habits Questionnaire.
- **GI Index**: Gastrointestinal Severity Index.
- **ITSEA:** Infant-Toddler Social Emotional Assessment — A validated tool for assessing social-emotional problems and competencies in toddlers.
- **ITT**: Intention-To-Treat (Analysis).
- **K-SADS-PL**: Kiddie Schedule for Affective Disorders and Schizophrenia – Present and Lifetime Version.
- **RCT**: Randomized Controlled Trial.
- **SDQ**: Strengths and Difficulties Questionnaire.
- **SRS-2**: Social Responsiveness Scale, Second Edition.
- **Cochrane Risk of Bias 2 (RoB 2)**: The Cochrane RoB 2 tool is the current gold-standard framework developed to evaluate the risk of bias in randomized controlled trials (RCTs). Unlike older tools, RoB 2 does not rely on numerical scoring; instead, it provides qualitative judgments for each domain, culminating in an overall risk of bias rating for the study. Each domain is assessed using three possible ratings:
    - Low Risk of Bias.
    - Some Concerns.

| No. | Study (Author, Year/Country) | Study Design | D1 (Authors' Judgement/ Support) | D2 (Authors' Judgement/ Support) | D3 (Authors' Judgement/ Support) | D4 (Authors' Judgement/ Support) | D5 (Authors' Judgement/ Support) | Overall RoB 2 (Authors' Judgement/ Support) | GRADE Certainty (Authors' Judgement/ Support) |
|---|---|---|---|---|---|---|---|---|---|

    - High Risk of Bias.
- **RoB 2 evaluates five distinct domains of potential bias**:
    - D1: Bias arising from the randomization process.
    - D2: Bias due to deviations from intended interventions.
    - D3: Bias due to missing outcome data.
    - D4: Bias in measurement of the outcome.
    - D5: Bias in selection of the reported result.
- **Overall Risk of Bias Judgment (RoB 2 Overall)**: In accordance with the Cochrane Handbook for Systematic Reviews of Interventions, the overall risk of bias judgment for an RCT is derived based on the following criteria:
    - Low Risk of Bias: All five domains are rated as "Low Risk".
    - Some Concerns: At least one domain is rated as "Some Concerns" but none are rated as "High Risk".
    - High Risk of Bias: One or more domains are rated as "High Risk", or multiple domains raise "Some Concerns" that collectively reduce confidence in the results.

**Citation**: All references are cited in the main text.

**Table S7.** Methodological Quality Assessment of Observational Studies Using the Newcastle-Ottawa Scale (NOS) and Certainty of Evidence Evaluation According to GRADE Criteria.

| No. | Study (Author, Year, Country) | Study Design | GRADE Certainty (Authors' Judgement/ Support) | Selection NOS stars (Authors' Judgement/ Support) | Comparability NOS stars (Authors' Judgement/ Support) | Exposure/ Outcome NOS stars (Authors' Judgement/ Support) | Total NOS Score (Authors' Judgement/ Support) |
|---|---|---|---|---|---|---|---|
| **1** | Altun et al., 2018, Turkey | Observational (Case-Control) | Low (Downgraded for potential confounding, indirectness in biomarker-function link; observational design). | 4 (Clear case definition, representativeness, selection of controls appropriate). | 1 (Matched on age, sex, season; not adjusted for socioeconomic status or diet). | 2 (Standardized serum assays used; same method for both groups). | 7 (Good quality case-control study with biomarker focus). |
| **2** | Arastoo et al., 2018, Iran | Observational (Case-Control) | Low (Observational design, indirect evidence for causality, limited outcome scope, small sample size). | 4 (Well-defined cases, random sampling of controls, adequate matching). | 1 (Controlled for age, sex, socioeconomic status, sun exposure). | 2 (Standardized lab measurement ELISA-Enzyme-Linked Immunosorbent Assay, blinding not reported). | 7 (Methodologically sound with limitations in external validity and blinding). |
| **3** | Bener et al., 2017, Qatar | Observational (Case-Control) | Moderate (Large sample size, multivariate adjustment, standardized lab assays; downgraded for observational design). | 4 (Clearly defined case/control selection, representative, comparable setting). | 2 (Adjusted for key confounders: BMI (Body Mass Index), sun exposure, consanguinity). | 3 (Blinded lab testing, consistent procedures, same assays for both groups). | 9 (High-quality observational study with strong internal validity). |

| No. | Study (Author, Year, Country) | Study Design | GRADE Certainty (Authors' Judgement/ Support) | Selection NOS stars (Authors' Judgement/ Support) | Comparability NOS stars (Authors' Judgement/ Support) | Exposure/ Outcome NOS stars (Authors' Judgement/ Support) | Total NOS Score (Authors' Judgement/ Support) |
|---|---|---|---|---|---|---|---|
| **4** | Bičíková et al., 2019, Czech Republic | Observational (Case-Control) | Low (Observational design, small sample size, lack of OR reporting and exact deficiency frequencies). | 3 (Clearly defined cases/controls; limited geographic scope). | 1 (Matched for age and season only). | 2 (ECLIA measurement, blinded assessment not reported). | 6 (Acceptable quality with some design limitations). |
| **5** | Coşkun et al., 2016, Turkey | Observational (Case-Control) | Moderate (Large sample, well-matched controls, genotype analysis; no longitudinal follow-up). | 4 (Clear case definition, representative and validated ASD diagnosis). | 2 (Matched on age, sex; genotype stratification). | 3 (Genetic and biomarker data; ELISA, blinded lab assays). | 9 (High methodological quality). |
| **6** | De Marzio et al., 2024, U.S.A. | Observational (Prospective, systems biology) | Moderate (Advanced modeling, high metabolomic rigor; lacks control group comparison). | 3 (Defined cohort from RCT, clear exposure definition). | 1 (No direct ASD vs. control, but stratified by ASQ groups). | 3 (Validated biomarkers, structured metabolomic analysis). | 7 (Moderate-to-good methodological quality). |
| **7** | Egorova et al., 2020, Sweden | Observational (Retrospective case-control) | Moderate (Prospective design, good biomarker precision, potential residual confounding). | 4 (Representativeness of exposed cohort; clear ASD diagnosis and follow-up). | 2 (Adjusted for maternal age, education, BMI, parity, season). | 3 (Laboratory-measured biomarkers; structured ASD registry data). | 9 (High quality). |
| **8** | Eshawi et al. (2024, Libya) | Observational (Case-Control) | Low (Small sample, no multivariate adjustment). | 3 (Clear definition but limited generalizability). | 1 (Basic demographic matching only-age, sex). | 2 (Serum biomarkers measured with validated tests). | 6 (Moderate quality, limited confounder control). |

| No. | Study (Author, Year, Country) | Study Design | GRADE Certainty (Authors' Judgement/ Support) | Selection NOS stars (Authors' Judgement/ Support) | Comparability NOS stars (Authors' Judgement/ Support) | Exposure/ Outcome NOS stars (Authors' Judgement/ Support) | Total NOS Score (Authors' Judgement/ Support) |
|---|---|---|---|---|---|---|---|
| **9** | Jayanath et al., 2021, Malaysia | Observational (Cross-sectional) | Low (Single-center, pre-post design without control group; small sample for supplementation subgroup). | 3 (Clear definition of ASD, representativeness, and ascertainment of exposure). | 1 (Adjusted for gender only). | 2 (Pre/post outcomes clearly measured with validated tool-CARS-2; no blind assessment). | 6 (Acceptable quality; lacked control group and adjustment for additional confounders). |
| **10** | Li et al., 2024, China | Observational (Case-Control) | Moderate (Large sample size, clear biomarker definitions, multiple comparisons well-analyzed). | 4 (Representative sample, validated ASD diagnosis, structured assessment, exposure measured before outcome). | 2 (Adjusted for key confounders: age, sex, BMI, etc.) | 2 (Reliable lab assays, clear statistical outcome analysis) | 8 (High quality observational study with rigorous design and analysis). |
| **11** | Li et al., 2022, China | Observational (Case-Control) | Moderate (Biomarker quartile design, sample matched for confounders, robust OR estimates). | 4 (Well-defined cases and controls, validated ASD diagnosis, good exposure ascertainment). | 2 (Adjusted for age, sex, parental education). | 2 (Lab-based biomarker measurement, statistical comparison with CI). | 8 (Strong methodological design, representative population, matched controls). |
| **12** | Nesa et al., 2022, Bangladesh | Observational (Case-Control) | Moderate (Large effect sizes and biological plausibility, but study is single-centered with moderate sample size). | 4 (Cases defined with standard diagnostic tools (DSM-5, ADOS), controls selected from the community, no selection bias). | 1 (Controlled for age and sex, but not for SES, diet or environmental exposures). | 2 (Blood sample biomarkers collected using standard methods, same method for cases and controls). | 7 (Overall good quality, minimal bias, but limited adjustment for confounding). |

| No. | Study (Author, Year, Country) | Study Design | GRADE Certainty (Authors' Judgement/ Support) | Selection NOS stars (Authors' Judgement/ Support) | Comparability NOS stars (Authors' Judgement/ Support) | Exposure/ Outcome NOS stars (Authors' Judgement/ Support) | Total NOS Score (Authors' Judgement/ Support) |
|---|---|---|---|---|---|---|---|
| **13** | Petruzzelli et al., 2020, Italy | Observational (Case-Control) | Moderate (Statistically significant and clinically relevant effect; moderate sample; limited confounder adjustment) | 4 (ASD diagnosis using DSM-5; controls from neuropsychiatric clinic; consistent criteria and sampling). | 1 (Matched for age and sex, but not adjusted for other factors such as SES, diet). | 2 [Serum 25(OH)D measured using standard laboratory assay; consistent between groups]. | 7 (Strong design but limited covariate control lowers comparability rating). |
| **14** | Raghavan et al., 2017, USA. | Observational (Prospective Cohort) | Moderate (Large prospective cohort with biomarker data and adjusted models). | 4 (Representativeness of cohort, selection of non-exposed, exposure ascertainment, outcome not present at start). | 2 (Adjusted for multiple confounders including SES, maternal health). | 3 (Record linkage, outcome assessment blind, adequate follow-up). | 9 (Strong methodology with minimal risk of bias). |
| **15** | Saad et al., 2015, Egypt | Observational (Case-Control Study & Open-Label Interventional Trial) | Moderate (Strong results, biologically plausible; no randomization or blinding). | 3 (ASD diagnosis confirmed; controls clearly defined but not population-based; sample may not be representative). | 1 (Basic subgroup comparisons; no adjustment for confounders). | 2 (CARS and ABC used; same assessors; no blinding; clear outcome definitions). | 6 (Moderate quality due to lack of randomization and full control adjustment). |
| **16** | Schmidt et al., 2019, U.S.A. | Observational (Prospective Cohort) | Moderate (Longitudinal design, validated diagnostic tools, but limited generalizability and modest sample size). | 3 (Clearly defined ASD and control groups from MARBLES cohort; good follow-up). | 2 (Adjusted for key covariates, e.g., maternal age, ethnicity, SES). | 3 (Validated maternal blood biomarker measures and ASD diagnostic assessments). | 8 (High-quality cohort with robust biomarker data and appropriate analysis). |

| No. | Study (Author, Year, Country) | Study Design | GRADE Certainty (Authors' Judgement/ Support) | Selection NOS stars (Authors' Judgement/ Support) | Comparability NOS stars (Authors' Judgement/ Support) | Exposure/ Outcome NOS stars (Authors' Judgement/ Support) | Total NOS Score (Authors' Judgement/ Support) |
|---|---|---|---|---|---|---|---|
| **17** | Shom et al., 2024, India | Observational (Case-control & Cross-sectional) | Moderate (Strong gene–biomarker associations, but limited by cross-sectional design, modest sample for biomarker subset). | 3 (ASD diagnosis confirmed; neurotypical controls clearly defined and recruited from same setting). | 1 (Limited adjustment for potential confounders, e.g., sunlight, diet) | 3 (Validated ELISA and qPCR for biomarker/genetic expression; consistent methodology). | 7 (Solid exposure measurement and outcomes; partial limitations in confounding control). |
| **18** | Sourander et al., 2023, Finland | Observational (Nested Case-Control) | Moderate (High-quality registry data, reliable biomarker measures, but possible residual confounding and effect heterogeneity). | 3 (Nationwide registry cohort, matched controls, complete follow-up). | 2 (Matched on DOB, sex, location; adjusted for maternal age, smoking, SES) | 3 (Serum vitamin B12 measured from first trimester; ASD diagnosis from national registry). | 8 (Robust data sources and control for confounding; high methodological quality). |
| **19** | Vinkhuyzen et al., 2017, Netherlands | Observational (Nested Case-Control) | Moderate (Robust biomarker measurement (LC-MS/MS), large cohort, but relatively few ASD cases and observational design). | 3 (Population-based prospective birth cohort with standardized ASD diagnostic verification). | 2 (Adjusted for multiple confounders including season, maternal age, ethnicity, education). | 3 (High-quality LC-MS/MS exposure data and registry-verified ASD outcomes). | 8 (Excellent methodology, large sample, adjusted for key confounders). |
| **20** | Vinkhuyzen et al., 2018, Netherlands | Observational (Cohort) | Moderate (Strong methodology with standardized biomarker measurement; small ASD sample; residual confounding possible). | 3 (Population birth cohort- ABCD; ASD verified via national registry). | 2 (Controlled for maternal age, education, ethnicity, folate/B12 interaction). | 3 (Maternal biomarkers from serum; outcome registry-verified). | 8 (Methodologically strong with high-quality biomarker and ASD outcome data). |

| No. | Study (Author, Year, Country) | Study Design | GRADE Certainty (Authors' Judgement/ Support) | Selection NOS stars (Authors' Judgement/ Support) | Comparability NOS stars (Authors' Judgement/ Support) | Exposure/ Outcome NOS stars (Authors' Judgement/ Support) | Total NOS Score (Authors' Judgement/ Support) |
|---|---|---|---|---|---|---|---|
| **21** | Windham et al., 2020, U.S.A. | Observational (Case-Control) | Moderate (Large sample, registry-verified ASD diagnoses, standardized biomarker measurement; some residual confounding). | 3 (Population-based recruitment, registry-confirmed ASD/ID diagnosis). | 2 (Adjusted for maternal race, BMI, season, smoking, education, parity). | 3 (LC-MS/MS vitamin D measures; outcomes via validated state registry). | 8 (High-quality data and analysis; observational design limits causal inference). |
| **22** | Wu et al. (2018, China) | Observational (Nested Case-Control) | Moderate (Large sample, DBS biomarkers, adjusted models). | 4 (Population-based cohort, neonatal biomarker measurement). | 2 (Adjusted for confounders (sex, season, gestational age). | 3 (Registry-confirmed ASD diagnosis, validated biomarker). | 9 (Maximum quality score; high confidence observational evidence). |
| **23** | Yektaş et al. (2019, Turkey) | Observational (Cross-Sectional & Case-Control) | Moderate (Adequate sample size; subgroup comparison; use of IQRs; ORs estimated). | 4 (Clearly defined ASD, ADHD, and control groups; structured recruitment). | 2 (Adjusted for sex; appropriate stratified comparisons). | 3 (Serum biomarkers measured with validated assays and proper statistical reporting). | 9 (High quality across all domains). |
| **24** | Zou et al. (2024, China) | Observational (Case-Control & Cross-Sectional) | Moderate (Clear comparisons; multiple biomarkers; adjusted for sex, BMI, diet, and sleep). | 4 (ASD and TD groups defined; recruited from structured settings). | 2 (Stratified analyses adjusting for confounders). | 3 (Valid biomarker assays and validated outcome tools -CARS, SRS). | 9 (Maximum score, robust quality across all domains). |

**Notes:**

- **ABCD**: Amsterdam Born Children and their Development cohort.
- **ASD**: Autism Spectrum Disorder.
- **ASQ-comm**: Ages and Stages Questionnaire - Communication Domain.
- **BMI**: Body Mass Index.
- **CARS**: Childhood Autism Rating Scale.
- **DBS**: Dried Blood Spots.
- **DOB**: Date of Birth.
- **ECLIA**: Electrochemiluminescence Immunoassay.
- **qPCR**: Quantitative Polymerase Chain Reaction.
- **LC-MS/MS**: Liquid Chromatography–Tandem Mass Spectrometry.
- **RCT**: Randomized Controlled Trial.
- **SES**: Socioeconomic Status.
- **Newcastle-Ottawa Scale (NOS)**: The Newcastle-Ottawa Scale is a validated tool designed to assess the methodological quality of non-randomized observational studies, including cohort and case-control designs. It employs a star-based rating system to evaluate studies across three domains: Selection, Comparability, and Exposure (or Outcome). The maximum achievable score is 9 stars, indicating the highest methodological rigor.
- **Total NOS Score**: The Total NOS Score represents the sum of stars awarded to a study based on the NOS criteria. It reflects the study-level risk of bias and is used to gauge the overall methodological quality of observational studies, including cohort, case-control, and occasionally cross-sectional designs.
- **NOS Scoring Domains**:
  - Selection (Maximum: 4 stars): Assesses the adequacy of participant selection and case definition.
  - Comparability (Maximum: 2 stars): Evaluates the study's control of confounding variables.
  - Exposure or Outcome (Maximum: 3 stars): Assesses the ascertainment of exposure (in case-control studies) or outcome (in cohort studies), and follow-up adequacy.
  - Maximum Possible Score: 9 stars.
- **GRADE (Grading of Recommendations, Assessment, Development, and Evaluations)**: The GRADE approach is a structured framework used to assess the certainty (quality) of evidence across multiple studies for specific outcomes in systematic reviews or guidelines. Unlike NOS, GRADE evaluates outcome-level certainty rather than individual study quality and is applicable to any study design (randomized or observational).
- **GRADE assesses five domains that may lead to downgrading the certainty of evidence**:
  - Risk of bias (informed by NOS or tools like RoB 2).
  - Inconsistency (heterogeneity in results across studies).
  - Indirectness (applicability to the research question).
  - Imprecision (wide confidence intervals or small sample sizes).
  - Publication bias (selective reporting).
- **GRADE also allows upgrading observational evidence when certain conditions are met, such as**:
  - Presence of a large effect size.
  - Dose-response gradient.
  - Plausibility that residual confounding would reduce the observed effect.
- **Citation**: All references are cited in the main text.

**Table S8.** Effect Estimates and Study Characteristics of Randomized Controlled Trials (RCTs) on Vitamin D Biomarkers and Autism Spectrum Disorder (ASD): Forest Plot Data Summary.

| No. | Authors (year, country) | Study Design | log OR | OR | 95% CI | SE | Weight (%) | t | p-value |
|---|---|---|---|---|---|---|---|---|---|
| 1 | Aagaard et al. (2024, Denmark) | RCT | -0.2790 | 0.76 | 0.59–0.97 | 0.1268 | 14.9 | | |
| 2 | Saas et al. (2020, Denmark) | RCT | -0.2744 | 0.76 | 0.53–1.09 | 0.1839 | 14.7 | | |
| 3 | Javadfar et al. (2020, Iran) | RCT | 1.9726 | 7.20 | 1.63–31.71 | 0.7572 | 9.4 | | |
| 4 | Kerley et al. (2017, Ireland) | RCT | 1.6431 | 5.14 | 0.47–56.90 | 1.2236 | 5.8 | | |
| 5 | Mazahery et al. (2019, New Zealand) | RCT | 1.5854 | 4.88 | 1.35–17.65 | 0.6558 | 10.4 | | |
| 6 | Mazahery et al. (2020, New Zealand) | RCT | 1.6558 | 5.25 | 0.94–29.18 | 0.8764 | 8.3 | | |
| 7 | Moradi et al. (2018, Iran) | RCT | 1.6287 | 5.09 | 1.45–17.92 | 0.6414 | 10.5 | | |
| 8 | Sandboge et al. (2023, Finland) | RCT | -0.9169 | 0.40 | 0.17–0.94 | 0.4363 | 12.6 | | |
| 9 | Tuovinen et al. (2021, Finland) | RCT | 0.8456 | 2.33 | 1.19–4.56 | 0.3427 | 13.5 | | |
| | Random Effects Model | REM | | 1.95 | 0.842-4.53 | | 100 | 1.84 | 0.10375 |
| | Prediction Interval | | | | 0.17-22.39 | | | | |

**Table S9**. Heterogeneity Quantification in RCTs Evaluating Vitamin D Biomarkers and ASD (Funnel Plot Analysis).

| No. | Parameter | Value | 95% CI |
|---|---|---|---|
| 1 | $Tau^2$ | 0.9236 | 0.251-3.958 |
| 2 | Tau | 0.9610 | 0.501-1.989 |
| 3 | $I^2$ | 0.81 | 0.642-0.896 |
| 4 | H | 2.27 | 1.67-3.095 |
| **Test of Overall Efect: $t_8$ = 1.84 (p = 0.10)** | | | |

**Table S10**. Cochran's Q Test Results for Heterogeneity in RCTs on Vitamin D Biomarkers and ASD (Funnel Plot Analysis).

| No. | Q | d.f. | p-value |
|---|---|---|---|
| 1 | 41.35 | 8 | <0.001 |

**Table S11.** Effect Estimates and Study Characteristics of Randomized Controlled Trials (RCTs) on Vitamin D Biomarkers and ASD After Trim-and-Fill Correction.

| No. | Authors (year, country) | Study Design | log OR | OR | 95% CI | SE | Weight (%) | t | p-value |
|---|---|---|---|---|---|---|---|---|---|
| 1 | Aagaard et al. (2024, Denmark) | RCT | -0.2790 | 0.76 | 0.59–0.97 | 0.1268 | 9.5 | | |
| 2 | Saas et al. (2020, Denmark) | RCT | -0.2744 | 0.76 | 0.53–1.09 | 0.1839 | 9.4 | | |
| 3 | Javadfar et al. (2020, Iran) | RCT | 1.9726 | 7.20 | 1.63–31.71 | 0.7572 | 7.4 | | |
| 4 | Kerley et al. (2017, Ireland) | RCT | 1.6431 | 5.14 | 0.47–56.90 | 1.2236 | 5.5 | | |
| 5 | Mazahery et al. (2019, New Zealand) | RCT | 1.5854 | 4.88 | 1.35–17.65 | 0.6558 | 7.9 | | |
| 6 | Mazahery et al. (2020, New Zealand) | RCT | 1.6558 | 5.25 | 0.94–29.18 | 0.8764 | 6.9 | | |
| 7 | Moradi et al. (2018, Iran) | RCT | 1.6287 | 5.09 | 1.45–17.92 | 0.6414 | 7.9 | | |
| 8 | Sandboge et al. (2023, Finland) | RCT | -0.9169 | 0.40 | 0.17–0.94 | 0.4363 | 8.7 | | |
| 9 | Tuovinen et al. (2021, Finland) | RCT | 0.8456 | 2.33 | 1.19–4.56 | 0.3427 | 9.0 | | |
| | Filled: Moradi et al. (2018, Iran) | Filled | -1.9884 | 0.14 | 0.04-0.48 | 0.6414 | 7.9 | | |
| | Filled: Kerley et al. (2017, Ireland) | Filled | -2.0028 | 0.13 | 0.01-1.48 | 1.2236 | 5.5 | | |
| | Filled: Mazahery et al. (2020, New Zealand) | Filled | -2.0154 | 0.13 | 0.02-0.74 | 0.8764 | 6.9 | | |
| | Filled: Javadfar et al. (2020, Iran) | Filled | -2.3322 | 0.10 | 0.02-0.43 | 0.7572 | 7.4 | | |
| | Random Effects Model (REM) | | | 0.97 | 0.37-2.57 | | 100 | | |
| | Prediction Interval (PI) | | | | 0.04-24.77 | | | | |

**Table S12**. Heterogeneity Metrics for Trim-and-Fill Adjusted Meta-Analysis of RCTs on Vitamin D Biomarkers and ASD Risk.

| No. | Parameter | Value | 95% CI |
|---|---|---|---|
| 1 | $Tau^2$ | 1.9780 | |
| 2 | Tau | 1.4064 | |
| 3 | $I^2$ | 0.82 | |
| 4 | H | 2.27 | |
| **Test of Overall Efect: $t_{12}$ = 0.07 (p = 0.95)** | | | |

**Table S13**. Cochran's Q Test for Heterogeneity in Trim-and-Fill Adjusted RCT Meta-Analysis on Vitamin D Biomarkers and ASD.

| No. | Q | d.f. | p-value |
|---|---|---|---|
| 1 | 65.21 | 12 | <0.001 |

**Table S14.** Summary of Observational Studies Evaluating Vitamin D and ASD Risk: Odds Ratios and Study Characteristics.

| No. | Authors (year, country) | Study Design | log OR | OR | 95% CI | SE | Weight (%) | t | p-value |
|---|---|---|---|---|---|---|---|---|---|
| **1** | Egorova et al. (2020, Sweden) | Observational | -0.2339 | 0.79 | 0.58–1.08 | 0.1586 | 8.8 | | |
| **2** | Schmidt et al. (2019, U.S.A.) | Observational | 0.8097 | 2.25 | 1.01–5.00 | 0.4080 | 7.5 | | |
| **3** | Sourander et al. (2023, Finland) | Observational | 0.4689 | 1.59 | 1.06–2.41 | 0.2095 | 8.6 | | |
| **4** | Vinkhuyzen et al. (2017, Netherlands) | Observational | 0.8548 | 2.42 | 1.09–5.07 | 0.3921 | 7.6 | | |
| **5** | Windham et al. (2020, U.S.A.) | Observational | -0.2255 | 0.79 | 0.49–1.30 | 0.2489 | 8.4 | | |
| **6** | Raghavan et al. (2017, U.S.A.) | Observational | 0.7960 | 2.22 | 1.26–3.90 | 0.2882 | 8.2 | | |
| **7** | Wu et al. (2018, China) | Observational | 1.1822 | 3.68 | 2.03–5.24 | 0.2419 | 8.5 | | |
| **8** | Altun et al. (2018, Turkey) | Observational | 0.0028 | 1.00 | 0.06–16.76 | 1.4369 | 2.6 | | |
| **9** | Arastoo et al. (2018, Iran) | Observational | 2.5075 | 12.27 | 1.45–103.9 | 1.0898 | 3.7 | | |
| **10** | Bener et al. (2017, Qatar) | Observational | 0.8947 | 2.36 | 1.74–3.44 | 0.1739 | 8.7 | | |
| **11** | Bičíková et al. (2019, Czech Rep.) | Observational | 0.2177 | 1.24 | 0.48–3.22 | 0.4856 | 7.0 | | |
| **12** | Petruzzelli et al. (2020, Italy) | Observational | 2.3330 | 10.31 | 1.96–54.22 | 0.8470 | 4.8 | | |
| **13** | Saad et al. (2015, Egypt) | Observational | 2.5719 | 13.1 | 7.2–23.8 | 0.3050 | 8.1 | | |
| **14** | Shom et al. (2024, India) | Observational | -1.1073 | 0.34 | 0.14–0.78 | 04382 | 7.3 | | |
| | Random Effects Model (REM) | | | 2.05 | 1.13-3.72 | | 100 | 2.62 | 0.02134 |
| | Prediction Interval (PI) | | | | 0.26-16.09 | | | | |

**Table S15**. Heterogeneity Metrics in Observational Studies ($I^2$, $\tau^2$, H).

| No. | Parameter | Value | 95% CI |
|---|---|---|---|
| **1** | $Tau^2$ | 0.82 | 0.344-2.653 |
| **2** | Tau | 0.91 | 0.587-1.629 |
| **3** | $I^2$ | 0.89 | 0.825-0.925 |
| **4** | H | 2.95 | 2.39-3.646 |
| **Test of Overall Efect: $t_{13}$ = 2.62 (p = 0.02)** | | | |

**Table S16**. Cochran's Q Test Results for Observational Studies.

| No. | Q | d.f. | p-value |
|---|---|---|---|
| **1** | 113.29 | 13 | <0.001 |

**Table S17**. Effect Estimates and Study Characteristics from the Randomized Controlled Trial (RCT) by Hendren et al. (2016) on Vitamin $B_{12}$ Supplementation and Autism Spectrum Disorder (ASD): Forest Plot Data Summary.

| Subgroup | Events (n) | Total (n) | Odds Ratio (OR) | 95% CI |
|---|---|---|---|---|
| B12 Group | 14 | 27 | 0.52 | 0.32 - 0.71 |
| Placebo Group | 6 | 23 | 0.26 | 0.10 - 0.48 |
| Overall Estimate | 20 | 50 | 0.39 | 0.06 - 1.00 |

**Notes:**

- Meta-Analytic Parameters:
    - Model Used: Inverse Variance (IV), Random Effects.
    - Summary Measure: Odds Ratio (OR).
    - 95% CI (Confidence Interval): Computed for each subgroup and overall.

**Table S18.** Heterogeneity Quantification Metrics for the RCT on Vitamin $B_{12}$ Biomarkers and ASD: $Tau^2$, $I^2$, and H Indices.

| No. | Parameter | Value | 95% CI |
|---|---|---|---|
| **1** | $Tau^2$ | 0.023 | 0.001-0.412 |
| **2** | Tau | 0.152 | 0.032-0.641 |
| **3** | $I^2$ | 0.70 | 0.264-0.897 |
| **4** | H | 1.83 | 1-3.845 |
| **Test of Overall Efect: $t_1$ = 2.24 (p = 0.03)** | | | |

**Table S19**. Cochran's Q Test Results Assessing Heterogeneity in the RCT on Vitamin $B_{12}$ and ASD Outcomes.

| No. | Q | d.f. | p-value |
|---|---|---|---|
| **1** | 3.33 | 1 | 0.07 |

**Table S20.** Effect Estimates and Study Characteristics of Observational Studies on Vitamin $B_{12}$ and ASD (Forest Plot Data Summary).

| No. | Author (year, country) | Study Design | log OR | OR | 95% CI | SE | Weight (%) | t | p-value |
|---|---|---|---|---|---|---|---|---|---|
| **1** | Schmidt et al. (2019, USA) | Observational | 0.8097 | 2.25 | 1.01–5.00 | 0.4080 | 9.0 | | |
| **2** | Sourander et al. (2023, Finland) | Observational | 0.4689 | 1.59 | 1.06–2.41 | 0.2095 | 9.4 | | |
| **3** | Vinkhuyzen et al. (2018, Netherlands) | Observational | 0.1467 | 1.16 | 0.90–1.49 | 0.1286 | 9.5 | | |
| **4** | Altun et al. (2018, Turkey) | Observational | 0.4653 | 642.44 | 209.45–1970.51 | 0.5718 | 8.5 | | |
| **5** | Bener et al. (2017, Qatar) | Observational | 0.8947 | 2.36 | 1.74–3.44 | 0.1739 | 9.4 | | |
| **6** | Li et al. (2024, China) | Observational | 0.7077 | 2.03 | 1.43–2.88 | 0.1786 | 9.4 | | |
| **7** | Nesa et al. (2022, Bangladesh) | Observational | 1.4279 | 4.17 | 1.72–10.11 | 0.4518 | 8.9 | | |
| **8** | Zou et al. (2024, China) | Observational | 1.0045 | 2.73 | 1.55–4.81 | 0.2889 | 9.3 | | |
| **9** | Eshawi et al. (2024, Libya) | Observational | 0.1324 | 1.13 | 0.36–3.62 | 0.5888 | 8.5 | | |
| **10** | Yektaş et al. (2019, Turkey) | Observational | 2.1340 | 8.45 | 3.67–19.45 | 0.4254 | 8.9 | | |
| **11** | Raghavan et al. (2017, USA) | Observational | 0.7960 | 2.22 | 1.26–3.90 | 0.2882 | 9.3 | | |
| | Random Effects Model (REM) | | | 3.77 | 1.17-12.12 | | 100 | | |
| | Prediction Interval (PI) | | | | 0.08-188.31 | | | 2.53 | 0.02962 |

**Table S21**. Heterogeneity Metrics for Vitamin $B_{12}$ Observational Studies (Funnel Plot Analysis).

| No. | Parameter | Value | 95% CI |
|---|---|---|---|
| **1** | $Tau^2$ | 2.73 | 1.265-9.511 |
| **2** | Tau | 1.65 | 1.125-3.084 |
| **3** | $I^2$ | 0.93 | 0.89-0.953 |
| **4** | H | 3.73 | 3.021-4.594 |
| **Test of Overall Efect: $t_{10}$ = 2.62 (p = 0.02)** | | | |

**Table S22**. Cochran's Q Test Results for Heterogeneity in Vitamin $B_{12}$ Observational Studies.

| No. | Q | d.f. | p-value |
|---|---|---|---|
| **1** | 138.80 | 10 | <0.001 |

**Table S23.** Trim-and-Fill Adjusted Effect Estimates in Vitamin B12 Observational Studies on ASD.

| No. | Author (year, country) | Study Design | log OR | OR | 95% CI | SE | Weight (%) | t | p-value |
|---|---|---|---|---|---|---|---|---|---|
| **1** | Schmidt et al. (2019, USA) | Observational | 0.8097 | 2.25 | 1.01–5.00 | 0.4080 | 6.6 | | |
| **2** | Sourander et al. (2023, Finland) | Observational | 0.4689 | 1.59 | 1.06–2.41 | 0.2095 | 6.8 | | |
| **3** | Vinkhuyzen et al. (2018, Netherlands) | Observational | 0.1467 | 1.16 | 0.90–1.49 | 0.1286 | 6.8 | | |
| **4** | Altun et al. (2018, Turkey) | Observational | 0.4653 | 642.44 | 209.45–1970.51 | 0.5718 | 6.5 | | |
| **5** | Bener et al. (2017, Qatar) | Observational | 0.8947 | 2.36 | 1.74–3.44 | 0.1739 | 6.8 | | |
| **6** | Li et al. (2024, China) | Observational | 0.7077 | 2.03 | 1.43–2.88 | 0.1786 | 6.8 | | |
| **7** | Nesa et al. (2022, Bangladesh) | Observational | 1.4279 | 4.17 | 1.72–10.11 | 0.4518 | 6.6 | | |
| **8** | Zou et al. (2024, China) | Observational | 1.0045 | 2.73 | 1.55–4.81 | 0.2889 | 6.8 | | |
| **9** | Eshawi et al. (2024, Libya) | Observational | 0.1324 | 1.13 | 0.36–3.62 | 0.5888 | 6.4 | | |
| **10** | Yektaş et al. (2019, Turkey) | Observational | 2.1340 | 8.45 | 3.67–19.45 | 0.4254 | 6.6 | | |
| **11** | Raghavan et al. (2017, USA) | Observational | 0.7960 | 2.22 | 1.26–3.90 | 0.2882 | 6.8 | | |
| | Filled: Zou et al. (2024, China) | | 0.0176 | 1.02 | 0.58-1.79 | 0.2889 | 6.8 | | |
| | Filled: Nesa et al. (2022, Bangladesh) | | -0.4058 | 0.67 | 0.27-1.62 | 0.4518 | 6.6 | | |
| | Filled: Yektaş et al. (2019, Turkey) | | -1.1119 | 0.33 | 0.14-0.76 | 0.4254 | 6/6 | | |
| | Filled: Altun et al. (2018, Turkey) | | -5.4432 | 0.00 | 0.00-0.01 | 0.5718 | 6.5 | | |
| | Random Effects Model (REM) | | | 1.71 | 0.47-6.26 | | 100 | | |
| | Prediction Interval (PI) | | | | 0.01-274.08 | | | 0.89 | 0.39 |

**Table S24**. Heterogeneity Metrics Post Trim-and-Fill Correction (Vitamin $B_{12}$ Observational Studies).

| No. | Parameter | Value | 95% CI |
|---|---|---|---|
| **1** | $Tau^2$ | 5.1657 | 1.265-9.511 |
| **2** | Tau | 1.65 | 1.125-3.084 |
| **3** | $I^2$ | 0.95 | 0.89-0.953 |
| **4** | H | 3.73 | 3.021-4.594 |
| **Test of Overall Efect: $t_{14}$ = 0.89 (p = 0.39)** | | | |

**Table S25**. Cochran's Q Test Post Trim-and-Fill Correction (Vitamin $B_{12}$ Observational Studies).

| No. | Q | d.f. | p-value |
|---|---|---|---|
| **1** | 276.09 | 14 | <0.001 |

**Table S26.** Effect Estimates and Study Characteristics of Observational Studies Evaluating Homocysteine and ASD Risk: Forest Plot Summary.

| No. | Author (year, country) | Study Design | log OR | OR | 95% CI | SE | Weight (%) | t | p-value |
|---|---|---|---|---|---|---|---|---|---|
| **1** | Schmidt et al. (2019, USA) | Observational | -0.0002 | 1.00 | 0.34–2.94 | 0.5503 | 8.6 | | |
| **2** | Altun et al. (2018, Turkey) | Observational | -0.0145 | 0.97 | 0.06–16.19 | 1.4280 | 1.8 | | |
| **3** | Li et al. (2024, China) | Observational | 0.7700 | 2.16 | 1.50–3.11 | 0.1860 | 21.1 | | |
| **4** | Nesa et al. (2022, Bangladesh) | Observational | 1.3383 | 3.81 | 1.65–8.81 | 0.4273 | 11.7 | | |
| **5** | Zou et al. (2024, China) | Observational | 0.8488 | 2.33 | 1.27–4.30 | 0.3111 | 15.8 | | |
| **6** | Eshawi et al. (2024, Libya) | Observational | 1.0759 | 2.93 | 1.22–7.05 | 0.4475 | 11.1 | | |
| **7** | Yektaş et al. (2019, Turkey) | Observational | 1.4475 | 4.25 | 2.12–8.53 | 0.3552 | 14.1 | | |
| **8** | Raghavan et al. (2017, USA) | Observational | -0.0740 | 0.92 | 0.44–1.96 | 0.3811 | 13.2 | | |
| **9** | Li et al. (2022, China) | Observational | 2.4668 | 11.77 | 1.21–114.78 | 1.1613 | 2.6 | | |
| | Random Effects Model (REM) | | | 2.30 | 1.43-3.70 | | 100 | | |
| | Prediction Interval (PI) | | | | 0.82-6.41 | | | 4.03 | 0.00381 |

**Table S27**. Quantifying Heterogeneity in Observational Studies of Homocysteine and ASD: $Tau^2$, $I^2$, and Related Metrics.

| No. | Parameter | Value | 95% CI |
|---|---|---|---|
| **1** | $Tau^2$ | 0.15 | 0-1.758 |
| **2** | Tau | 0.39 | 0-13.26 |
| **3** | $I^2$ | 0.47 | 0-0.754 |
| **4** | H | 1.37 | 1-2.015 |
| **Test of Overall Efect: $t_{14}$ = 0.89 (p = 0.39)** | | | |

**Table S28.** Cochran's Q-Test for Between-Study Heterogeneity in Observational Studies on Homocysteine and ASD.

| No. | Q | d.f. | p-value |
|---|---|---|---|
| **1** | 15.08 | 8 | 0.06 |

**Table S29.** Summary of Randomized Controlled Trials Investigating the Effects of Homocysteine Modulation on Autism Spectrum Disorder (ASD) Outcomes.

| No. | Author (year, country) | Study Design | OR | 95% CI |
|---|---|---|---|---|
| **1** | Wink et al. (2016, USA) | RCT | 1.71 | 0.37–7.86 |
| **2** | Mazahery et al. (2020, New Zealand) | RCT | 5.25 | 0.94–29.18 |
